# Current Perspectives on the Beneficial Effects of Soybean Isoflavones and Their Metabolites for Humans

**DOI:** 10.3390/antiox10071064

**Published:** 2021-06-30

**Authors:** Il-Sup Kim

**Affiliations:** Advanced Bio-Resource Research Center, Kyungpook National University, Daegu 41566, Korea; 92kis@hanmail.net

**Keywords:** soybean-derived isoflavones, physiological effect, gene regulation, health benefit mechanism

## Abstract

Soybeans are rich in proteins and lipids and have become a staple part of the human diet. Besides their nutritional excellence, they have also been shown to contain various functional components, including isoflavones, and have consequently received increasing attention as a functional food item. Isoflavones are structurally similar to 17-β-estradiol and bind to estrogen receptors (ERα and ERβ). The estrogenic activity of isoflavones ranges from a hundredth to a thousandth of that of estrogen itself. Isoflavones play a role in regulating the effects of estrogen in the human body, depending on the situation. Thus, when estrogen is insufficient, isoflavones perform the functions of estrogen, and when estrogen is excessive, isoflavones block the estrogen receptors to which estrogen binds, thus acting as an estrogen antagonist. In particular, estrogen antagonistic activity is important in the breast, endometrium, and prostate, and such antagonistic activity suppresses cancer occurrence. Genistein, an isoflavone, has cancer-suppressing effects on estrogen receptor-positive (ER+) cancers, including breast cancer. It suppresses the function of enzymes such as tyrosine protein kinase, mitogen-activated kinase, and DNA polymerase II, thus inhibiting cell proliferation and inducing apoptosis. Genistein is the most biologically active and potent isoflavone candidate for cancer prevention. Furthermore, among the various physiological functions of isoflavones, they are best known for their antioxidant activities. *S*-Equol, a metabolite of genistein and daidzein, has strong antioxidative effects; however, the ability to metabolize daidzein into *S*-equol varies based on racial and individual differences. The antioxidant activity of isoflavones may be effective in preventing dementia by inhibiting the phosphorylation of Alzheimer’s-related tau proteins. Genistein also reduces allergic responses by limiting the expression of mast cell IgE receptors, which are involved in allergic responses. In addition, they have been known to prevent and treat various diseases, including cardiovascular diseases, metabolic syndromes, osteoporosis, diabetes, brain-related diseases, high blood pressure, hyperlipidemia, obesity, and inflammation. Further, it also has positive effects on menstrual irregularity in non-menopausal women and relieving menopausal symptoms in middle-aged women. Recently, soybean consumption has shown steep increasing trend in Western countries where the intake was previously only 1/20–1/50 of that in Asian countries. In this review, Ihave dealt with the latest research trends that have shown substantial interest in the biological efficacy of isoflavones in humans and plants, and their related mechanisms.

## 1. Introduction

In general, substances that interfere with the endocrine system are known as endocrine disruptors. Most are artificial chemicals. These estrogen disruptors (including pesticides such as dichlorodiphenyltrichloroethane (DDT), polychlorinated biphenyls (PCBs), and bisphenol A) not only cause reproductive problems, but also induce cancer and obesity [1]. In contrast, phytoestrogens are estrogen-like non-steroid substances found in plants (hence the prefix “phyto”) and are effective in preventing cancer, arteriosclerosis, osteoporosis, menopausal symptoms, and obesity [2]. Twenty different types of phytoestrogens are known so far, including lignans (secoisolariciresinol, matairesinol, pinoresinol, and lariciresinol), isoflavones (genistein, daidzein, glycitein, and formononetin), coumestans (coumestrol), and prenylflavonoids [3]. Such phytoestrogens are abundant in grains, vegetables, and fruits. Soybeans contain particularly high amounts of isoflavones, making it advantageous that soybeans are easy to consume. Barley, sunflower seeds, lentils, arrowroot, broccoli, and cauliflower also contain isoflavones [4]. The most well-known isoflavones are genistein, daidzein, formononetin, biochanin A, and coumestrol. Genistein is the most abundant isoflavone in legumes. Among legumes, soybeans contain the highest amount of genistein (26.8–102.5 mg/100 g dry weight), followed by arrowroot (12.6 mg/100 g dry weight) [5]. Compared to soybeans, other food sources contain very small amounts of genistein. In addition to genistein (40–60%), daidzein (30–50%) and glycitein (12–13%) are other well-known isoflavones [5].

Isoflavones protect plants exposed to the external environment. Although the recommended daily intake of isoflavones has not yet been established, the FDA recommends an intake of 50 mg per day, which is considered to be safe [6]. Currently, there is a lack of information on the side effects of ingesting high concentrations of isoflavones. Isoflavone intake is exceptionally high in Asian countries; China, Japan, and South Korea have a daily soy isoflavone intake in the range of 25–50 mg, in contrast to the United States and European countries, where the average daily intake is less than 2 mg [6].

Asian countries consume isoflavones in tofu, tempeh, miso, natto, and cheonggukjang; in Western countries, it is mainly consumed in the form of dairy substitutes, such as soy milk, soy cheese, and soy yogurt [7,8]. Isoflavones occur in food as either sugar-bound glycosides or as aglycones, without a sugar molecule; aglycones are known to be more bioavailable than glycosides [9]. In non-fermented foods like tofu and bean sprouts, isoflavones mostly exist as glycosides; however, in fermented foods like doenjang (soybean paste) and cheonggukjang, large amounts of isoflavones are broken down, and these foods thus provide highly bioavailable isoflavones [10]. Chemically, isoflavones are compounds of the flavonoid family with a 3-phenylchromone skeleton that are abundant in soybeans, particularly in its hypocotyl [11].

Isoflavones show structural similarity to the female hormone estrogen as well as similar biological activities, and consequently they are called phytoestrogens [12]. Phytoestrogens have been found to serve as a potential alternative therapy for hormone-dependent diseases, including cancer, menopausal syndrome, cardiovascular diseases, and osteoporosis. Isoflavones also play a role in strengthening the bones by increasing bone density [13]. There are 12 types of soy isoflavones, classified into glycosides and aglycones according to their chemical structure. Of them, genistein and daidzein in particular been attracting attention for their anti-cancer and antioxidative effects, and are thus hypothesized to have the potential to prevent and treat not only various types of cancers, but also various lifestyle diseases [9,13]. They have been found to inhibit the action of enzymes involved in the proliferation of cancer cells; several studies have reported their ability to inhibit cancers such as prostate cancer. In addition, studies have shown that they bind weakly to estrogen receptors and inhibit the development of breast cancer cells that require estrogen activity [6,14]. Isoflavones exhibit weak estrogen activity and can also prevent bone loss by promoting bone cell proliferation. Various studies have reported their role in the prevention of osteoporosis caused by menopause and aging [6,15]. Due to the recent increasing interest in soybean extracts, isoflavones are now being used as a material in food medicines for a variety of commercialized products such as functional health supplements, cosmetics, and in beverages [5,12]. Therefore, aim of this review is to highlight the novel functionality of various soybean-derived moleculesthat have recently received a great deal of attention, and to suggest future directions for study through the examination of isoflavone characteristics, and beneficial and adverse effects, with a special focus on health functionality assessment. Furthermore, I hope that this review will be helpful to a wide range of readers, including persons or researchers broadly interested in natural science, agriculture, food science, or medicinal foods.

## 2. Search Strategy

The study results used in this review have been obtained from the only PubMed database based on studies conducted in the last five years since 2016 (2016–2020) with the exception of five articles located searched on Google Scholar. The search keywords were “soybean-derived isoflavone and beneficial effects.” The related mechanism diagram was reconstructed based on results reported since 2010, unless there were significant changes, to produce a simple diagram for readers to easily understand. It should be noted that some pieces of information cited in this review are based on results obtained prior to 2016 if there is little information. Experimental strategies, including model, isoflavone type, and assay (in vitro and in vivo) and approach (observation and clinic) methods are summarized in Table S1. The chemical structures of the major classes of isoflavones and their metabolites were determined using ChemSpider. All figures were drawn directly on the basis of the references cited in the paper. Furthermore, although only positive results from the authors’ subjective perspective have been described in this review, an effort was made to deliver as much information as possible from an objective standpoint.

## 3. Content, Structure, and Metabolism of Isoflavones

### 3.1. Isoflavone Content in Soybean

According to the USDA Database for the Isoflavone Content of Selected Foods (Release 2.1) published by the US Department of Agriculture (http://doi.org/10.15482/USDA.ADC/1324538 or http://www.ars.usda.gov/nutrientdata/isoflv; accessed on 1 January 2021), soybean is the only type of beans, in comparison to other beans, to contain high levels of isoflavones [16]. The isoflavone content for soybeans varies slightly between countries, as in 100 g soybean it was found to be 118.28 mg in China, 130.65 mg in Japan 159.98 mg in the US, and it was highest in Korea at 178.81 mg. The global average was 154.53 mg [16]. This indicates that the isoflavone content of soybeans varies according to the cultivar and growth conditions; but nevertheless, it generally make up 0.2–0.3% of the dry weight. The daily recommended intake of isoflavones is around 40–50 mg/day, and to achieve this 25 g of boiled soybean or 100 g of common tofu should be consumed daily [5].

### 3.2. Isoflavone Biosynthesis and Regulation

**Figure 1:**
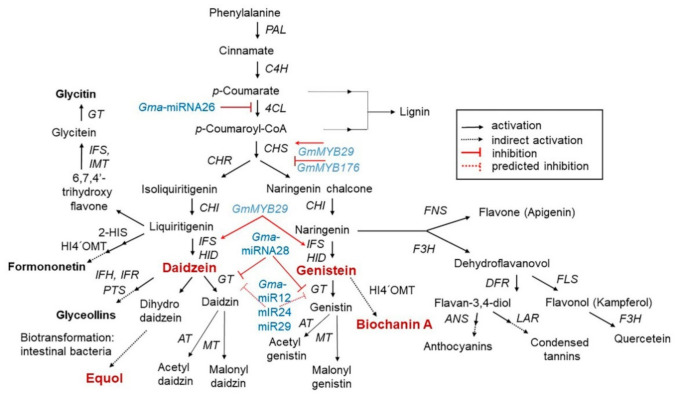
Schematic representation of the phenylpropanoid pathway, showing the key intermediates and enzymes associated with isoflavone biosynthesis, and partial regulation pathway. Isoflavones are biosynthesized via a phenylalanine-dependent pathway in the presence of a wide range of enzymes. Regarding regulation, GmMYB29 activates chalcone synthase (CHS) expression, whereas GmMYB176 inhibits CHS expression. Furthermore, Gma-miRNA26, Gma-miRNA269, and Gma-miRNA28 suppress the gene expression or enzymatic activity of 4-coumarate-CoA ligase (4CL), isoflavone synthase (IFS), and glucosyltransferase (GT), respectively. Dotted lines represent the unclear role of miRNA in the pathway [18,19]. This isoflavone biosynthetic pathway is adapted from “USDA Database for the Isoflavone Content of Selected Foods Release 2.0 (http://www.ars.usda.gov/nutrientdata; accessed on 1 March 2021).

The isoflavone biosynthetic pathway is divided into several branches that generally share common substrates resulting in strong and regulated flux channeling. Isoflavones have a distinct group of plant secondary metabolites that are produced from the phenylpropanoid pathway [17]. The precursor in the multistep pathway of isoflavone biosynthesis is the amino acid L–phenylalanine, which in the initiating step is stripped of its amine group to generate cinnamic acid after non-oxidative deamination via the enzyme phenylalanine ammonia lyase (PAL). Then, cinnamic acid is translated into *p*-coumaryol CoA by 4–hydroxylase (C4H) and 4–coumarate CoA ligase (4CL). The first critical enzymes involved in isoflavone biosynthesis are from the multigenic chalcone synthase (CHS) family, although not all are expressed in the seeds at detectable levels. Of all the CHS homologous copies, CHS7 and CHS8, which are seed specific in soybean, have the highest expression levels in seeds and they catalyze the conversion of *p*–coumaryol CoA into naringenin chalcone. Chalcone isomerase (CHI) and chalcone reductase (CHR) are the other important enzymes that are required for isoflavone synthesis. CHI converts chalcones to flavanones and CHR is required for daidzein and glycitein formation (Figure 1) [18,19].

Isoflavone synthase (IFS) is a cytochrome P450 monooxygenase that is important for distinguishing between isoflavone-producing and isoflavone-lacking plants. The soybean genome harbors two IFS genes (IFS1 and IFS2) with 14 different amino acids. The two IFS isoforms catalyze the first reaction of isoflavone biosynthesis to form 2-hydroxyisoflavanone, which in turn is dehydrated to form daidzein and genistein naturally or by intervention of 2-hydroxyisoflavanone dehydratase (HID) [20]. Soybean synthesizes daidzein and genistein in the cytoplasm, which are additionally glycosylated into daidzin and genistin by UDP-glucose:isoflavone 7-*O*-glucosyltransferase, or malonylated into malonyldaidzin/genistin by malonyl-CoA:isoflavone 7-*O*-glucoside 6″-*O*-malonyltransferase [21]. Malonylated products accumulate in vacuoles in which daidzein and genistein are directly secreted to the root or rhizosphere through membrane transport by ATP-binding cassette-type transporters or are indirectly secreted into apoplasts as isoflavone glucosides. This process is mediated by isoflavone conjugates-hydrolyzing beta-glucosidase (ICHG) present in the apoplasts [22]. In addition to isoflavone biosynthesis, the phenylpropanoid pathway is involved in the synthesis of lignins, stilbene, phlobaphenes, proanthocyanidins, and anthocyanins through specific branches [23,24]. The differences in isoflavone accumulation among several soybean varieties result from genetic and environmental interactions, which regulatory mechanisms remain unclear.

All steps of the isoflavone biosynthesis pathway are regulated by other genes that determine the accumulation patterns of other compounds in the pathway [18]. Currently, five microRNAs (Gma–miRNA12, Gma–miRNA24, Gma–miRNA26, Gma–miRNA28, and Gma–miRNA29) are known to regulate isoflavone biosynthesis. The differential expression of Gma-miRNA26 and Gma-miRNA28, and their corresponding target genes (Glyma.10g197900 and Glyma.09g127200) were directly related to the total isoflavone content [18]. In contrast, some MYB transcription factors involved in the regulation of the isoflavone biosynthesis pathway have been identified in soybean [25]. For example, the R1-type MYB transcription factor GmMYB176 affects isoflavone synthesis by regulating chalcone synthase 8 (CHS8) expression. R2R3-type MYB transcription factors (GmMYB39 and GmMYB100), are believed to negatively regulate isoflavone biosynthesis by inhibiting the expression of structural biosynthesis genes [25,26]. Recently, the R2R3–type MYB transcription factor GmMYB29 (Glyma.20g209700) was found to activate a promoter related to IFS2 and CHS8 [25,26]. Overexpression of GmMYB29 in hairy roots increased, whereas isoflavone levels were reduced upon its suppression [26]. Thus, a better understanding of the isoflavone biosynthesis mechanism may aid in the development of novel plant varieties based on molecular breeding.

### 3.3. Structure and Type of Isoflavones

**Figure 2:**
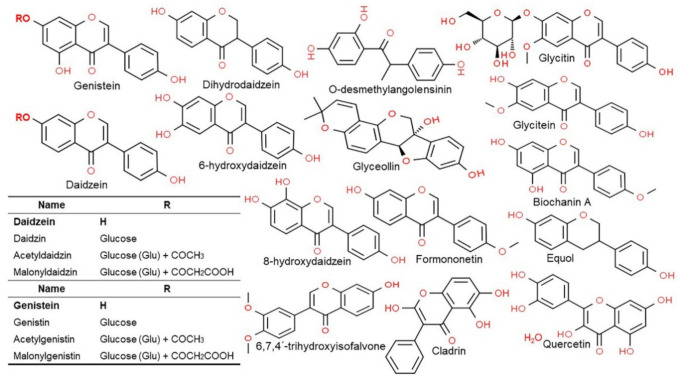
Chemical structures of the major classes of isoflavones and their metabolites. Structures were drawn using the ChemSpider. See the genistein and daidzein backbone in bold before reviewing respective molecules.

Isoflavones are compounds with a 3-phenylchrome structure, and the best known are genistein, daidzein, formononetin, biochanin A, and coumestrol. Soy isoflavones are divided into glycosides and aglycones depending on their chemical structures. Twelve isoflavones have been identified in soybean, including three aglycones—genistein, daidzein, and glycitein—and their corresponding glycosides daidzin, genistein, and glycitin, as well as malonyl glycoside and acetyl glycoside. The glycosides in soybean are often found as malonyl glycoside. The other forms include the glycosides of daidzin and genistin, which are daidzein and genistein, respectively, with a carbohydrate attached to C7 (7-*O*-glucosides) and 6′-*O*-acetyl daidzin and 6′-*O*-acetylgenistin, which have a carbohydrate attached to C6 (6′-*O*-acetylglucosides). There are also 6′-*O*-malonyl daidzin and 6′-*O*-malonyl genistin (6′-*O*-malonylglucosides) (Figure 2) [5]. For absorption in the intestine the glycosides with a polysaccharide attached (7′-*O*-glucosides, 6′-*O*-acetylglucosides, and 6′-*O*-malonylglucosides) are converted to aglycones, which are free isoflavones without the sugar, by an enzyme called β-glucosidase secreted by the intestinal microflora [9,27]. The chemical structure of soy isoflavones resembles estrogen and they have an affinity for the estrogen receptor. Such isoflavone compounds are mainly distributed in the epicotyl and hypocotyl of the plant as mentioned above, while their contents vary greatly among the different cultivars and even within the same cultivar, variation in content has also been reported to be depended on growth conditions [13,28].

### 3.4. Functionality of Isoflavones in Plants

**Figure 3:**
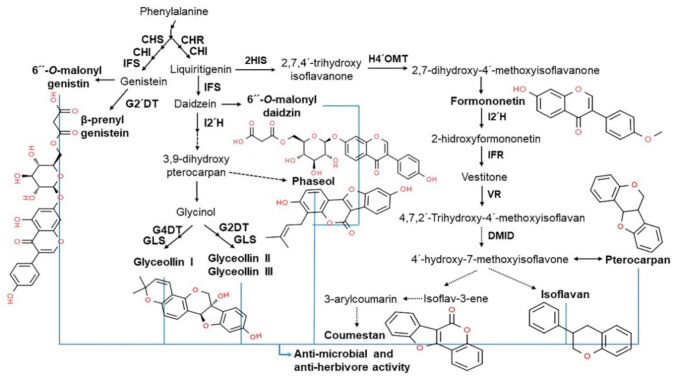
Relationship between isoflavone products and antimicrobial activity. Phenylalanine-derived biosynthetic isoflavone products, including 6″-*O*-malonylgenistein, glyceollins (I, II, and III), coumestan, isoflavan, and pterocarpan, are capable of antimicrobial and antiherbivore activity for defense against abiotic stress, such as pathogens [29,30,31,32].

In plants, isoflavones play an important role in plant-microbial interactions, including defense and symbiosis. In plant defense systems, isoflavones serve as stress-resistant mediators with antioxidant activity that help neutralize reactive oxygen species (ROS) induced by stressful conditions. They are precursors of phytoalexins, a classic plant metabolite with antibacterial and antiviral effects and/or antiherbivore activities that protect plants from pathogen infections (Figure 3) [26,29,30,31,32]. One of the most well-known soybean phytoalexins is prenylated pterocarpans derived from glyceollins and daidzein, which are released in response to pathogens, such as *Phytophthora sojae* and *Macrophomina phaseolina* [33]. In addition to their role in the plant defense system, isoflavones are known to have health benefits for humans, including the prevention and amelioration of various diseases including cancer, cardiovascular disease, neurologic disorders, climacteric syndrome, obesity, inflammation, and aging, due to its phytoestrogen and antioxidant properties [13,34]. Recently, isoflavones were reported as desirable ingredients in bean-based infant formulas [35]. Therefore, the various benefits of soybean-derived isoflavones to plants and humans highlight their importance and the need for more relevant research (especially in plants).

As aforementioned, isoflavone biosynthesis is believed to be regulated by environmental conditions [5]. For example, high concentrations of heavy metals can suppress plant growth and development. Environmental factors can directly or indirectly influence various organism structures and processes through the production of ROS [36]. In particular, vanadium compounds have a negative impact on plant growth and development, as they can bind to phosphates of enzymes and inhibit their activation, such as protein kinases, ribonucleases, or ATPases [37,38,39]. Despite these known negative effects and toxicity, vanadium compounds have also been observed in vivo as promising plant-induced substances that affect the production and exudation of secondary metabolites, and have been reported as potential therapeutic agents for several diseases [37]. Moreover, some vanadium compounds can activate genes and enzymes involved in the phenylpropanoid pathway, thereby facilitating flavonoid production and release [38].

The complex surrounding environment of plants is continuously evolving. Thus, to protect themselves from harmful substances, plants are equipped with a natural immune system, divided into acquired and induced systemic resistance [40,41]. In plants, phytoestrogens do not function as hormones, instead they have an important role as phytoalexins, small molecule compounds with antifungal, antibacterial, antiviral, and antioxidant properties that are produced in response to environmental stress and pathogenic attack [5]. Among these compounds, isoflavones play a crucial role in plant-microbe interactions, such as rhizobia-legume symbiosis and defense responses in legumes, including soybeans [42,43]. In plant defense, isoflavones act as a precursor of antibacterial and/or antiherbivore activities of phytoalexins that protect the plant from pathogen infection [44,45]. One of the most well-known phytoalexins in soybean is prenylated pterocarpans derived from glyceollins and daidzein, which are induced in response to pathogens such as *P. sojae* and *M. phaseolina* [33,46]. For example, daidzein is metabolized to produce glyceollins, which are important elements in the defense mechanism against fungal pathogens. Concerning symbiosis, isoflavones are signaling molecules that contribute to the formation of nitrogen-fixing root nodules in legumes [47]. In addition to these functions in plants, isoflavones (such as daidzein and genistein) are released into rhizospheres—a small area around legume roots—where they will serve as signaling molecules for rhizobia to form nodules in the roots [22,48]. Therefore, isoflavones play an important role in biological communication with soil microbes. Research on the functions of isoflavones in plants is lacking compared with that available in humans; therefore, this topic warrants more attention and investigation.

## 4. The Role of Isoflavones in Humans

### 4.1. Absorption and Metabolism

Isoflavones that exist as glycosides are converted to genistein, daidzein, and other metabolites in a free-state for absorption in the intestine by the gastric acid and the microbial enzyme β-glucosidase [9]. Isoflavones are lipophilic, so their absorption and metabolism employ the same pathways as other lipophilic nutrients. Hence, isoflavones that are absorbed in the intestine join the chylomicron to be transferred by blood along the lymph nodes and delivered to all cells in the body [13,15]. When isoflavones reach the liver, they are stored in the form of glucuronide or sulphate then released to the small intestine through the bile for circulation in the gastrointestinal tract. When the circulation is over, isoflavones are excreted mainly through urine. The ingested isoflavones are almost completely excreted after two days, with approximately half being excreted within 12 h [49,50].

### 4.2. Estrogen-Like Effect and Activity

**Figure 4:**
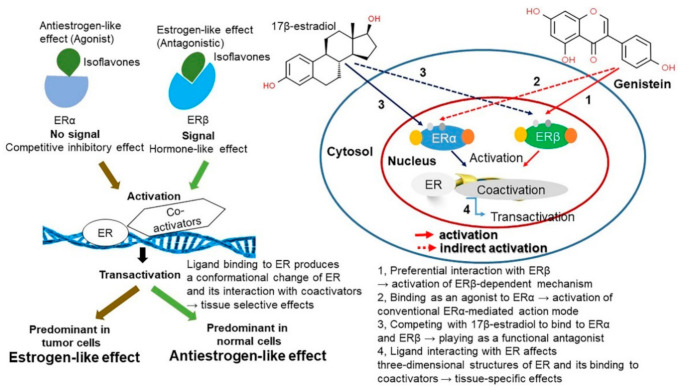
Phytoestrogenic activity of soybean isoflavone mediated by estrogen receptor beta (ERβ) [51], predicted action mode of 17β-estradiol, and potential interaction of isoflavones in humans [52]. If isoflavones (genistein) with activity similar to that of 17β-estradiol bind to ERβ, it will promote a signal with an estrogen-like effect (antagonist), leading to the development and differentiation of normal cells by upregulating ER and coactivators. In contrast, isoflavones binding to ERα act as a predominant effector with an estrogen-like effect (agonist) in tumor cells. Figure adapted from Huser et al. [52].

Soy isoflavones resemble the female hormone estrogen and they bind to the estrogen receptor (ER) based on their inherent binding affinity (Figure 4) [14,51,52,53]. Although isoflavones have a lower affinity than estrogens, they play an identical role by acting as an ER-β selective ligand. Isoflavones inhibit testosterone 5-α reductase, which converts testosterone into an activated form, by inhibiting the aromatase enzyme (estrogen synthetase), thus maintaining the balance of estrogen/androgen [54]. Soy isoflavones, through an activity similar to estrogens, also promote vasodilation and improve blood flow by increasing the production of nitrogen monoxide, and this has an anti-arteriosclerosis effect. In addition, via the ER-mediated pathways, soy isoflavones influence the growth and survival of nerve cells, synaptic plasticity, and brain function (Figure 4) [51,52,53]. Isoflavones can regulate the plasma testosterone level by stimulating the production of sex-hormone binding globulins that bind with the free sex hormones in the serum to render them inactive [15,52].

### 4.3. Neuroprotective Effect and Improvement of Cognitive Impairment

The brain governs all body behaviors and maintains homeostasis, i.e., it keeps the heart rate, blood pressure, blood concentration, and body temperature constant, and is responsible for cognition, emotion, memory, and learning. The brain is the one organ that does not stop growing even as one ages, and the more it is used, the tighter the connections between the cells, thus, if managed properly even with aging, it can function as effectively as during youth. Therefore, while it is important to increase blood circulation for the health of the body and to build muscles, it is also required for brain health. Furthermore, quitting smoking and regular physical activity can also increase blood flow to the brain and maintaining an appropriate weight, as well as eating plenty of vegetables, fruits, olive oils, beans, fish, and low-fat milk can also help to improve brain health [55,56].

**Figure 5:**
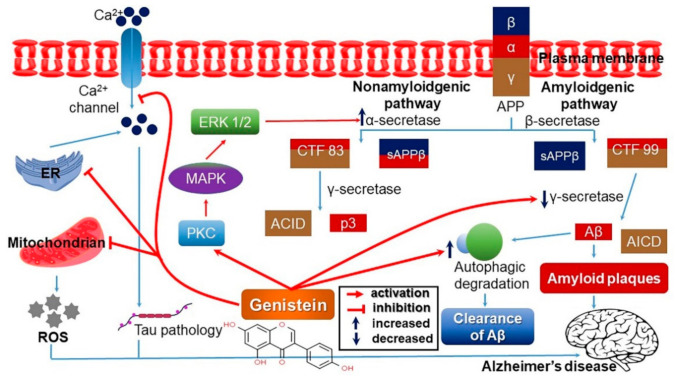
Proposed mechanism underlying the neuroprotective effects of genistein against neuropathological insults of Alzheimer’s disease. In the amyloidogenic pathway, cleavage of amyloid precursor protein (APP) by β- and γ-secretases produces Aβ peptides, whereas in the non-amyloidogenic pathway, cleavage of APP by α- and γ-secretases provoke the formation of p3 and APP intracellular domain (AICD). Protein kinase C (PKC) signaling pathway including mitogen-activated protein kinase (MAPK) and extracellular signal-regulated kinase 1 and 2 (ERK1/2), activated by genistein, activates the non-amyloidogenic pathway of APP cleavage by increasing α-secretase activity. In the amyloidogenic pathway, genistein inhibits the formation of Aβ peptides by decreasing γ-secretase. Further, genistein reduces Tau-mediated pathology by decreasing intracellular calcium concentrations, inhibiting oxidative stress-mediated neuronal damage and death by inhibiting the reactive oxygen species (ROS) released from mitochondria and promoting autophagic clearance of aggregate-prone proteins [57]. Figure adapted from Uddin and Kabir [57].

Aging affects all tissues and organs including the brain. In post-menopausal women, neurodegenerative disease incidence is significantly higher than in young women. Genistein upregulates protein kinase C (PKC), activating the cyclic AMP (cAMP)/cyclic AMP response element-binding protein (CREB)—brain-derived neurotrophic factor (BDNF)—tyrosine receptor kinase B (TrkB)—phosphatidylinositol 3-kinase (PI3K)—serine/threonine kinase Akt (also known as protein kinase B) signaling pathway mediated by PKC, and regulating the activity of α- and β-secretase. Based on this, genistein blocks neuro-toxicity to protect the nerve cells (Figure 5) [57,58,59]. However, the general behavior of mice exposed to isoflurane showed better learning abilities as genistein led to more intense fear responses, and spatial learning ability was also markedly improved as genistein restored the ability that had been degraded by isoflurane [60].

Isoflavones restore the cell membrane composition and increase the density of dendrites that are crucial for signaling while promoting the production of nerve growth factors. They also regulate acetylcholine synthesis and release and maintain its level, and restore the level of dopamine release, to facilitate neurotransmitter activities and enhance the function of nerve cell membranes. Hence, isoflavones significantly boost cholinergic function in the hippocampus, suppress the level of oxidative stress, and increase the expression of extracellular signal-regulated kinase (ERK), brain-derived neurotrophic factor (BDNF), and cAMP response element binding (CREB) protein, thereby exhibiting a neuroprotective effect against scopolamine-induced memory impairment [55,61]. In detail, the daidzein metabolite 6,7,4-trihydroxyisoflavone (6,7,4-THIF) improves learning and memory in the cholinergic nervous system and affects signaling pathways. To evaluate the effect of 6,7,4-THIF on scopolamine (which causes memory and cognitive impairment)-induced learning and memory impairment, experimental mice were used in a maze and passive avoidance tests. The results indicated that scopolamine impaired memory and decreased the spontaneous alteration score in the Y maze test, but 6,7,4-THIF (5 mg/kg) administration increased the spontaneous alteration score. In addition, 6,7,4-THIF significantly inhibited acetylcholinesterase (which hydrolyzes acetylcholine to disrupt normal cholinergic nerve signaling) and thiobarbituric acid reactive substance (lipid oxides and aging indicators) in the hippocampus of scopolamine-induced mice and significantly improved the brain-derived neurotrophic factors, which promote hippocampal neurogenesis in the brain, responsible for memory and learning, and fluorescent CREB protein, which is essential for the formation of long-term and spatial memories in the brain (Table S2) [61]. The study revealed that 6,7,4-THIF improves cognitive dysfunction caused by scopolamine and improves learning and memory ability through the activation of the cholinergic system and phosphorylated CREB (p-CREB)/BDNF signaling pathways.

A preclinical study using experimental mice was conducted to investigate the neuroprotective effects of isoflavone in memory disorders caused by scopolamine and to clarify its mechanisms. The results showed that 40 mg/kg of isoflavone improved the object localization task and the cognitive ability of the mice in the Morris water maze test. Isoflavone remarkably enhanced the cholinergic function in the hippocampus and suppressed the level of oxidative stress, while the expression levels of the extracellular signal-regulated kinase, BDNF, and CREB were increased (Table S2) [55]. This suggests that isoflavone is a good candidate for the treatment of neurodegenerative diseases such as Alzheimer’s as it shows neuroprotective effects on the cognitive dysfunction caused by scopolamine.

Reports on isoflavones improving cognitive function in the early postmenopausal phase were first published in 2001; studies in this field regarding the pros and cons of isoflavones are still under way. Many studies have reported that menopausal women who consumed 60 mg of isoflavones per day showed improvements in cognitive function and overall mood. These results are understandable, considering the antioxidant activity and estrogen-like functions of isoflavones. In particular, genistein has a high antioxidative effect and can pass through the cerebrovascular barrier, providing protection against various oxidative stresses that affect the brain (Table S2) [5,12,57,62]. However, studies have shown that long-term isoflavone intake may decrease cognitive abilities; therefore, further research is needed. It has been reported that the antioxidant activity of isoflavones inhibits the phosphorylation of Tau proteins related to Alzheimer’s [57]; thus, it may be expected to have positive effects on preventing dementia. It is also believed that genistein promotes serotonin transmission in the brain, and its antidepressant effects are thought to help overcome depression in menopausal women [12]. Small steps in daily life can help maintain brain health and reduce the risk of developing dementia later in life. Constantly stimulating the brain and eating soy foods that are good for the brain can help maintain a person’s health life.

### 4.4. Antidepressant Effects

One of the biggest global health concerns in recent years has been depression, as more than 300 million people, or approximately 4% of the global population, suffer from this disease, and it is more prominent among the elderly, women, and adolescents. Depression causes problems in the ability of the brain’s ability to control emotions, but the cause cannot be explained by a single theory. However, the most well-known theory is the monoamine hypothesis, which states that a deficiency in the monoamine neurotransmitters, norepinephrine, serotonin, and dopamine, can cause depression [63,64]. The World Health Organization (WHO) recently defined depression as a disease and disorder, rather than controllable emotions, and emphasized the need for effective treatment [65].

Genistein has been shown to have effects like those of antidepressant drugs in a behavioral despair test. The effects of genistein on depression were analyzed according to the period of ingestion (long-term or once) and the results showed that the long-term ingestion of genistein resulted in effects that were similar to those of antidepressant drugs. In the forced swim test and tail flick test, genistein was shown to decrease the immobility time in a concentration-dependent manner and in particular, 45 mg/kg genistein reduced the immobility time by 48.3% and 45.6%, respectively, in each test, implying similar effects to the antidepressant drug imipramine. Furthermore, long-term genistein ingestion was shown to increase the monoamine level in the brain and dose-dependently inhibit monoamine oxidase activity. These results indicate that genistein plays the role of an antidepressant drug in the serotonin system and that the 5-hydroxytryptamine (serotonin) 1A (5-HT1A) receptor plays a crucial role in the antidepressant effect of genistein [66]. In addition, a study analyzing the prevalence of depression according to the ingestion of bean products and isoflavone intake, showed that the prevalence of gestational depression was significantly reduced. A cross-sectional study was conducted to investigate the prevalence of depression depending on the consumption of soy foods (tofu, miso soup, tofu products, fermented soybeans, and boiled soybeans) and isoflavones in 1745 pregnant women. According to the amount of consumption, the subjects were divided into four groups and the prevalence of depression by each soy food and isoflavone intake was analyzed. The results showed that the consumption of soy foods and isoflavones reduces depression during pregnancy. The prevalence of depression decreased by 28% with tofu, 26% with tofu products, 43% with fermented soybeans, 27% with boiled soybeans, 35% for miso soup, and 37% for isoflavones (Table S2) [67]. These results confirm that the consumption of tofu, miso soup, tofu products, fermented soybeans, and boiled soybeans reduces the risk of developing depression during pregnancy.

Improving physical health generally has a good effect on mental health and can help overcome depression. Regular exercise creates natural endorphins, which can act as antidepressants, and acquiring sufficient levels of vitamins and minerals via a regular diet can also help to reduce depression. Furthermore, eating foods that increase serotonin in the body are also beneficial, and thus physical and mental health may be improved by consuming soybeans along with egg whites, low-fat cheese, pumpkin, and sesame seeds, which are rich in tryptophan, and the raw materials for serotonin.

### 4.5. Anti-Obesity Effects

The WHO in 1996, defined obesity as a “disease that requires long-term treatment” [68] and as a major cause of eight types of cancers (colorectal, endometrial, ovarian, prostate, kidney, breast, liver, and gallbladder) [69]. Obese people also have a four times higher risk of developing coronary artery disease, six times higher risk of stroke, twelve times higher risk of high blood pressure, and a six times higher risk of diabetes than those with a normal weight. Obesity is also known to have a negative effect on overall health, including physical, mental, psychological, and social health. Consequently, increased levels of obesity result in rapid increases in mortality rates and obesity is thus considered one of the most important diseases that mankind must overcome in 21st century [70,71]. It is caused by a combination of environmental factors, such as irregular eating habits, excessive calorie intake, and lack of exercise, as well as genetics [72]. As obesity causes metabolic syndromes and related disorders, it must be actively managed to maintain a health life. Many studies have shown that soy ingredients and foods are effective in preventing obesity [73].

Genistein and daidzein have anti-obesity effects and genistein acts directly on the adipocytes or preadipocytes, and consequently a protective effect against obesity and obesity-related metabolic diseases is produced [74]. In a concentration-dependent manner, genistein converted the lipids from a droplet form with large distribution to a bundle form during the differentiation of adipocytes, and this lipid accumulation pattern was similar to that in brown fat cells. Genistein reduced the expression of the genes (ACC, FASN, Fabp4, HSL, chemerin, and resistin) that are active in white fat cells while increasing the expressions of the genes (CD-137 and UCP1) for the characteristics of brown fat cells (Table S3). Hence, genistein modified the gene expression profile and mitochondrial function in adipocytes to engage in the transition from white to brown or beige adipocytes. Through such mechanisms, the development of beige adipocytes is promoted [75,76].

**Figure 6:**
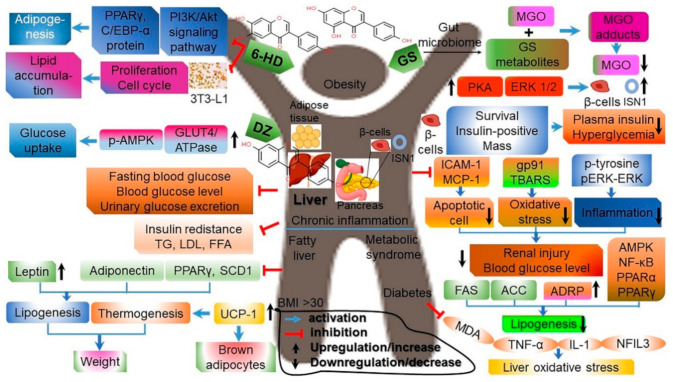
Anti-diabetes and anti-obesity activity of daidzein (DZ), genistein (GS) and its metabolite (6-hydroxydaidzein; 6-HD) through decreased lipogenesis, liver oxidative stress, hyperglycemia, urinary glucose secretion, insulin tolerance, and weight, as well decreased levels of triacylglycerol (TG), low-density lipoprotein (LDL), free fatty acids (FFAs), fasting blood glucose (FBG), and plasma insulin. Genistein and daidzein activate the expression of extracellular signal-regulated kinase 1/2 (ERK1/2), Wnt/β-casein, tyrosine kinase inhibitor [p38 and Janus kinase 2 (JAK2)], 5′-adenosine monophosphate-activated protein kinase (AMPK) pathway. The upregulated effectors inhibit the expression of genes associated with peroxisome proliferator-activated receptor gamma (PPARγ), CCAAT/enhancer-binding proteins (C/EBPα, β and δ), SET-binding protein 1 (STEBp1), which lead to the downregulation of adipocyte genes involved in adipocyte cell differentiation and lipid accumulation [78]. ↑, enhancing; ↓, lowering; →, activation; ┤, inhibition. Figure adapted from Kim et al. [78].

Isoflavones play a key role in the AKT/mTORC1 pathway that is crucial in lipid metabolism and weight loss. Lipid production is reduced, and lipid decomposition is promoted when isoflavones increase gene expression regating to β-oxidation and ketogenesis that is suppressed by a high-fat diet while inhibiting the mTORC1 activity via the AKT signaling pathway [77]. Isoflavones thus help with weight loss and lipid decomposition by improving lipid metabolism. Isoflavones also decrease the expressions of genes (SREBP-1c, ACC, FAS, and PPARγ) that govern adipocyte production so as to reduce the weight, visceral fat, serum leptin level, and adipocyte size in both normal and obese mice (Figure 6; Table S3) [71,78,79].

Glucocorticoids play a critical role in glucose and lipid metabolism and lipid regulation, and it is an important factor in obesity control as it contributes to the advancement of visceral obesity, hyperlipidemia, dyslipidemia, and hypertension [80]. A recent study reported that in mouse adipocytes, 17β-estradiol inhibited 11β-hydroxysteroid dehydrogenase type 1 (11β-HSD1) regulation of glucocorticoids in a non-competitive manner. Genistein, with a structure similar to estradiol, was also reported to participate in producing an antiobesity effect. Genistein was shown to inhibit the activity of 11β-HSD1 in mouse adipose tissue and liver while inhibiting the activity of hexose-6-phosphate dehydrogenase (H6PD)/glucose-6-phosphate dehydrogenase (G6PD) that donate NADPH to 11β-HSD1 (Table S3). Notably, the inhibition of enzyme activity was time and dose dependent. This ultimately inhibits glucocorticoid biosynthesis in adipose tissue to have a preventive effect against obesity [81].

Oxidative stress accumulates during exercise. Ingesting isoflavones reduces oxidative stress, increases the activity of antioxidant enzymes, and decreases blood triglyceride levels. In addition, genistein induces the death of adipocytes through AMP-activated protein kinase (AMPK), inhibits adipocyte production, and suppresses the process by which adipocytes absorb glucose. A hormone called leptin is secreted by adipocytes, which acts on the brain and suppresses appetite, lowers blood sugar levels, and increases metabolic efficiency in order to control body weight. In people with obesity, leptin levels are high due to errors in this process. In such cases, since leptin cannot function properly, the body perceives leptin to be insufficient [82,83,84]. According to a study, leptin levels decreased after consistent intake of isoflavones; however, further research is needed to determine the exact mechanism. Further, one of the common complications of obese patients is fatty liver disease, resulting in liver dysfunction (increased levels of alanine aminotransferase [ALT] and aspartate aminotransferase [AST]) (Table S3). Studies have reported that consistent intake of isoflavones improves fatty liver disease and normalizes elevated AST and ALT levels [85,86,87].

As obesity is a disease in which several complications can occur, and is difficult to easily control with individual efforts, active obesity management is required to improve the quality of life and increase life expectancy. Obesity can be prevented and treated by managing eating habits, exercise, smoking, drinking, and stress, as well as the consumption of soy foods that help prevent obesity.

### 4.6. Effects on Improving Metabolic Syndrome

Metabolic syndrome is a condition with co-occurrence of three or more of the following conditions: increased body fat, high blood pressure, high blood sugar, and abnormal blood lipids; it can cause cerebral and cardiovascular diseases as well as diabetes. Since metabolic syndrome increases the risk of cardiovascular diseases by 2–3 times and the risk of diabetes by more than 3-fold, the prevention, management and treatment of metabolic syndrome is necessary to lower these risks [88,89]. Metabolic syndrome is becoming increasingly common, occurring in one in three adults in the United States and one in four adults in South Korea. Active lifestyle changes can delay or prevent metabolic syndrome and its associated diseases [90].

The incidence of irritable bowel syndrome (IBS) was reported to improve when supplements combining isoflavone and active vitamin D were taken. Since periodic changes in the levels of estrogen affect female IBS patients and activated vitamin D is involved in the activity of estrogen receptors in intestinal smooth muscles, it was thought that ingestion of isoflavones and vitamin D could alleviate IBS symptoms. Thus, 40 mg of isoflavone and 50,000 international unit (IU) of activated vitamin D supplement, either separately or in combination, were given to 100 female IBS patients for 6 weeks, and indicators such as TNF-α, NF-κB, and fecal serine protease were measured. NF-κB (a transcription factor) is an important factor in triggering mucosal inflammation, and high levels of TNF-α (an inflammatory cytokine) induces the production and release of other inflammatory cytokines that induce NF-κB activation and increase intestinal permeability. Fecal serine protease is also an indicator of intestinal epithelial tissue permeability. If intestinal permeability is increased, it can cause an inflammatory reaction due to the invasion of pathogens. In the study, after 6 weeks, all indices increased in the placebo group; however, in the groups that received at least one type of supplement, there were significant decreases in all indices. The group that took both supplements showed the greatest decrease in these indices, with NF-κB being significantly reduced by half (Table S3) [91]. These results indicate that the combined intake of isoflavone and active vitamin D can help improve gastrointestinal disorders such as IBS by lowering the levels of cytokines that cause inflammation in the intestine, thus reducing intestinal permeability.

Genistein, in particular, has also been reported to improve the symptoms of inflammatory bowel disease. A preclinical study was conducted to confirm the effect of genistein on inflammation in dextran sodium sulfate (DSS)-induced colitis. Genistein was found to restore colon length and body weight by inhibiting the weight loss and colon length reduction caused by colon inflammation. In addition, it improved colitis symptoms by reducing both the number and percentage of M1 macrophages in DSS-induced colitis. Genistein was also found to reduce the levels of inflammatory cytokines (IL-6, TNF-α, and MCP-1) and improve inflammation by mediating T-cells (Table S3) [92]. These results show that genistein improves the severity of colitis through multiple pathways, such as reducing the number of T cells, levels of inflammatory cytokines, and number and percentage of M1 macrophages.

In addition, it has been reported that a diet deficient in isoflavones increases the risk of constipation. In a study, mice were fed with food either containing isoflavones (experimental group) or without isoflavones (control), for one week; the intestinal transmission function was evaluated by calculating the moisture content in the excrement sample, intestinal length and propulsion. Changes in the gut microbiota were observed to investigate the effects of isoflavone on the gastrointestinal tract. There was no significant difference in food intake between the experimental and control groups, but the fecal pellet number and fecal moisture content of the control group decreased by half compared to the experimental group (Table S3). In addition, the intestinal transit rate decreased by about 10%, and symptoms of constipation were seen. I found that the risk of constipation and metabolic diseases increased as the composition of the intestinal microflora changed, and the Firmicutes/Bacteroidetes ratio, an intestinal health evaluation index related to two major phyla of bacteria involved in the metabolism of undigested food, increased. Further, the number of *Desulfovibrio* bacteria was relatively increased, and oxidation of acid salts was inhibited by the hydrogen sulfide produced by these bacteria; thus, cell respiration was impeded, leading to apoptosis and chronic inflammation [93]. Based on these results, it was suggested that soy foods rich in isoflavones are essential to the diet, since a diet deficient in isoflavones affects gut health by altering gut activity and gut microbial balance.

Metabolic syndrome is closely related to overweight and obesity, low physical activity, as well as a condition called insulin resistance. A lifelong commitment to a healthy lifestyle can prevent metabolic syndrome. Having at least 30 min of physical activity each day, consuming adequate amounts of vegetables, fruits, low-fat protein, and whole grains, and limiting the intake of saturated fats and salts are beneficial. In addition, it is good to take a step further and maintain intestinal health, such as by habitual consumption of soy foods to prevent metabolic syndromes [94].

### 4.7. Improvements of Blood Pressure

High blood pressure, also referred to hypertension, affects approximately 30% of adults in South Korea and can occur for a variety of reasons. In the winter when the daily temperature varies by more than 10 °C, the number of patients with heart or cerebrovascular diseases increases. This is because when the body is exposed to cold air, the sympathetic nerves are affected and the blood vessels contract; therefore, patients with high blood pressure should take extra care in the winters [95]. Hypertension can be improved by changes to a person’s lifestyle, such as avoiding stress, quitting smoking, reducing alcohol intake, and getting at least 30 min of proper exercise every day [96]. According to the 2018 U.S. Hypertension Guidelines, losing 1 kg of body weight can lower systolic blood pressure by more than 1 mmHg; hence, maintaining an appropriate weight is important [97]. In addition, it is also necessary to reduce fat intake and consume less salty foods. Vegetables have low levels of sodium (responsible for an increase in blood pressure) and high levels of potassium (responsible for lowering blood pressure); therefore, a vegetarian diet can help patients with hypertension [88].

It has been shown that isoflavones contribute to blood pressure homeostasis by interacting with calcium. Based on studies that show that calcium and estrogen regulate vascular health, a randomized double-blind, placebo-controlled experiment was conducted to determine the mediating effects of isoflavones and calcium on blood pressure [98]. Postmenopausal women were divided into two groups, where 136.6 mg of isoflavone or placebo was administered five times a week, and after 2 years, blood pressure, serum calcium level, and the amount of excreted isoflavone were measured. The results showed that isoflavones had a significant effect on blood pressure in a concentration-dependent manner. Isoflavones, depending on the calcium concentration, decreased systolic blood pressure by 17.7 mmHg at the maximum calcium concentration, but increased systolic blood pressure by 13.81 mmHg at the lowest concentration. However, isoflavone decreased diastolic blood pressure regardless of the calcium concentration (Table S3) [99,100]. These results suggest that at the highest calcium concentration, the isoflavones had a mediating effect by normalizing the systolic blood pressure; therefore, isoflavone and calcium levels are important for maintaining blood pressure homeostasis.

When menopausal women consumed 54 mg of isoflavones per day, menopausal symptoms, such as hot flashes, were alleviated and systolic/diastolic blood pressure was also reduced. Since there are various effective antihypertensive drugs already in use, isoflavones may not be considered a substitute for such drugs (Table S3). However, since estrogen is a natural antihypertensive agent, estrogenic isoflavones can also be effective in lowering blood pressure [12,101,102].

### 4.8. Improvement of Cardiac Function

Cardiovascular diseases are steadily increasing every year due to lifestyle changes and aging. The WHO reported that more than 75% of the death due to cardiovascular diseases could be prevented by lifestyle adjustments [96]. In recent years, cardiovascular diseases and major risk factors have tended to occur in people of younger age; therefore, it is important to consistently manage heart health from a young age. Cardiovascular risk factors include family history, age, smoking, alcohol, stress, and the excessive consumption of sugars and saturated fatty acids that cause obesity and dyslipidemia. In particular, trans fats increase total cholesterol and low-density lipoprotein (LDL) cholesterol levels; consequently, it is recommended that the consumption of meat, sugary snacks, ice cream, and fast food be reduced [103,104]. In contrast, vegetables and fruits, whole grains (brown rice and mixed grains), and legumes provide a variety of complex carbohydrates, fiber, potassium, vitamins, and antioxidants to lower blood pressure and improve sugar and lipid metabolism [105]. Therefore, eating fresh vegetables and fruits of various colors 2–3 times a day is recommended (200 g/day of fruits and vegetables) [105].

Genistein was shown to relieve the reduced cardiac function by transverse aortic constriction although it did not have an effect on cardiac function in normal mice. Genistein also reduced cardiac failure while inhibiting hypertrophy due to a pressure overload [106,107]. Thus, genistein could regulate the transformation of myofibroblasts and as a result play a critical role in suppressing fibrosis in endomyocardial fibrosis. In addition to genistein, daidzein also improves cardiovascular disease risk factors effectively. It influences the genotype of the estrogen receptor, which effectively reduces the level of plasma triglyceride and uric acid, thereby improving the cardiovascular disease risk factors. Such effects were more prominent in individuals with the GA genotype for Rsal polymorphisms of the estrogen receptor (ERβ) gene (Table S4) [108].

Hormone replacement therapy is known to help reduce cardiovascular risks in postmenopausal women [109]. A randomized, double-blind study of 200 perimenopausal women analyzed the risk of cardiovascular disease in women after they were given either 66 mg of isoflavone-containing soy protein or 15 g of soy protein alone for 6 months and data on their age, diabetes, smoking, blood pressure, and lipid profiles were collected. The results showed that metabolic markers and systolic blood pressure were significantly lower in the women that consumed soy protein-containing isoflavones. When cardiovascular risk was analyzed using the cardiovascular risk indicators, the risk of developing coronary heart disease within 10 years decreased by 27%, the risk of myocardial infarction by 37%, the risk of cardiovascular disease by 24%, and the risk of death by cardiovascular diseases by 42% (Table S4). These results confirmed the advantage of using isoflavones as a hormone replacement therapy in postmenopausal women, as the risk of cardiovascular disease in postmenopausal women reduced with the intake of isoflavone-containing soy protein more than with soy protein alone [110].

A study reported that ingesting 60 mg of isoflavones per day reduced cardiovascular diseases in menopausal women. Cardiovascular diseases are not only related to blood cholesterol levels, but also to the elasticity or relaxation of the arteries. When blood cholesterol is high and cholesterol accumulates on the arterial wall, the elasticity of the arterial wall decreases [6,111,112]. Endothelial cells that form the arteries secrete nitric oxide (NO), which plays a role in relaxing blood vessels [112]. Reportedly, when menopausal women ingested 80 mg of isoflavones per day, although blood vessel relaxation did not increase significantly, the stiffness of blood vessel walls was significantly reduced (Table S4) [95]. These results suggest that the ability of isoflavones to control cholesterol may have remarkable contributions to offer to the field of cardiovascular therapy.

The heart constantly provides the body with oxygen, blood, and nutrients, and is necessary for life. However, as cardiovascular mortality is increasing worldwide, effective heart management is important. Cardiovascular disease can be prevented through changes in dietary habits and regular exercise. A diet containing vegetables, fruits, and soybeans as opposed to a meat-oriented diet could prevent cardiovascular disease and lead to a healthier lifestyle.

### 4.9. Protection against Liver Damage

As the largest organ in our body, the liver weighs 1.2–1.5 kg in adults and plays an important role in processing and storing substances in our body. It is estimated that 1 in 3 Koreans have a fatty liver, which means that adipocytes occupy more than 5% of the total weight of the liver, and because there are no physical symptoms, its danger is generally underestimated. However, with the severity of a fatty liver, the normal cells of the liver lose their function and may progress to hepatitis and liver cirrhosis. Fatty liver is classified into alcoholic fatty liver and nonalcoholic fatty liver depending on the cause. Alcoholic fatty liver refers to a state in which fat is accumulated in the liver cells, and nonalcoholic fatty liver refers to a condition where one does not drink at all or drinks a small amount [20 g of alcohol (e.g., 360 mL of soju) per week for women and less than 40 g of alcohol per week for men] and has a lot of fat in the liver, similar to those who drink a lot of alcohol, and its main cause is the intake of high-calorie, high-fat foods, among various other causes [113]. A few ways to prevent fatty liver include abstaining from alcohol, weight loss, proper diet, and regular aerobic exercise.

In addition to the soybean proteins with known cholesterol lowering effects, soy isoflavones were also found to lower total cholesterol and LDL cholesterol while increasing high-density lipoprotein (HDL) cholesterol. In the liver, the main organ for LDL cholesterol synthesis, soy isoflavones inhibit lipid synthesis based on increased energy consumption and facilitate lipid decomposition, thereby playing an important role in effectively preventing the accumulation of lipid granules in the liver [114]. Non-alcoholic fatty liver disease (NAFLD) may advance from simple steatosis to severe liver failure [115]. A study on the effects of isoflavones on lipid accumulation in the liver was conducted in mice with NAFLD induced by a high-fat diet. Isoflavones were shown to reduce the liver index (the ratio of final body weight against liver weight) that had increased due to the high-fat diet as well as the serum biochemistry levels [alanine aminotransferase (ALT), aspartate aminotransferase (AST), LDL-cholesterol, triglycerides, and total cholesterol] and to increase free fatty acids and HDL-cholesterol that had been reduced by the alcohol (Table S4) [78]. While a high-fat diet also increased the expressions of sterol regulatory element binding proteins (SREBP-1c) and fatty acid synthase in the liver, isoflavones reduced the expression of such protein [116]. The results indicated that isoflavones could ameliorate liver steatosis and improve the advancement into NAFLD by inhibiting lipid production in the liver while facilitating lipid oxidation.

**Figure 7:**
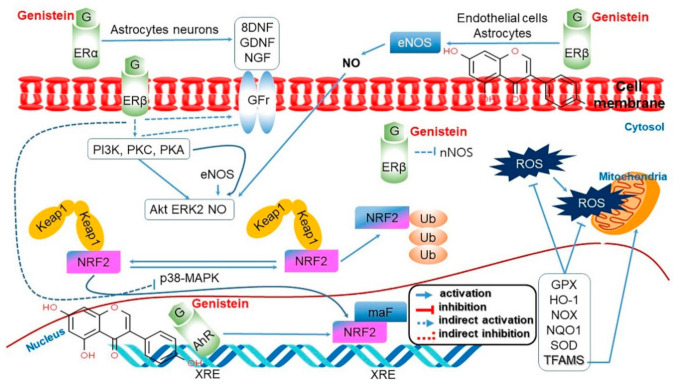
Antioxidant pathways activated by genistein (G) in cerebral ischemia to detoxify reactive oxygen species (ROS). Phosphorylation of nuclear factor erythroid 2 (Nrf2)-related substrate adapter protein Keap1 leads to either ubiquitination (Ub) and degradation of Nrf2 or release of Nrf2 for translocation to the nucleus, association with small musculoaponeurotic fibrosarcoma (Maf) protein, and activation of the transcription of several antioxidant genes (GPX, HO-1, NOX, NQQ1, and SOD) and Tfam through binding the antioxidant response element. The kinase pathways promoting this action (PI3K/Akt, PKC/ERK, and PKA/eNOS/NO) are stimulated by ERβ and its interaction with GFr through several intermediate proteins. This action mode is further processed by genistein-dependent growth factors from astrocytes and neurons and by the ERβ-induced NO generated in endothelial cells and astrocytes. Conversely, genistein inhibits degradation of Nrf2 by inhibiting p38 MAPK through activation of ERβ, which plays a role in inhibiting nNOS-induced ROS generation. Finally, genistein can enhance Nrf2-induced transcription by reinforcing AhR binding to the xenobiotic response element (XRE) [127]. Figure adapted from Schreihofer and Oppong-Gyebi [128].

The mechanisms of genistein action on chronic liver damage due to alcohol have also been recently identified. Genistein increases the lipid oxidation and peroxidation markers [heme oxygenase-1 (HO-1), catalase (CAT), superoxide dismutase (SOD), glutathione (GSH), and glutathione peroxidase (GPX)] that had been reduced by alcohol but decreased the inflammatory cytokines (NF-κB, p65, TGF-1β, COX-2, MCP-1, TNF-α, and IL-6) that had been increased by alcohol (Figure 7) [78,117,118]. Genistein provides antioxidant and anti-inflammatory activities to effectively protect the liver against chronic damage due to alcohol. Genistein also exhibits a protective effect against NAFLD induced by a methionine-choline deficient diet. In a study on type II diabetes using a 5-week-old male *db*/*db* mouse model, genistein was shown to relieve the oxidative and endoplasmic reticulum (EPR) stress and inactivate AMP-dependent phosphorylase although liver steatosis was not prevented. In addition, genistein regulated the gene expressions related to liver inflammation and fibrosis to inhibit lipid oxidation, inflammation, and fibrosis in the liver (Table S4) [119].

Patients with a fatty liver must follow a strict exercise and diet regimen. Moreover, obese people have a higher risk of liver damage from alcohol consumption than the general population. The most effective fatty liver treatments so far are weight loss, diet, and constant aerobic exercise. Consumption of soy foods that can prevent a fatty liver and may improve overall health.

### 4.10. Improvement of Renal Dysfunction

The kidneys act as water purifiers in the body because they are involved in metabolic processes, such as controlling salt and water levels, helping with the contraction and relaxation of blood vessels, and participating in vitamin D activation. However, kidney diseases can lead to serious situations in which the kidney can no longer filter waste products. This can lead to the accumulation of waste products, and swelling of the body due to the inability to urinate. The symptoms of kidney diseases appear only when more than 70% of the tissue is damaged; therefore, it is necessary to maintain a healthy lifestyle beforehand by consuming less salt, avoiding excessive protein intake, and exercising regularly. In addition, eating foods high in antioxidants helps the kidney by preventing chronic inflammation [120,121,122].

Genistein was shown to have a role in improving renal dysfunction due to ischemia/reperfusion (I/R) [123]. In lab mice with renal I/R injury, genistein improved renal dysfunction, cell damage, and apoptosis, while promoting cell proliferation. In other words, genistein regulated the expression of sirtuin 1 (SIRT1) engaged in the treatment of various diseases in the cells to improve apoptosis and promote the proliferation of cell nuclear antigens to prevent renal I/R injury by exhibiting cytoprotective effects using anti-apoptosis, antioxidation, and anti-inflammatory mechanisms [123]. In addition, the ingestion of soybean daidzein intake was reported to improve renal function in pre-hypertension menopausal women. In this study, soybeans as a food providing plant proteins were shown to better improve the renal function than milk which is rich in animal proteins (Table S4) [124].

Isoflavones protect the kidneys against diabetic nephropathy [125]. In a study using a mouse model for the condition, isoflavones were shown to reduce the expressions of Wnt4, β-catenin, and TGF-β1 that participate in the process of cell development, thereby decreasing urinary protein excretion and relieving renal histopathological damage. These effects of the isoflavones were not significantly different from those of the drug Losartan [126]. Genistein also protects the kidneys against diabetic renal failure through the regulation of oxidative stress and inflammation. Diabetes is a representative endocrine metabolic disorder and it accompanies hyperglycemia as it disrupts the metabolism of carbohydrates, proteins, and lipids. The oxidative stress caused by hyperglycemia may lead to inflammation and fibrosis and aggravate the condition to diabetic renal disorder [127]. Genistein was found to reduce the oxidative stress indicators for the kidney such as NRF2, HO-1, GPX, and SOD (Figure 7) [127,128], as well as reducing the inflammatory indices such as NF-κB, pIκBα, C-reactive protein, MCP-1, COX-2, and TNF-α [34,127]. The analysis of fibrosis-related indicators also showed an effect of genistein in inhibiting fibrosis (Table S4) [127].

Renal fibrosis is a histological feature that is common in renal aging and chronic renal diseases [129]. The epigenetic diet exhibits tissue protection and epigenetic regulation [130], but its function and mechanisms in renal fibrosis have not yet been identified. A preclinical study used experimental mice to determine the epigenetic regulatory properties of genistein on renal fibrosis function, and showed that genistein inhibits neurofibromatosis by restoring the epigenetic loss of klotho, a protein that strengthens the kidney, has antiaging properties, and inhibits fibrosis [129]. Genistein significantly restored Klotho loss and weakened the renal fibrosis-associated protein expression induced by a unilateral ureteral obstruction in the fibrous kidneys of mice. Mechanistically, genistein inhibited the histone 3 deacetylations of the Klotho promoter and induced DNA methyltransferases, DNMT1, and DNMT3a, normalizing DNA hypermethylation (Table S4) [129]. This suggests that genistein-induced Klotho restoration in the kidney is essential for the restoration of renal fibrosis function.

Kidneys may not show specific symptoms even if their normal function is reduced by 50% or more, so early detection of the damage is difficult. As kidneys cannot be recovered once they are damaged, it is important to maintain a healthy lifestyle that prevents kidney disease, this include quitting smoking, reducing alcohol intake, maintaining a normal weight, consuming less sodium, and eating soybeans.

### 4.11. Anti-Inflammatory Effects

Many diseases, such as rhinitis, enteritis, gastritis, pharyngitis, hepatitis, esophagitis, and bronchitis have the suffix ‘itis’ in their names as they are associated with inflammatory reactions. Inflammation is a type of defense reaction that occurs in the tissue of a living body in response to injury, and is typically characterized by pain (dolor), fever (calor), and redness (rubor). With extensive recent developments in immunology, the concept of innate immunity and its relationship with diseases have created major interest in the scientific community. Inflammatory reactions inevitably appear in response to all stimuli in daily life, but they differ in severity [131,132,133]. When our body is healthy or when the infection is weak, it can control the inflammatory response and recover to normal, but when the ability to control the inflammatory response is weakened, the damage caused by inflammation can be more severe, leading to disease [133]. The most important factor in reducing inflammation is diet. Acidic foods, such as meat, are high in cholesterol and saturated fatty acids that produce many ROS during digestion, which can cause inflammatory reactions throughout our body [134]. The consumption of alkaline foods that contain a lot of natural antioxidants or phytonutrients which remove ROS, such as fruits, vegetables, and legumes are recommended to reduce inflammatory reactions [135,136].

**Figure 8:**
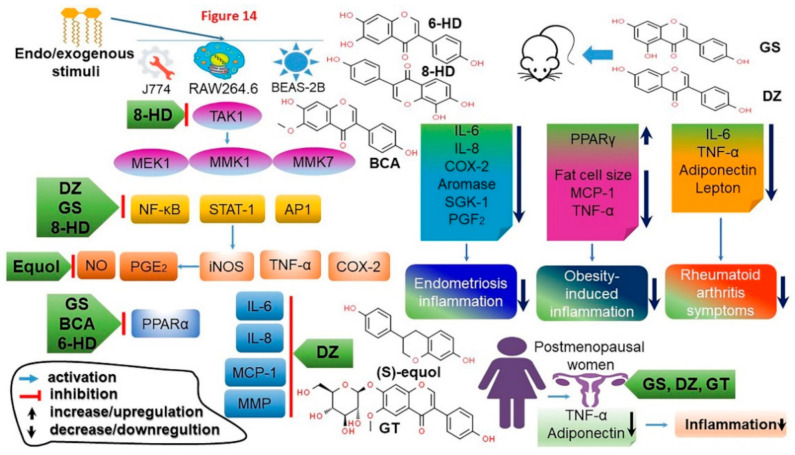
Inhibition of lipopolysaccharide (LPS)-induced inflammation in J774, RAW 264.7, and BEAS-2B cells by isoflavones [genistein (GS) and daidzein (DZ)] and their metabolites [6-hydroxydaidzein (6-HD), 8-hydroxydaidzein (8-HD), glycitein (GT), biochanin A (BCA), and *S*-equol]. 8-HD inhibits the expression of mitogen-activated protein kinase kinase (MEK1) and mitogen-activated protein kinase homologs MMK1 and MMK7 following inhibition of transforming growth factor β-activated kinase (TAK1). DZ, GS, and 8-HD inhibit the expression of the nuclear factor kappa B (NF-κB), signal transducer and activator of transcription 1 (STAT1), and activator protein 1 (AP1). *S*-equol blocks nitric oxide (NO) and prostaglandin E2 (PGE_2_) production. GS, BCA, and 6-HD inhibit the expression of the peroxisome proliferator-activated receptor-alpha (PPARα). Finally, DZ represses interleukin (IL)-6 and -8, monocyte chemoattractant protein-1 (MCP1), and matrix metalloproteinase (MMP) expression. In addition, GS and DZ reduce proinflammatory cytokines in different inflammatory manifestations in animal models, such as endometriosis, obesity, and rheumatoid arthritis models, via the regulation of multiple genes associated with inflammation. For postmenopausal women, aglycones (GS, DZ, and GT) reduce inflammation by downregulating tumor necrosis factor-alpha (TNF-α) and adiponectin [78]. Figure adapted from Hsiao et al. [78].

Isoflavones were shown to reduce the expression level of nitric oxide synthase 2 by inhibiting the production of NF-κB that increases due to the radiation and of pro-inflammatory cytokines (IL-6, TNF-α, IL-1β, and IFN-γ) (Figure 8) [34] while at the same time maintaining and thereby promoting the activity of an anti-inflammatory enzyme arginase-1. It was also shown to prevent the influx of radiation-induced neutrophils in the lungs [137,138]. Hence, by participating in the inflammatory reaction mechanisms in the lung tissue where they inhibit the radiation-induced macrophages, and the infiltration and activation of neutrophil, isoflavones provide protection against lung damage upon radiotherapy. In addition to isoflavones, the mechanisms by which daidzein inhibits acute lung injury due to lipopolysaccharides and exerts anti-inflammatory effects have been elucidated [139]. Daidzein could inhibit the expression of TLR4 and MyD88 proteins and NF-κB activity by reducing the levels of inflammatory cytokines TNF-α and IL-6 (Table S5) [34,140,141]. However, daidzein reduced iNOS expression and suppressed the activity of myeloperoxidase (MPO), a known reliability index for neutrophil infiltration [140,141]. The results indicated that isoflavones exhibit anti-inflammatory activity by inhibiting the signaling pathways related to inflammation.

Homocysteine induces vascular endothelial cell damage, and hyperhomocysteinemia is one of the risk factors of cardiovascular disease [142]. Studies have also recently reported that genistein has an inhibitory effect on endothelial cell damage. Genistein reduced the release of ROS and inhibited NF-kB activity while downregulating the cytokine IL-6 and ICAM-1 expressions and favoring the balance between the proliferation of endothelial cells and apoptosis, thereby shielding endothelial cells from homocysteine-induced inflammatory damage [143]. Genistein also exhibits a preventive effect against inflammatory diseases in the liver and the lungs [144]. D-Galactosamine increases the area of massive hemorrhagic necrosis due to inflammation and the inflammatory cell infiltration, and genistein was found to reduce the inflammatory damage of D-galactosamine. In addition, genistein inhibited the expressions of inflammatory mediators (iNOS and COX-2) and the production of inflammatory cytokines (TNFα and IL-1β) by blocking the inflammatory cytokine and enzyme IKK/NF-kB signaling pathway (Figure 8; Table S5) [34,145]. These results implicated that, since the NF-κB and MAPK signaling pathways are regarded as a promising therapeutic strategy in the development of anti-inflammatory drugs, genistein could be a potential therapeutic supplement for liver or inflammatory diseases.

Soy isoflavone exhibits antioxidant and anti-inflammatory properties [34], but the effect of isoflavones on chronic inflammatory bowel diseases are currently unknown. Recently, a preclinical study was conducted to explore the effects and the underlying mechanisms of soy isoflavones on dietary colitis caused by dextran sulfate sodium (DSS). Female mice were divided into four groups according to diet (control; basic diet + water; 5% DSS solution; 0.5% soy isoflavone; and 0.5% soy isoflavone + 5% DSS), and the inflammatory index was analyzed. The results indicated that soy isoflavone consumption significantly reduced TNF-α expression, which is increased by DSS, and alleviated the damage to the villi of the colon. In addition, isoflavone inactivated the myeloid differentiation primary response gene 88 (TLR4/Myd 88) signaling pathway, which is related to inflammation and is activated by DSS (Table S5) [146]. This shows that isoflavones can alleviate inflammation caused by DSS, improve antioxidant function, and alleviate intestinal barrier dysfunction.

Any stimulus inevitably leads to an inflammatory response. A high level of inflammation indicates that the body has a lot of inflammatory responses, which means that the immune system is out of balance. Since inflammation is the base cause various diseases, efforts are required to protect our body from inflammation. To increase immunity, proper exercise, adequate sleep, quitting smoking, and the consumption of soy foods that have anti-inflammatory effects are necessary to recover from inflammatory reactions and enjoy a healthy life.

### 4.12. Anticarcinogenic Activity through Gene Regulation

**Figure 9:**
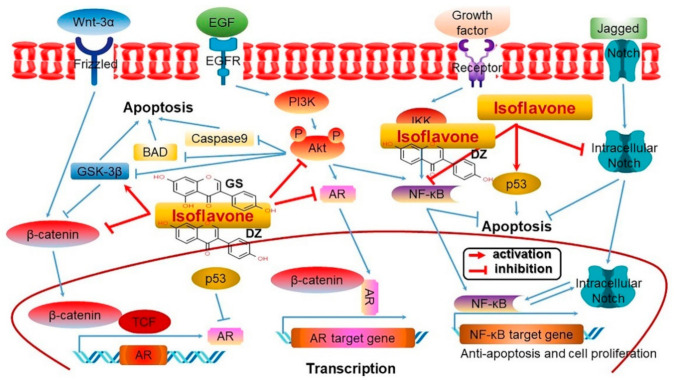
Cellular signaling mechanism associated with the activation of isoflavone-induced cancer cell apoptosis. Isoflavones activate glycogen synthase kinase 3 beta (GSK3β) and p53 associated with apoptosis. In contrast, they inhibit the expression of β-catenin, protein kinase B (Akt), androgen receptor (AR), nuclear factor kappa B (NF-κB), and intracellular Notch, which regulate tumor cell proliferation. In various cancer cells, altered conformational changes of target proteins caused by endo/exogenous stimuli, including mutations and amplifications of genes, or other defects, affect cellular signal that modulate apoptotic cell death. NF-κB, Akt, mitogen activated-protein kinase (MAPK), Wnt, Notch, p53, and AR pathways are commonly changed in various cancers. Among them, the NF-κB signaling pathway plays crucial roles in regulating cell growth and differentiation, apoptosis, inflammation, stress response, and other physiological processes including diabetes. Several important molecules in the NF-κB-mediated signaling pathway, such as NF-κB, inhibitor of NF-κB (IκB), and IκB kinase (IKK), regulate apoptotic signal transduction; however, NF-κB is the key protein in the pathway and has been recognized as the major culprit and a therapeutic target in cancer. The constitutive activation of NF-κB observed frequently in cancer cells is likely owing to the involvement of multiple other signal transduction pathways, such as tyrosine kinase, NF-κB-inducing kinase (NIK), and Akt pathways. NF-κB is known as a key modulator of apoptosis in various cell types. Activation of NF-κB inhibits apoptosis, whereas inhibition of isoflavones-induced NF-κB enhances apoptosis in human cancer cells, suggesting that isoflavones play critical roles in the apoptotic pathway by inhibiting NF-κB signaling [147]. Figure adapted from Kimet al. [8].

Soybeans contain a diverse array of health functional substances, the most representative of which are isoflavones. Soy isoflavones inhibit cancer cell growth by suppressing the expression of tyrosine kinase which has positive role in cancer cell growth, physiology, and division, while inducing apoptosis and controlling cell cycle [132]. In addition, soy isoflavones exhibit anticancer activity by inhibiting vasculogenesis which promotes cancer cell growth and metastasis as well as the migration to and activation in the nucleus of the transcription factor NF-κB (Figure 9) [147]. The anticancer activity also comes from the regulation of steroid hormone synthesis and metabolic enzyme systems [148,149,150,151,152,153,154,155]. For example, genistein is known to downregulate the expressions of Gli1 and CD44 while inhibiting the Gli1-related signaling pathway to play a unique regulatory role in cancer stem cell characteristics [156]. Genistein also increases the level of peptide phosphate IGEGpTpYGVVYK that influences the CDKs (CDK1, CDK 2, and CDK 3) related to the cell cycle, and regulates protein expression that is essential for DNA replication. As shown, genistein exerts an anticancer effect by inducing cell cycle arrest and apoptosis as it regulates DNA cell damage response and cell cycle regulation (Table S6) [157].

Among the different isoflavones, genistein is seen as having various antitumor effects [158,159]. Genistein was shown to inhibit lung cancer cell proliferation by using an anticancer mechanism that activates the caspase 3/9 signaling pathway to induce lung cancer cell apoptosis while activating the expression of miR-27, an activator of Wnt signaling pathway, and reducing the expression of the MET protein, a receptor of lung cancer cell growth factor [160]. Hence, genistein inhibits the proliferation of lung cancer cells by regulating the miR-27 and MET signaling pathways. Another study showed that genistein inhibited the expression of FoxM1 genes that regulate cancer cell apoptosis, whereby lung cancer metastasis was prevented. Moreover, genistein promoted not only its own anticancer activity but also that of another anticancer substance by enhancing the effects of cisplatin, an anticancer substance (Table S6) [161].

In cervical cancer caused by human papillomavirus (HPV) infection, genistein has an anticancer effect based on the induction of apoptosis and a reduction of cell viability [162]. To be specific, genistein was shown to dose-dependently increase the levels of caspase 2 and the cleaved poly-ADP-ribose polymerases (PARP) involved in apoptosis to induce apoptosis [163]. What is of note here is that genistein activates the endoplasmic reticulum (EPR) stress as a way to exert its anticancer effect against cervical cancer [164]. EPR stress arises when abnormal proteins accumulate in the EPR and prolonged EPR stress causes apoptosis. Genistein significantly promotes the expression of GRP78, a molecular marker of EPR stress, and activates EPR stress through the strong expression of C/EBP homologous protein (CHOP), an important mediator of apoptosis that induce EPR stress, thereby ultimately leading to apoptosis induction in cervical cancer cells [165]. Genistein was also shown to increase lymphocyte proliferation as well as the release of lactate dehydrogenase (LDH) and interferon gamma (IFN-γ). Such effects on lymphocyte proliferation, cytolytic activity, and IFN-γ production were produced by genistein to inhibit tumor growth (Table S6) [166].

The ingestion of soybean products or dietary isoflavone intake were reported to show protective effects against prostate, ovarian, gastric, and overall colorectal cancer, and an association with reduced risks of colon and rectal cancer. The effects were particularly prominent in postmenopausal women [34,166,167,168,169,170,171,172]. To give an illustration, genistein inhibits prostate cancer by regulating various cell signaling systems and miRNA in prostate cancer. Genistein was shown to effectively reduce the mRNA expression of the prostate cancer marker KLK4 while showing a tendency to reduce androgens and the cell cycle-related indicators and increasing the expression of the cell cycle inhibitor p27kip1 [173]. In another study, genistein was shown to inhibit prostate cancer cell growth by inducing the expression of tumor-suppressing miR-34A to downregulate HOX transcript antisense RNA (HOTAIR). HOTAIR influences the apoptosis and cell cycle in prostate cancer cells and miR-34A regulates the expression of HOTAIR [174]. Genistein was also reported to contribute to an antitumor effect on prostate cancer by reducing methylation and increasing the genes expression levels of ADCY4 encoding adenylate cyclase 4 and NEU1 encoding lysosomal sialidase that collectively governs the cell cycle, vasculogenesis, cellular immune response, and intracellular signaling (Table S6) [175].

Soybeans contain different types of isoflavones, and among them, daidzein has been reported by various studies as a substance with an antitumor effect. In addition, daidzein is metabolized by the intestinal microflora into a substance called *S*-equol existing as an active form (hereinafter referred to equol) but not *D*-equol [5,176]. Soy isoflavones and the metabolite equol contribute to cancer cell growth suppression and prevention by inducing a fall in immunosuppressive cells [177,178,179]. With an increasing number of studies reporting on the effectiveness of bean products and soy isoflavones in cancer prevention, much interest has been focused on not only the high-level effectiveness of soy isoflavones but also their safety and synergistic effects as a health supplement. A combined treatment of daidzein and genistein was shown to effectively inhibit prostate cancer cell proliferation with a synergistic effect on apoptosis induction (Table S6) [180].

Cancer stem cells play an important role in the occurrence, progression, and recurrence of kidney cancer and the Sonic hedgehog (Shh) signaling pathway (a major regulator of cell differentiation and proliferation) maintains cancer stem cells [181]. Genistein has anticancer properties, but its mechanistic effect on kidney cancer stem cells have not yet been revealed. A recent study showed that genistein inhibits proliferation and induces apoptosis in kidney cancer stem cells. An in vitro study investigating the role of the Shh signaling pathway in the anticancer inhibitory effect of genistein showed that genistein targeted cancer stem cells in malignant tumors, and significantly reduced the number and size of kidney cancer stem cells in a dose-dependent manner. In addition, genistein had an effect on cell proliferation and apoptosis and dose-dependently decreased the expression levels of the Shh pathway-related proteins (Table S6) [182]. These results show that genistein has an inhibitory effect on renal cancer stem cells by inhibiting the Shh signaling pathway.

In addition to the inhibitory effect of isoflavones on ER breast cancer, genistein has an inhibitory effect on ER–breast cancer (Figure 4) [183,184]. Recently, phytoestrogens such as isoflavones also activate G protein-coupled receptor GPR30 (also known as GPER-1) associated with several physiopathological disorders and especially in estrogen-dependent diseases such as breast cancer [185]. Apart from inhibiting cell proliferation and inducing apoptosis, genistein is also a strong antioxidant that reduces oxidative stress, repairs DNA damage, and inhibits tumor angiogenesis and metastasis. As such, isoflavones induce normal apoptosis, regulate cell proliferation, inhibit cancer angiogenesis and metastasis, and control abnormal mutations in cells through their antioxidant activities. Research on cancer suppression is on the rise. In addition to hormone-related cancers such as breast and prostate cancers, isoflavones have cancer-suppressing effects on colon, ovarian, and lung cancer. Isoflavones also reduce resistance to anticancer drugs. These results may explain the anticancer activity of isoflavones. Although this is an area that requires further research, it is believed that there is no harm in breast cancer patients taking isoflavones other than their suppressive effect on tamoxifen, a breast cancer-treating drug [185,186,187,188,189,190,191,192,193,194,195,196]. Studies have shown that despite reducing the action of tamoxifen [197], isoflavones decrease mortality and recurrence rates as well as metastasis in breast cancer patients [198,199]. On the other hand, isoflavone intake does not adversely affect markers of breast cancer risk, including mammographic density and cell proliferation in clinical trial. Prospective epidemiologic studies in the USA and China show that post-diagnosis soy intake significantly reduces recurrence and reduces recurrence and improves survival (Table S6) [186].

### 4.13. Promotion of Osteogenesis and the Prevention of Osteoporosis

Bone plays an important role in the formation of skeletons as well as blood cells and mineral metabolism in the body. As bone ages, bone minerals decrease and the risk of fracture increases [200]. Osteoporosis is almost always associated with women between the ages of 55 and 70 [201]. Calcium levels decrease after the age of 30, and after menopause, female hormone levels decrease, leading to a reduction in bone density, and consequently the risk of fracture is highest at this time of life [202]. Bone health is determined early on in life. Approximately, 90% of bone mineral density is formed in adolescence, and the peak bone mass, which is the maximum amount of bone a person has throughout one’s life, is generally reached in the early 20s for women and the late 20s for men [203,204]. Therefore, it is important to take good care of bone health during growth, and studies have shown that soy ingredients and foods are beneficial for bone health.

**Figure 10:**
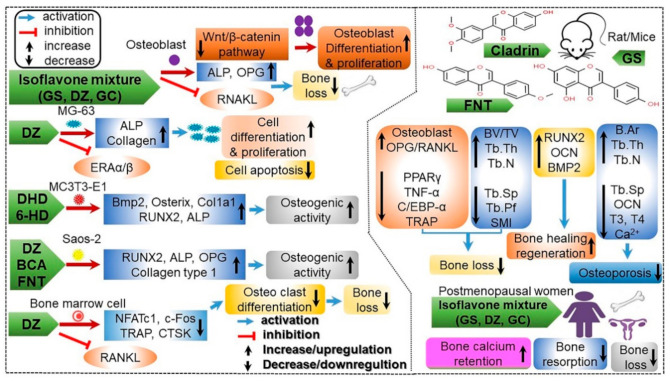
Antiosteoporotic effects of daidzein (DZ), genistein (GS), glycetin (GC), and their metabolites [6-hydroxydaidzein (6-HD), formononetin (FNT), dihydrodaidzein (DHD), biochanin A (BCA), and cladrin] through modulation of gene expression at transcriptional and translational levels in different bone cell types. Cell differentiation and proliferation, osteogenic activity, and bone health (such as bone density) is enhanced, whereas apoptosis, bone loss, and bone resorption are attenuated in experimental settings (involving MG-63, MC3T3-E1, Saos-2, and bone marrow cells and in vitro experiments in rats/mice). In osteoblast cells, GS, DZ and GC upregulate the expression of genes associated with alkaline phosphatase (ALP) and osteoprotegerin (OPG), and downregulate Wnt/β-catenin pathway, whereas they inhibit receptor of activator of nuclear factor kappa B ligand (RANKL). DZ induces increased ALP and collagen synthesis, and inhibits estrogen receptor A alpha/beta (ERAα/β) in MG-63 cells. DZ also decreases the expression of genes associated with nuclear factor of activated T-cells cytoplasmic 1 (NFATc1), cellular oncogene fos (c-Fos), tartrate-resistant acid phosphatase (TRAP) and cathepsin K (CTSK), as well as inhibits RANKL in bone marrow cells. DHD and 6-HD upregulates the expression of genes associated with the bone morphogenetic protein 2 (BMP2), transcription factor Sp7 (Osterix), alpha-1 type I collagen (COL1a1), runt-related transcription factor 2 (RUNX2), and ALP in MC3T3-E1 cells. DZ, BCA, and FNT increase the expression of genes associated with RUNX2, ALP, OPG, and COL1 in Saos-2 cells. As shown in the right panel, cladrin, GS, and FNT improve bone health by regulating a wide range of genes in rat and mice. In addition, mixed intake of GS, DZ, and GC increases bone calcium retention and decreases bone resorption and bone loss in postmenopausal women [78]. ↑, enhancing; ↓, lowering; →, activation; ┤, inhibition. Figure adapted from Kim et al. [7].

Many studies have shown that isoflavones reduce bone resorption and stimulate production; however, some studies report that there is no particular difference. Recently, promising results have shown that isoflavones have a positive effect on bone turnover. As bone health is consistently deteriorating as the population ages, a strategy to reduce fragility and fracture risk due to aging is required [205]. Soy isoflavones and hop prenylflavanones have been traditionally used as a natural estrogen replacement therapies [205], but their potential effects are still debatable. To investigate the effects of soy flavanone and hop prenylflavanone (Soy-Hop) intake on bone loss and metabolic dysfunction in the estrogen-deficient state, a preclinical study was conducted on ovariectomized mice that were fed a high-fat diet and Soy-Hop (0, 30, 100, and 300 mg/kg) at different doses for 8 weeks, and the metabolic and bone-related parameters were analyzed. The results showed that Soy-Hop intake significantly inhibited estrogen deficiency-induced increases in body weight, fat mass, leptin, LDL-cholesterol, total cholesterol, triglycerides, and glucose and insulin circulation. Here, Soy-Hop stimulated leptin and insulin sensitivity, improving the metabolic dysfunction associated with estrogen deficiency. In addition, by inhibiting the bone turnover of the femur, Soy-Hop alleviated the bone loss associated with estrogen deficiency [205]. This suggests that the combination of soy isoflavone and hop prenylflavanone can improve the quality of life after menopause by reducing the severity of osteoporosis and improving metabolism. In addition, genistein, a type of isoflavone, uses the Wnt3a/beta–catenin signaling pathway to promote osteoprotegerin protein synthesis in the bone-forming osteoblast, and plays a critical role in facilitating the differentiation and proliferation of osteoblasts by inhibiting the conversion of a receptor activator of the nuclear factor-κB ligand (RANKL) into the bone-destroying osteoclast in bone marrow stem cells (Figure 10; Table S7) [78,206].

Isoflavones containing genistein, daidzein and equol as a yellow pigment, and β-carotene as a red pigment, reportedly play an effective role in maintaining bone health (Figure 10) [78]. Isoflavones enhance the quality of the neck bone and femur by increasing the contents of the collagen fiber and glycosaminoglycan, a morphological parameter in bone tissue [207,208]. Isoflavones improve bone growth and quality in growing female mice [209]. To investigate the effects of isoflavones on the tibia length and bone density of growing female mice, 3-week-old female mice were divided into four groups [control group (distilled water), a low dose of isoflavone group (10 mg/kg body weight/day), a high dose of isoflavone group (50 mg/kg body weight/day), and 17β-estradiol group (10 µg subcutaneous injections)]. After 8 weeks, bone-related parameters [alkaline phosphatase (ALP), osteocalcin, bone length, failure load, hardness, bone density, and structural parameters] were analyzed. The results indicated that the administration of low doses of isoflavone in growing female mice promoted bone growth, as the length of their tibia and femurs were longer when compared with the control and the high dose isoflavone groups. High dose of isoflavone improved bone marrow volume, thickness, number per unit, and bone density. In addition, isoflavone intake did not induce vaginal opening, a marker for puberty onset (Table S7) [207]. These results suggest that isoflavone intake in childhood has a positive effect on bone growth by increasing peak bone mass without serious concerns about early puberty onset and decreasing the risk of fracture in children.

Combining isoflavones and β-carotene have been shown to induce osteoblast differentiation via ALP activation (Figure 10), which is useful in maintaining the balance of bone turnover. For bone health-related effects for each isoflavone constituent, genistein showed a higher bone density in the lumbar spine and stronger effects on osteogenesis in the spine than others, while daidzein intake had the best effects on bone mineral deposition [210]. On the other hand, isoflavones are known to increase osteocalcin levels to reduce bone density and promote osteogenesis (Table S7) [211].

Isoflavone-enriched soymilk powder prevented bone mineral density reduction and promoted bone formation [209,211]. To determine the effects of isoflavone-enriched soymilk on bone metabolism in postmenopausal women, isoflavone-enriched soymilk (10% and 20%) was fed to ovariectomized mice for 8 weeks, and bone-related parameters (bone density, ALP, and osteocalcin) were analyzed. The results showed that the bone density of the ovariectomized mice fed isoflavone-enriched soymilk powder was significantly higher than that of the control ovariectomized mice. In addition, osteocalcin levels were significantly decreased in the ovariectomized mice, but isoflavone-enriched soymilk intake increased the osteocalcin levels in a dose-dependent manner (Table S7) [211]. This suggests that soymilk powder prevented the decrease in bone density and promoted bone formation in menopausal mice, confirming its potential as a hormone replacement therapy for bone health.

The aging of bones differs by gender and individuals. To maintain healthy bones, essential nutrients should be supplied to the body through foods rich in the required components, and regular exercise for strength. Furthermore, the consumption of soy foods that strengthen bones from childhood and prevent osteoporosis after menopause should also be considered.

### 4.14. Improvement of Blood Glucose Homeostasis

The number of people diagnosed with diabetes is rapidly increasing worldwide. The increase in the prevalence of diabetes is alarming as it can cause serious health complications, affecting the quality of life and even leading to death. Diabetes is not an acute disease that can be cured, but rather a chronic disease that requires effective continuous management. For diabetics, exercise burns calories, improves the effectiveness of diet, directly lowers blood sugar, prevents long-term complications, and relieves stress. Diet in diabetics should not be about reducing or limiting certain foods but about planning and practicing healthy eating habits. Regular eating habits as well as reducing the intake of simple sugars, such as sugar or honey that promotes increased blood sugar levels, as well as appropriate dietary fiber intake as it lowers blood sugar levels and blood lipid concentrations. In addition, it is important to maintain good nutrition by ingesting a variety of foods [212,213,214,215,216].

An animal study reported that fasting blood glucose levels decreased in diabetic animals that ingested genistein. In addition, a study on postmenopausal women compared fasting blood glucose levels and blood insulin concentrations between groups that either received hormone therapy (estrogen, 0.625 mg) or consumed isoflavones (100 mg/day) for 6 months. Isoflavones were observed to be as effective as hormone therapy in improving blood sugar levels and reducing insulin levels in the blood. In the group that received hormone therapy, fasting blood glucose levels and blood insulin concentration decreased by 17% and 44%, respectively, whereas in the group that received isoflavones, fasting blood glucose levels and blood insulin concentration decreased by 15% and 33%, respectively (Table S7) [217,218].

Soybean proteins suppress the biosynthesis of fatty acids and triglycerides in the liver and inhibit triglyceride accumulation to reduce lipid accumulation in the tissues. Through such cross-protection effects, soy isoflavones allow for overall blood glucose control based as they reduce body fat accumulation as well as body weight. An increase in body fat leads to increased insulin resistance but soy isoflavones reduce body fat and thus decrease insulin secretion and prevent insulin resistance and pancreatic cell dysfunction by improving insulin sensitivity [219,220].

Genistein was found to have protective effect on the pancreas from autoimmunity, which lowers type I diabetes incidence and delays the onset of the disease [221]. Equol, a daidzein metabolite produced by the intestinal microflora, is a type of isoflavones that show anti-diabetic and anti-hypertensive effects as it increases glucose utilization in muscle cells. Equol inhibits gene expression related to the enzymes of glucose metabolism, thereby suppressing the levels of serum glucose, cholesterol, triglycerides, and lipid peroxides, as well as a rise in triglyceride level. Equol also inhibits a rise in fasting blood glucose and promotes gene expression for gluconeogenesis and lipogenesis (Figure 6; Table S7) [222,223].

Type 2 diabetes is associated with an increased risk of bone fractures [224] while the effects of soybean on the bone turnover marker in type 2diabetes male patients remain unclear. According to a recent report, an intake of soybean proteins containing isoflavones reduced the bone resorption marker βCTX (collagen type 1 cross-linked β C-telopeptide) which prevents the removal of minerals from the bones. A significant linear correlation was also shown between βCTX reduction and glycated hemoglobin (HbA1) reduction. These results showed that the intake of soybean proteins containing isoflavones uses a mechanism to reduce βCTX related to blood glucose control and insulin tolerance enhancement by altering insulin resistance [225,226]. This confirmed that by altering insulin resistance in males with type 2 diabetes, the consumption of soy protein containing isoflavones for 3 months reduced βCTX, which is associated with the improvement of blood sugar control and insulin resistance [225]. The intake of soy isoflavones, furthermore, improves the blood glucose level and lowers the risk factors in type 2 diabetes patients. The risk of diabetes fell by 13% with daidzein intake and by 9% with genistein intake [227]. The intake of soybean proteins plus isoflavones was also shown to improve the reactive hyperemia index and blood glucose control by reducing the glycated hemoglobin A1c (HbA1c) [225]. In addition, isoflavones exhibit preventive effects on hyperglycemia by inhibiting the activity of α-glucosidase, suppressing glucose absorption, and reducing the oxidation and inflammatory damage markers (LPO, CRP, and IL-6) (Figure 6; Table S7) [228].

Among soy isoflavones, a mixture of daidzein and glycitin was shown to be effective in preventing obesity and diabetes (Figure 6). The isoflavones mixture lowered the weights of epididymis and visceral adipose tissue to a level equal with those of a healthy-diet case while increasing the level of the beneficial HDL-cholesterol. The mixture also induced a low level for the fasting blood glucose, serum glycated hemoglobin, and serum insulin. Moreover, the mixture was shown to maintain the high content of liver GSH and to reduce the level of plasma 8-hydroxy-2-deoxyguanosine (8-OHdG) (Table S7) [229]. Such results indicate that daidzein and glycitin inhibit adipose tissue formation due to the high-fat diet as well as the advancement of diabetes based on the regulation of oxidative stress, a side effect of harmful oxygen free radicals that induce various diseases.

Equol, a metabolite of daidzein, has been proposed as a chemoprotective agent for type 2 diabetes [230], but its underlying mechanisms have not yet been elucidated. Recently, in vitro and preclinical studies were conducted to determine the mechanisms by which equol stimulates the β-cell response (Figure 6). The administration of equol for 7 days increased pancreatic β-cell function in experimental mice by 27% and increased glucose-stimulated insulin secretion in the islets of mice by 41%. By inhibiting protein kinase A, equol showed effects on cell growth, insulin secretion, and cAMP-response element (CRE)-mediated transcription in INS cells (insulin-secreting β-cell model), and the effect of equol was superior to that of daidzein (Table S7) [230]. This suggests that equol in legumes improves pancreatic β-cell function and protects type 2 diabetics against hyperglycemia.

Soy isoflavones prevent hyperglycemia by lowering blood sugar levels. Goto-Kakizaki (GK) mice with a genetic background that makes them susceptible to type 2 diabetes were fed isoflavone either once or long-term [228]. A single administration of isoflavone reduced blood sugar levels, and long-term administration inhibited α-glucosidase in the small intestine of mice. Isoflavone inhibited glucose absorption in a dose-dependent manner and reduced GSP levels, the glycemic status index, and oxidative and inflammatory damage indices (LPO, CRP, and IL-6) [228]. This confirmed that isoflavone is effective in preventing hyperglycemia through the inhibition of carbohydrate digestion and glucose uptake in the small intestine of GK mice with type 2 diabetes, proving that isoflavone is a potential candidate as a therapeutic agent for type 2 diabetes (Table S7).

It is important to improve lifestyle habits to prevent diabetes. Upon improvement of lifestyle habits via moderate physical activity for 150 min per week, the incidence of diabetes decreased by 58%, and it decreased by 71% in adults over 60 years of age. Lifestyle improvement starts with a balanced diet. For people with an irregular lifestyle, a balanced diet as well as the consumption of legumes, which are good for blood sugar management and diabetes prevention, is required.

### 4.15. Reduced Inflammation and Skin Cell Protection in Wound Tissue

A study showed that it takes only 3 s to make a first impression, and skin is one of the major factors that influence first impressions [231]. Apart from that, it plays a variety of physiological roles, including controlling the body temperature, maintaining moisture, and recognizing senses, as well as acting as a primary barrier to protect our body from the external environment [232]. Appearance changes with age, and as age increases, the wound healing ability of the skin is reduced, and the survival rate of skin flaps decreases [233]. Various skin problems, such as redness and psoriasis, skin damage due to aging, and decreased skin elasticity can change how a person is perceived by others; therefore, many people, regardless of age or gender, are becoming more interested in skincare [234]. Appearance is bound to change over the years, but diet, one of the secrets for elastic and shiny skin, helps maintain the acidity of the skin and keeps the skin healthy. This sectionintroduces the latest studies on the effects of legumes on skin health.

The metabolism and absorption of isoflavones have largely been characterized as mentioned above; however, studies on their role in the prevention of skin damage caused by ultraviolet (UV) light are relatively rare, and thus the mechanisms are unclear. The protective effect of isoflavones for the skin has recently begun to be identified. An excess exposure to UV light causes various skin problems, and UV exposure in particular, induces skin inflammation by increasing skin DNA damage and oxidative stress. In human skin fibroblasts, daidzein and genistein, are effective for skin protection against ultraviolet B (UVB)-induced damage and help to reduce skin inflammation. While UVB increases the expressions of inducible nitric oxide synthase (iNOS) and cyclooxygenase-2 (COX-2), an intake of fermented soybeans containing isoflavones inhibits the expressions of iNOS and COX-2 [235]. Both the isoflavone glycoside and aglycone showed anti-inflammatory effects and effectively prevented UV-induced DNA damage (Table S8) [236].

Genistein produces antioxidant enzymes to protect the skin fibroblasts from aging and prevent intracellular oxidative stress [237]. The production of macrophage cytokines was inhibited in vitro and the level of inflammatory response in the wound tissue was reduced in vivo to aid in re-epithelialization. Soybean extracts have anti-aging effects on the skin as they regulate matrix metalloproteinase-1 (MMP-1) related to collagen reduction, tissue inhibitor of metalloproteinase-1 (TIMP-1) related to MMP-1 activity inhibition, and HAS2 and CRABP2 genes for retinoid regulation (Table S8) [238].

Glycitin, a type of soybean isoflavone, is a functional substance reported to have antioxidant, anti-allergic, and anti-osteoporosis effects [239]. It has also been reported to engage in skin cell protection against aging and wrinkle formation. Glycitin in fibroblasts was shown to induce the synthesis of type I and type II collagen and fibronectin, while reducing the activities of the typical skin aging indicators, elastase, matrix metalloproteinase, and β-galactosidase. Based on these results, glycitin was shown to ultimately protect skin cells against aging and wrinkle formation by stimulating the secretion of transforming growth factor-beta (TGF-β) which in turn induces fibroblast proliferation (Table S8) [239].

Following ingestion, isoflavones are converted to metabolites in the human gastrointestinal tract, and if one were to understand the physiological effects of soy isoflavones in the body, the individual bioactivity of their metabolites should also be assessed [5,78]. Among them, daidzein leads to a metabolite 6,7,4-trihydroxyisoflavone (6,7,4-THIF) that exhibits anti-photoaging effects. The 6,7,4-THIF treatment was shown to dose-dependently reduce the expression of MMP-1 whereas daidzein did not significantly reduce MMP-1 expression. 6,7,4-THIF also inhibited UV-induced the mitogen-activated protein kinase (MAPK) signaling pathway, and dose-dependently inhibited protein kinase C alpha (PKCα) activity by directly binding to the PKC, an upstream regulator of MAPKKs (Table S8) [240]. These results indicated that 6,7,4-THIF, as a daidzein metabolite, had PKCα as its direct molecular target in UV-induced MMP-1 expression, and that it had anti-photoaging effects based on the inhibition of MMP-1 that plays a crucial role in wrinkle formation.

In both East Asian and Western countries, people have become increasing interested in maintaining a healthy skin. This has led to ongoing studies focused on the anti-wrinkle effects of a mixture of substances that have been reported to be associated with the key factors of skin aging such as inflammation, collagen synthesis, oxidative stress, and UV stress (Table S8) [241,242]. For instance, studies have reported on the cases where an intake of the mixture containing soy isoflavones, lycopene, vitamin C, vitamin E, and omega 3 fatty acid produced positive effects on facial wrinkle improvement [243].

Isoflavone has been considered an antiaging product due to its antioxidant properties [237]. A study using pigs was conducted to confirm the effect of nanoemulsions, prepared by hydrolyzing or nonhydrolyzing soybean extract containing isoflavones (genistin and genistein), on skin protection from UV rays. The results showed that the nanoemulsion with isoflavone-containing hydrolyzed soybean extract improved skin permeability and the nonhydrolyzed soybean extract containing isoflavone improved skin retention. The antioxidant effect was higher in nanoemulsions containing isoflavone-containing soybean extract than in those of pure isoflavones (Table S8) [244]. This suggests that the nanoemulsion containing isoflavone-rich soybean extract can be used as a topical formulation to protect the skin from UVA/UVB oxidative damage.

Isoflavone extracts are effective in treating psoriasis and other inflammatory skin diseases. Psoriasis is a common inflammatory disease that affects 1–3% of the world’s population, and medical expenses for its treatment are increasing every year [245]. A preclinical study was conducted with experimental mice to confirm the therapeutic effects of the isoflavone extract on the prevention of psoriasis and the alleviation of inflammatory effects in epidermal keratinocytes in psoriatic skin. The results indicated that isoflavone extracts reduced skin inflammation and psoriasis caused by imiquimod (which is an immune response activator but induces and exacerbates psoriasis). Isoflavone reduced the activity of MAPK, NF-κB, and JAK-STAT by IL-22, IL-17A, and TNF-α in epidermal keratinocytes; TNF-α and NF-κB levels were high in the skin lesions of patients with psoriasis, and isoflavone extracts play a large role in their inflammatory responses (Figure 8) [78]. Isoflavone extracts significantly reduced transepidermal water loss (TEWL), red spots, blood flow rate, and ear thickness, increased water moisture in the surface, and reduced epidermal hyperplasia and inflammatory cell infiltration in the experimental mice (Table S8). This shows the potential of isoflavone extract in treating psoriasis and other inflammatory skin diseases [245].

Genistein improves the viability of skin flaps (unlike skin transplantation, a tissue is supplied with blood by itself, and because the skin is moved while securing blood circulation without dividing part of the graft, the surgery is difficult to perform and there could be severe sequelae if it fails) [244]. A study with experimental mice was conducted to investigate the viability of genistein, a natural selective estrogen receptor modulator (SERM) of skin flaps, for its antioxidant properties on endothelial cells, and whether necrosis could be prevented after blood flow damage. The results showed that the flaps survival rate between groups significantly increased when treated with genistein, and genistein had a protective effect against skin ischemia. Genistein did not induce cytotoxicity, and promoted the migration of endothelial cells by 15%. Mechanistically, genistein treatments for skin flaps significantly improved wound healing via the upregulation of Bcl-2 (promoting cell survival) and SOD (protecting cells from toxicity) [246]. This proved that the genistein treatment for 3 days before the skin flap surgery led to a protective effect on the ischemia of the skin tissue and suggests that it could potentially be used as a therapeutic agent to improve skin flap viability (Table S8).

Genistein and 17β-estradiol (an estrogen steroid hormone) have been reported to have antioxidative effects on human skin cells by regulating the level of peroxide. Based on previous studies that have shown estrogen to improve skin health, the anti-aging mechanisms of genistein, a phytoestrogen, and 17β-estradiol were studied to confirm the effects of genistein on skin. A preclinical experiment was conducted using fibroblasts and keratinocytes, which are cells that play an important role in human skin regeneration. Indices such as NO, mitochondrial membrane potential, ROS, and GSH were anayzed to confirm any antioxidative effects. After hydrogen peroxide treatment, the amount of nitric oxide, a substance related to photoaging of the skin, increased by 55% and 61% in fibroblasts and keratinocytes, respectively, and ROSthat promote cellular aging, increased by 25%. Injection of genistein and 17β-estradiol led to a significant decrease in these indices by more than half or to a level similar to that of the control, depending on the dose. Further, the mitochondrial membrane potential was measured and the ratio of normal cells to apoptotic cells was compared. Similar to the previous results, a dose-dependent result was found. The ratio of glutathione, which had decreased, increased back to the level observed in the control group (Table S8) [247]. Based on these results, genistein and 17β-estradiol are thought to help prevent aging by reducing oxidative stress and removing peroxides that cause aging of skin cells.

Recently, it has been reported that the soy isoflavones genistein and daidzein can help prevent skin aging in male mice. Besides, it was confirmed that soy isoflavones are effective in preventing skin aging in menopausal humans and rats; however, there has been no research related the effect of isoflavones on male skin. In a study on male mice, a mixture of genistein and daidzein was added to the diet starting from prenatal to sexual development, following which, skin thickness, number of fibroblasts, and collagen fiber diameter were measured to observe the effect of isoflavones. There was a significant increase in skin thickness, diameter of collagen fibers, and the amount of elastic fibers in the group of male mice that ingested isoflavones. This is likely because genistein affects insulin growth factor receptors, which cause keratinocyte movement and acceleration of re-epithelialization through an estrogen-independent pathway. This improves skin barrier function, which results in an increase in skin thickness. Due to the antioxidative effect of isoflavones, catalase (CAT) activity in the skin was not required, resulting in a decrease in the level of CAT in the skin homogenate. Lipid peroxide formation was inhibited when the genistein-daidzein mixture was administered at a high dose (Table S8) [248]. These results confirm that the mixture acts as an effective antioxidant not only in women, but also in men. It also positively affects the antioxidant enzyme system, thereby improving skin health by relieving oxidative stress in the skin cells.

It is believed that genistein’s anti-inflammatory effect can be used to develop treatments for psoriasis, a skin disorder. To understand the mechanism underlying the anti-psoriatic effects of genistein, a preclinical experiment was conducted to verify the effect of genistein on imiquimod-induced psoriasis lesions (HaCaT cells). Both in vivo and in vitro, genistein inhibited the expression of mRNAs that upregulate TNF-α-induced inflammatory cytokines and chemokines such as IL-1β, IL-6, VEGFA, and CCL2. Thus, TNF-α-induced proliferation of HaCaT cells, and the resulting inflammatory response was inhibited. This inhibition led to the suppression of STAT3 and NF-κB phosphorylation, which play important roles in the pathogenesis of psoriasis, and blocked STAT3 and NF-κB signaling, thereby reducing the proliferation of psoriasis-induced HaCaT cells (Table S8) [249]. These results suggest that genistein’s ability to regulate the expression of inflammatory factors can be used to design treatment strategies for chronic recurrent skin disorders such as psoriasis. As mentioned above, many studies have shown that genistein and daidzein repair skin cells damaged by UV rays (Table S8). However, due to inadequate information in this regard, it is important to conduct studies to develop methods to effectively deliver isoflavones to the skin. For healthy skin, it is necessary to get adequate amount of sleep, control stress, and promote metabolism. In addition, the steady consumption of soy foods, which have excellent antioxidant effects, can help maintain skin health by reducing skin inflammation and reducing the signs of skin aging.

### 4.16. Enhanced Quality of Life for Postmenopausal Women Based on Improved Physical and Emotional Symptoms

Women tend to have more health problems than men, as their bodies change significantly throughout their life owing to physiological processes such as menstruation, pregnancy, childbirth, and menopause. In particular, the period when menstruation completely disappears, called perimenopause or menopause is an important stage of development women, which begins around the late 40 s. Most women experience menopausal syndrome, which can include hot flushes, cold sweats, lethargy, depression, memory problems, and sleeping problems. In addition, as the secretion of the female hormone estrogen decreases, the risk of developing diseases, such as osteoporosis, dementia, and cardiovascular diseases increases [101,133,250,251,252]. Therefore, women require proper exercise and a regular diet to lead a healthy life.

**Figure 11:**
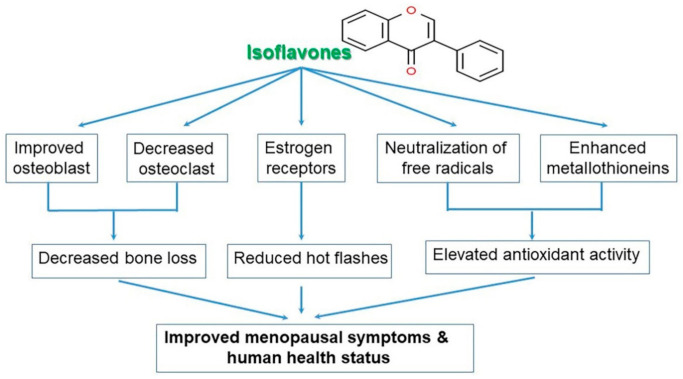
Improved menopausal symptoms throughisoflavones. Isoflavones improves osteoblast, estrogen receptor beta-binding capacity, reactive oxygen species-neutralization ability, including enzymatic antioxidant systems (e.g., metallothioneins), and they decrease osteoclast frequently. Therefore, isoflavones prevent bone loss and hot flashes, and enhance antioxidant activity, thereby ameliorating the menopausal symptoms and improving human health status [250,253].

The female hormone estrogen acts to protect the blood vessels by inhibiting the LDL cholesterol that may harm the blood vessels and by increasing the level of beneficial HDL cholesterol to prevent waste material from accumulating in arterial vessels. Nonetheless, as estrogen levels decrease following menopause, such vasoprotective functions rapidly decrease, which increases the risk of vascular diseases including hypertension and hyperlipidemia in menopausal women (Figure 11) [253]. Soybeans have been shown by numerous studies to be a food that brings multiple benefits to menopausal women as they not only relieve the menopausal syndrome but can also lower blood pressure and improve blood vessel function [254]. The intake of soybean proteins and isoflavones were shown to significantly reduce the systolic pressure (4.25%) and to decrease the well-known arteriosclerosis risk factors such as E-selectin and soluble intercellular adhesion molecule (sICAM)-1 (Table S9) [255].

Isoflavones were also shown to help improve the physical and emotional symptoms in premenopausal and postmenopausal women [12], such as facial flushes, heart symptoms, sleep disorders, depression, hypersensitivity, anxiety, fatigue, sexual and bladder dysfunction, vaginal dryness, and rheumatic problems [256]. In premenopausal women, physical symptoms improved by 27.7%, emotional symptoms by 26.3%, and urinary-reproductive symptoms by 1.2%. In postmenopausal women, physical symptoms improved by 14.5% and emotional symptoms by 26.8% [257], while urinary-reproductive symptoms and health-related quality of life also improved (Table S9) [254,257,258,259,260]. These results indicate that soy isoflavones could improve the physical and emotional symptoms of menopause in both premenopausal and postmenopausal women, to help them lead healthier lives. As mentioned above, studies have reported on the positive effects of soy isoflavones on improving cognitive functions but whether such findings apply equally to postmenopausal women is still debated. A recent study has reported that isoflavone intake had a positive influence on improving the cognitive function and visual memory in postmenopausal women [261].

Female sexual dysfunction is a common endocrine disorder that impairs the quality of life of many women. This can manifest as emotional problems, concerns about the sexual interest of women, or vaginal pain, and has a direct or indirect effect on the quality of life such as inability to have children, which can further lead to depression or suicide [262,263]. The worldwide prevalence of female sexual dysfunction is about 70%, and can affect women of all ages [264]. A study analyzed serum hormone levels, vaginal histological changes, and enzyme-linked immunosorbent assays in mice to confirm the effect of isoflavone on female sexual function and the underlying mechanisms. The results indicated that vaginal blood flow was significantly lower in the adult mice of the control group than in the mice that were fed isoflavone, in response to pelvic nerve stimulation in a dose-dependent manner [265]. The estrogen levels and the number of mature follicles significantly increased in isoflavone fed mice, suggesting that isoflavones promote the maturation of ovarian follicles [265]. In addition, isoflavone dose-dependently improved sexual dysfunction by activating the endothelial nitric oxide synthase (eNOS) signaling pathway via the estrogen receptor [265]. This shows that isoflavones in soybeans could improve sexual function in middle-aged female mice and confirms its potential for treating sexual dysfunction.

A systemic review and meta-analysis of randomized clinical trials (RCT) evaluated the efficacy of isoflavones and equols in alleviating menopausal symptoms, particularly vasomotor symptoms, in postmenopausal women that produce or do not produce equols [266]. In the study, five out of twelve RCT studies were systematically reviewed and meta-analyzed, and three of these studies reported the benefits of equol. In particular, the meta-analysis results showed that 10 mg of equol reduced hot flashes by 23% when compared to the control group. This shows that in postmenopausal women who can produce equol in the intestine, that 10 mg of equol per day through a diet rich in isoflavone could reduce hot flashes, thereby alleviating some menopausal symptoms (Table S9) [266]. Taken together, these results show that equol, a metabolite of soy isoflavones, can reduce hot flashes in postmenopausal women.

Isoflavones improve sleep disorders in postmenopausal women [267]. Menopausal women were divided into two groups [natural isoflavone complex (isoflavone + Lactobacillus + agnus-castus (a medicinal plant of the Verbenaceae family known to be effective on menstrual irregularities and premenstrual syndrome) + magnolia) and isoflavone alone], and Kupperman Index (evaluating a total of 11 items including hot flashes, insomnia, depression, and vaginal dryness), Pittsburgh Sleep Quality Index, endometrial thickness, breast density, and liver function were analyzed. The two methods used for isoflavone intake for 12 months resulted in no differences in breast density, endometrial thickness, or liver function, confirming its safety. In addition, hot flashes and sleep quality improved, and in particular, natural isoflavone complexes had a better effect at improving sleep disorders (Table S9) [258]. This suggests that the natural isoflavone complex could potentially be used as a treatment for sleep quality and vasomotor symptoms.

Soybeans are healthy and are especially beneficial to women’s health. As previously discussed, soybeans not only help reduce menopausal symptoms and sexual dysfunction but also lower the chances of endometrial cancer, breast cancer, and ovarian cancer caused by the effects of female hormones; therefore, soybean consumption is highly recommended for women (Figure 11). Additionally, menopause is not a disease, but a process of physical change, and since it leads to psychological changes. To come through menopause in a health manner, proper exercise and a regular diet are necessary. Consuming soybeans that help alleviate menopausal symptoms may also be beneficial.

### 4.17. Safety of Isoflavones in Reproductive Development

As interest in height has increased, and children are reaching puberty faster, more parents are concerned about the sexual development of their children. The causes of precocious puberty are childhood obesity, hormonal imbalance, stress, and environmental hormones [123,268]. In the past few years, misinformation has been perceived as true, and people were reluctant to feed healthy soy foods to their children. This is because certain foods, especially legumes, contain phytoestrogens similar to female hormones, but it is not clear whether these ingredients affect puberty, and if precocious puberty could be prevented by diet [269,270,271].

Previous studies have reported that the consumption of soybeans early in life may have a negative influence on reproductive development (mammary gland development, first menstrual period, endometrium, testis, etc.) [272,273,274,275]. However, the findings of recent studies show that soybean ingestion during infancy had absolutely no influence on precocious or early puberty. No intergroup difference or difference in association with puberty was found due to the daily intake of soybeans, micronutrients, energy, carbohydrates, lipids, and proteins [276]. Continuous soybean food intake for a year was shown to improve the nutritional status of the children [277]. In other words, soybean proteins acted as a selective estrogen receptor modulator that had close to zero influence during reproductive development and soybean protein intake during infancy was confirmed to be safe with regard to reproductive development [278]. In addition, genistein intake during pregnancy showed a protective effect for the fetal reproductive organs against harmful bisphenol A (Table S10) [187,279].

Dietary habits in childhood are often maintained in adulthood [193]. For children to acquire and maintain healthy dietary patterns throughout their lives, it is recommended that they experience a diversity of foods early in life [280]. A diet containing soybeans or soybean products during the first few years of life is thus likely to contribute greatly to the healthy lives of those children. The safety of soybeans in baby foods has been proven as infants fed with soybean-containing baby foods showed similar development those the infants fed with breast milk and milk-based baby foods in terms of growth, bone health, and metabolism, as well as reproductive, immune, and neurological development [281,282]. Furthermore, a study has shown that the ingestion of soybeans early in life leads to the effective prevention of conditions including breast and prostate cancer (Table S10) [283,284]. As such, soybeans help with growth and development while exhibiting excellent preventive effects against various adult diseases and cancer. Moreover, soybeans have protective effects against the negative impacts of endocrine disruptors so that the ingestion of beans and bean products during infancy is being emphasized for the healthy lives of children (Table S10).

Soybeans contain essential amino acids that are important for growth and development and are an important source of protein without excessive calories. Soybean and soybean processed foods are not only excellent in terms of nutrition but also rich in functional ingredients; therefore, their consumption during childhood could play an important role in preventing childhood obesity and malnutrition and reducing the risk of disease. A meta-analysis study found that infants who ate soy infant food showed similar growth and development for bone health, metabolism, reproduction, immunity, and neurodevelopment as those that consumed breast milk and formula, proving its safety.

### 4.18. Other Human Beneficial Effects of Isoflavones

#### 4.18.1. Improvement of Arteriosclerosis and Lipid Metabolism

Genistein exerts a protective effect against leptin-induced atherosclerosis [285]. Genistein was shown to inhibit the proliferation and migration of leptin-stimulated vascular smooth muscle cells by 10% and reduce the expression of leptin-induced cell cycle regulatory protein cyclin D1. In addition, although leptin caused the proliferation of new vascular smooth muscle cells in the carotid artery of mice with vascular injuries, genistein was shown to inhibit their proliferation [100,107]. In another study, an intake of a soybean fermented food containing isoflavones was shown to reduce total cholesterol by 13.8%, non-HDL cholesterol [LDL + intermediate-density lipoprotein (IDL) + very low-density lipoprotein (VLDL cholesterol)] by 14.7%, and LDL cholesterol by 24.2%. When consumed, the soybean fermented food containing isoflavones prevented the reduction of HDL cholesterol, and notably, equol that was produced as a metabolite of isoflavones led to the reduction of LDL cholesterol [286]. Such combined antioxidant and anti-inflammatory activities of isoflavones were shown to improve lipid metabolism (Table S10).

#### 4.18.2. Antioxidant Activity

Phytoestrogens have been reported as metabolites that can induce biological reactions [287]. They generally bind to an estrogen receptor (ER) under identical conditions as estrogens to play crucial roles by mimetic or intrinsic estrogen activities [14]. Soy isoflavones and their metabolites exhibit better or similar antioxidant activity to prevent oxidative stress and lower the rate of oxidation [287,288,289], as compared to flavonoids from hops and subterranean clover [290,291,292]. Although hops, beer, and subterranean (*Trifolium* *subterraneum*) and red (*Trifolium pratense*) clover exhibit a broad spectrum of biological activities based on flavonoids, including isoflavones, there is little information in human health benefits exception for hops. Present sources of isoflavones for medical/nutraceutical food development are red clover and soybean (*Glycine max* L.) (Table S10) [291].

Notably, genistein among isoflavones activates the gene expression related to antioxidant proteins [293]. In healthy mice, isoflavones reduced the content of 8-hydroxydeoxyguanosine which is a marker of somatic cell oxidation and advancement of cancer, and promoted the expression of superoxide dismutase that prevents oxidative damage (Figure 7). Such protein activities led to a reduced malondialdehyde (MDA) content, which is a product of the decomposition of lipid peroxides (Table S10) [294].

Since estrogen improves blood sugar levels and has antioxidative effects, in a study, glucose tolerance increased and antioxidant enzymes (SOD and GPX) decreased in ovariectomized mice (Figure 6; Figure 7). When these mice were administered high concentrations (150 mg/kg) of isoflavones, the levels of antioxidant enzymes increased, and glucose tolerance decreased [83,295,296]. These effects were greater in mice fed with a high-fat diet after ovariectomy. In addition, in a study on the antioxidant activities of isoflavones against stress factors after exercise, it was observed that the concentration of thiobarbituric acid-reactive substances, a lipid peroxidation factor, was decreased, and the activities of antioxidant enzymes, such as superoxide dismutase and catalase, were increased (Table S10) [62,289,297].

Genistein was shown to significantly increase the expression of the proteins involved in apoptosis (caspase-3, caspase-9, and caspase-3 target protein PARP) while reducing the expression of Bcl-2. Genistein also activated the proteins (Ask1 and JNK) that cause DNA damage, reduce the expression of an antioxidant (thioredoxin-1), and induce apoptosis in a concentration-dependent manner, in the process of DNA fragmentation and caspase-3 activation, the two typical features of apoptosis. Based on such results, genistein exerted anticancer effects through apoptosis induction in cancer cells and the inhibition of tumorigenic cells, as it downregulated the enzyme thioredoxin-1 with an antioxidant effect [159]. In addition, genistein was found to have an anti-proliferation effect on breast cancer cells (MCF-7). The apoptosis induction by genistein, in particular, was shown to entail the activation of both the intrinsic and extrinsic apoptosis pathways in relation to the partial affinity to ERα and ERβ (Table S10) [153].

Equol is an isoflavone and a metabolite of daidzein produced by the intestinal microflora used to prevent and treat various diseases [176]. The reports on equol’s antioxidant effects have recently increased. Lipopolysaccharides induce oxidative stress by increasing MDA and decreasing GSH [298]. In chicken HD11 macrophages, equol was shown to reduce the lipopolysaccharide-induced oxidative stress and enhance total SOD activity. Equol also increased the transcription of TNFα and IL-1 β and improved the inflammatory indices (TNFα, IL-1β, IL-2, and IFNβ) (Figure 6; Table S10) [298]. Thus, equol could improve the inflammatory response of macrophages by increasing the gene expression related to antioxidant activity and cytokine production to inhibit oxidative stress.

#### 4.18.3. Antiviral Effect

Among soy isoflavones, the research on genistein has been the most extensive, especially its antiviral properties [5]. In DF-1 cells, a target of avian leucosis virus subgroup J (ALV-J), genistein inhibited the gene expression in the virus and reduced viral p27 protein expression. 

**Figure 12:**
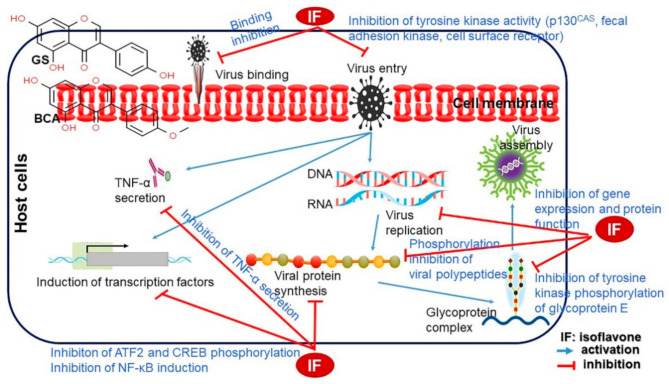
Antiviral effect of isoflavone (IF). IF inhibits virus binding and entry via tyrosine kinase inactivation into the host cells. IF also inhibits of secretion of tumor necrosis factor-alpha (TNF-α), phosphorylation of activating transcription factor 2 (ATF2), cyclic AMP-responsive element-binding protein (CREB), tyrosine, glycoprotein E, and viral polypeptides, as well as prevents the induction of nuclear factor kappa B (NF-κB) and activation of genes and proteins associated with virus assembly [300]. Figure adapted from Andres et al. [300].

In other words, although genistein did not prevent the host from being invaded by the virus, it blocked the transcription and release of the viral gene after infection. This is indicative of the inhibitory effect of genistein on the late phase of ALV-J replication (Figure 12) [299,300]. Biochanin A, a metabolite of the genistein, shows anti-avian influenza (AI) virus activity. Biochanin A was shown to effectively prevent the H5N1 virus while significantly reducing the cellular changes induced by the viral infection. Furthermore, by influencing the host cell signaling mechanisms, it effectively inhibited the replication of the H5N1 virus (Table S10) [301].

#### 4.18.4. Allergy Relief

The antibody IgE binds to the α-receptor on mast cells, causing allergic reactions. Ingestion of isoflavones, especially genistein, reduces α-receptors and has an anti-allergenic effect. In a study on animal models, it was reported that chronic atopic dermatitis was improved in mice fed with 4–20 mg/kg genistein per day for 8 weeks [184]. There are also reports showing a significant reduction in the incidence of allergic rhinitis in children who regularly consumed isoflavones (Table S10) [302].

### 4.19. Other Considerations

Intestinal bacteria play an important role in the activation of isoflavones; therefore, taking antibiotics may reduce the effect of isoflavones by half [5]. It has also been reported that ingesting high concentrations of isoflavones interferes with the effectiveness of anticoagulants such as warfarin. Further, in persons with thyroid disorders, isoflavones stimulate thyroid gland proliferation [52,303,304]. Thus, the amount of drugs required to regulate thyroid hormones, such as levothyroxine (used to treat hypothyroidism), would need to be increased to compensate [305,306]. 

### 4.20. Side Effects

Because legumes are a rich source of isoflavones, which are also known phytoestrogens, they are occasionally considered equivalent to isoflavones [307]. Recently, soybean consumption has been rapidly increasing in Western countries, where in the past it was only 1/20–1/50 of that in Asian countries. The mean daily isoflavone consumption per person in Japan is 30–50 mg, whereas that in the United States and Europe is of only few milligrams [307]. As discussed above, isoflavones are noteworthy substances as they have antioxidant activities that can help prevent cardiovascular disease, metabolic syndrome, and osteoporosis, and have a positive effect on menstrual irregularity in non-menopausal women, and alleviate climacteric symptoms. Despite its numerous benefits for humans, isoflavones are sometimes mentioned as endocrine disruptors based on animal studies, but this is not considered a significant problem owing to sufficient human data demonstrating the health benefits of isoflavones [307]. Recently, a comprehensive review of studies published until January 31, 2021, reported the relationship between endocrine-related parameters and legume and/or isoflavone intake [307]. Of 417 reports (229 observational studies, 157 clinical trials, 32 systematic reviews and meta-analyses) that met the inclusion criteria, none presented evidence suggesting that isoflavone intake induces thyroid functional impairment as a side effect [307]. Moreover, no adverse events were observed concerning breast or uterine endometrial tissues, and estrogen levels in women, as well as related to testosterone or estrogen levels, and sperm or semen-related parameters in men [307]. While the menstrual cycle may be extended slightly, ovulation occurs normally. There are limited insights into the effect of isoflavone exposure on the uterus and breast, but the consensus is that existing data confirm the safety of isoflavones [307]. Furthermore, there were no adverse events associated with isoflavone intake in children; however, few reports were available on this subject in pediatric populations [307].

Despite the functionality of isoflavones is in general well established, some side effects were reported, most of which were gastrointestinal events, such as nausea, abdominal distension, diarrhea, and constipation [12]. The side effects of *S*-equol reported in a previous animal study were relevant to isoflavones derived from other plants that not soybean. Sheep that consumed red clovers showed reproductive dysfunction, and captive cheetahs fed legume-containing feed became infertile [12,308]. Ovary-removed Sprague-Dawley rats fed a high concentration of equol developed a disease similar to that of breast cancer [12,309]. However, *S*-equol has been proven to be safe in the humans in all studies that discussed this subject.

In most cases, the side effects associated to isoflavones consumption are related to hormone imbalance due to estrogen receptor (ER) activity, particularly in human breasts. For example, glycoside genistin, such as aglycone genistein, can stimulate estrogen-dependent breast cancer cell growth. In contrast, a diet lacking genistin or genistein was reported to induce tumor degeneration [189,190]. However, because soybean products have a relatively low genistein and daidzein content, the increased risk for onset or progression of breast cancer can only be observed in relation to the intake of high-dose isoflavone supplements in women. Therefore, long-term human studies that monitor free estrogens and their conjugates reported that high-dose genistein and daidzein intake may potentially cause adverse events in patients diagnosed with ERα-positive, and not ERβ-positive, breast cancer [191]. Recently, as isoflavones can bind to ERβ, the perception of the adverse events caused by isoflavones as recently diminished [12,307].

Children in Asian countries traditionally consume large amounts of legumes, and Asian women are reported to have a high prevalence of endometriosis [193]. Mice fed legumes prior to puberty showed good growth, but periodic legume intake stimulated the development and progression of endometriosis, in addition to reproductive problems, in adulthood. In particular, a diet containing more than 10% legumes can potentially induce malignant conditions (e.g., malignant mixed Mullerian tumors) [194]. Women (18–49 years) who regularly consumed soybean formula during infancy were found to have more than 2-fold higher risk for endometriosis compared with women who have not been exposed to soybean formula [284]. Moreover, broad phytoestrogen supplementation facilitated ureteral Mullerian carcinosarcoma occurring in endometriosis lesions [284,310]. Furthermore, some studies suggested that early consumption of soybean-derived isoflavone foods increases the risk of endometriosis, whereas other studies focused on adult exposure raised doubts about the relationship between soybean consumption and endometriosis [307,311,312]. Regarding the risk of endometrial cancer, results on the protective effects of soybean isoflavones are more consistent. A meta-analysis that reported a negative correlation between soybean isoflavone consumption and the risk of endometrial cancer [307,313]. In addition to the individual impact on endometriosis, there are cases in which soybean isoflavone can weaken estrogen proliferation related to endometriosis [307,314,315,316]. Epidemiological and clinical trials do not confirm these case reports, but propose instead protective or null effects of isoflavones while considering key factors, such as estrogen exposure, timing of soybean exposure, and ethnic characteristics [307]. Considering the tremendous popularity and usefulness of dietary isoflavone, research on its safety in women with wide-ranging endometriosis is essential [194].

Gynecomastia, the enlargement of male breast tissue, has been reported in adult men as well as in teenagers, likely due to hormonal imbalance. The occurrence of this condition during puberty is suggested to be the result of physiological changes in androgen and estrogen levels, which naturally resolve in most cases over time [195]. Conversely, gynecomastia in prepubescent children is extremely rare and have been related to exogenous estrogen exposure, usually in the form of personal-care products containing tea tree oil or lavender, or accidental exposure to hormone-replacement therapies [195]. There is also a modest concern over whether excessive consumption of soy products may lead to the occurrence of gynecomastia [195]. Interestingly, there is the first documented case of gynecomastia in a prepubescent patient due to high intake of soy in the diet, and it can be resolved by limiting dietary products containing significant amounts of soy [195]. Ceasing soy milk intake after a daily intake of 3 quarts of soy milk in 60-year-old man, alleviated breast tenderness and improved normal estradiol concentration [196]. Although soybeans and soy-derived products can be a key source of nutrition for people, those with abnormal sensitivity to phytoestrogens may benefit by eliminating dietary soy intake to avoid potential adverse effects, such as gynecomastia [195,196]. There is no data available for the positive correlation between soy isoflavone and its adverse. Therefore, future studies are warranted to exhibit the way some diseases are caused by isoflavones.

## 5. Conclusions

The functionality of food products includes not only the nutrients they supply but also disease prevention and treatment. Isoflavones are phytoestrogens found in plants. They are originally a phytochemical with identical physiological activity to estrogens and they become physiologically active via the intestinal microflora, which have made them popular as an alternative female hormone treatment without side effects. Recently, as soybeans have attracted substantial interest as functional foods that can improve blood circulation and prevent osteoporosis, research into their effects has become more vigorous.

The main functionality of isoflavones discussed so far can be summarized as follows:

(1) Isoflavones can activate the estrogen receptor and may function as an estrogen or an antiestrogen depending on the physiological conditions or chemical structures.
(2) The anticancer activity of isoflavones comes mostly from genistein which is an antiestrogen that weakly binds to the estrogen receptor. Genistein reduces cancer cell proliferation and promotes the division of normal cells. Genistein also inhibits angiogenesis to block the supply of oxygen or nutrients, thereby suppressing tumor development.
(3) Isoflavones in vitro and in vivo show antioxidant effects equivalent those of vitamins E and C.
(4) The estrogen effect of isoflavones can be utilize to improve the physical and emotional symptoms in post-menopausal women.
(5) While the animal protein casein increases plasma cholesterol levels, soy isoflavone decreases them, indicating its potential use as a cholesterol lowering agent.
(6) Isoflavones prevent calcium leaks from the bones by elevating vitamin D activity that enhances calcium absorption, thus lowering the risk of osteoporosis.
(7) The reported effects of isoflavones include reducing blood pressure, enhancing the effects of lipid metabolism, anti-mutational effects, antimicrobial activity, antiviral activity, and skin protective effects.

This review has focused on isoflavone bioactivity that has been identified so far and not previous studies on the nutritional quality enhancements of soybean-derived isoflavones, with an aim to examine the possibility of their use in functional food products. Most studies so far were investigations in cultured cells or experimental animals using high doses of isoflavones that exceed the average human consumption by several tens or hundreds of times. Such doses of isoflavones are impossible with general food intake. In addition, applying the results of animal experiments to clinical studies is rather difficult because the sensitivity to isoflavones varies with race, age, and individual characteristics while different results may also be obtained because of the form, dose, and exposure of the samples [317]. Despite such practical constraints, it is undisputable that soybean-derived isoflavones have the potential to enhance the quality of life, but further studies to clarify the mechanisms of isoflavone activity are required.

Isoflavones have been safely consumed in different food sources in Asian countries, including South Korea for centuries. Some studies show that 75% of Asians consume an average of 65 mg of isoflavones per day. Long-term consumption of isoflavones is considered safe, and it has various beneficial effects, but further research is needed to determine the side effects of high isoflavone intake [318,319]. The amount of isoflavone consumed in the Asian diet may be the most reasonable, as the US FDA’s recommended intake is 50 mg/day [12,101]. The effects of isoflavones on each of the health aspects dealt with in this review are still being debated and need further consideration. It should be noted that I have reviewed only the positive results that have been reported so far. However, no serious side effects of isoflavone intake have been identified yet.

## 6. Perspectives

Soybeans have been reported to be beneficial for human heath due to their nutritional and functional characteristics. Recently, they have been under the spotlight due to the physiological characteristics of isoflavones found in them. Isoflavones, which are related to root nodule formation for nitrogen fixation in the rhizosphere, are commonly found in Fabaceae, including soybeans. This review summarizes various current research trends, including content, mechanisms for nitric oxide synthesis, bioactivity in plants, and potential functionality for human health, on isoflavones and their metabolites that constitute the non-nutrient compounds of soybeans. Consumption of foods containing isoflavones such as tofu, boiled beans, natto, soybean paste, and tempering have evolved as traditional fermented foods in East Asia, including South Korea. Moreover, the consumption of isoflavone-derived soy-based foods has recently expanded; it is crucial in terms of preventing of various diseases. As mentioned in this review, soybeans containing isoflavones have been reported to have a wide variety of health effects. As a result, consumers are increasingly recognizing how beneficial they are for their health. However, the fact that soybeans are the only raw materials rich in isoflavones limits the types of foods that contain them. Furthermore, since most of them are traditional, soy-based food intake decreases as dietary habits in South Korea become westernized, where soy-based foods are abundant. Since isoflavones are very stable to heat and pH dependent, various compounds are required to be developed through the addition of isoflavone-containing soybeans to generic or health functional foods, other than traditional ones, as an alternative to overcome this problem.

Conversely, as Korea has become an aged society, many elderly people suffer from a stroke and osteoporosis; this leads to increasing numbers of bedridden patients or those with vascular dementia, thereby acutely exacerbating their quality of life. The role of soybeans in preventing or treating these diseases is largely increasing, as part of becoming a “happy bluebird”. In addition, consumption of soybeans, a high-nutrient source for longevity among other traditional foods in South Korea and worldwide, will be essential to prevent lifestyle-related diseases in many countries during the 21st century. Indeed, humankind already holds the key to a healthy lifestyle. This article explains key details; I hope that further research and investigation on the effects and use of isoflavones will follow, thereby ushering in for a disease-free life expectancy of humankind. Although isoflavones have been studied relatively well, this review also aims to suggest that many classes of interactions between isoflavones and biopolymers remain to be clarified.

## Supplementary Materials

The following are available online at https://www.mdpi.com/article/10.3390/antiox10071064/s1, Table S1: Experimental design strategy, Table S2: Neuroprotection and antidepressant effects of isoflavone and its metabolites, Table S3: Anti-obesity and effects on improving metabolic syndrome and regulation of blood pressure effects of isoflavone and its metabolites, Table S4: Improvement of cardiac function, protection against liver damage, and improvement of renal dysfunction of isoflavone and its metabolites, Table S5: Anti-inflammatory effects of isoflavone and its metabolites, Table S6: Anticancer effects of isoflavone and its metabolites, Table S7: Anti-osteoporosis and anti-diabetes effects of isoflavone and its metabolites, Table S8: Skin improvement of isoflavone and its metabolites, Table S9: Enhanced quality of life for postmenopausal women and safety in reproductive development of isoflavone and its metabolites, Table S10: Improvement of arteriosclerosis and antioxidant activity, and antiviral and anti-allergic effects of isoflavone and its metabolites.

## Abbreviations

AA	arachidonic acid
ABC	ATP-binding cassette
AC	adenylyl cyclase
ACC	acetyl-CoA carboxylase
ADRP	adipose differentiation-related protein activator protein 1
AhR	aryl hydrocarbon receptor
AICD	APP intracellular domain
Akt	protein kinase B (serine/threonine-specific protein kinase)
ALP	alkaline phosphatase
AMPK	5′-adenosine monophosphate-activated protein kinase
ANS	anthocyanidin synthase
AP1	activator protein 1
APP	amyloid precursor protein
AR	androgen receptor
ARE	antioxidant responsive element
AT	acetyltransferase
ATF2	activating transcription factor 2 ATP, adenosine triphosphate
BAD	Bcl2-associated agonist of cell death
B.Ar	bone area
BCA	biochanin A
BMI	body mass index
BMP2	bone morphogenetic protein 2
BV/TV	bone volume/tissue volume
Ca^2+^	calcium ion
C/EBP	CCAAT/enhancer-binding proteins
CaMK II	calmodulin kinase II
cAMP	cyclic adenosine monophosphate
C/EBP-α	CCAAT/enhancer-binding protein-alpha
c-Fos	cellular oncogene fos
C4H	cinnamic acid 4-hydroxylae
CHI	chalcone isomerase
CHR	chalcone reductase
CHS	chalcone synthase
4CL	4-coumarate-CoA ligase
COL1a1	transcription factor Sp7 (Osterix), alpha-1 type I collagen
COX-2	cyclooxygenase 2
CRE	carbohydrate response element
CREBp	cyclic AMP-responsive element-binding protein
CTF	C-terminal fragment
CTSK	cathepsin K
DFR	dihydroxyflavonol reductase
DMID	7,2′-dihydroxy-4′-methoxy-isoflavanol dehydratase
DHD	dihydrodaidzein
DR5	tumor necrosis factor (TNF)-related apoptosis-inducing ligand (TRAIL) receptor 2
DZ	daidzein
EGF	epidermal growth factor
EGFR	epidermal growth factor receptor
eNOS	endothelial nitric oxide synthase
EPR	endoplasmic reticulum
ER	estrogen receptor
ERAα/β	estrogen receptor A alpha/beta
ERα	estrogen receptor alpha
ERβ	estrogen receptor beta
ERK1/2	extracellular signal-regulated kinase 1 and 2
F3H	flavanone 3-hydroxylase
FADD	Fas-associated protein with death domain
FAS	fatty acid synthetase
FASL (CD95L)	FAS ligand
PARP	poly(ADP-ribose)-polymerase
FBG	fasting blood glucose
FFAs	free fatty acids
FLS	flavonol synthase
FNS	flavone synthase
FNT	formononetin
FOXO	forkhead box transcription factor family O
G2′DT	glycinol 2-dimethylallyltransferase
G4′DT	glycinol 4-dimethylallyltransferase
G6Pase	glucose-6-phosphatase
GC	glycetin
GDNF	glial cell line-derived neurotrophic factor
GFr	growth factor receptor
GK	glucokinase
GLS	glyceollin synthase
GLUT4	glucose transporter type 4
GPX	glutathione peroxidase
gp91 (NOX2)	NADPH oxidase subunit
G or GS	genistein
GSH	glutathione (reduced form)
GSK3β	glycogen synthase kinase 3 beta
GT	glucosyltransferase
6-HD	6-hydroxydaidzein
8-HD	8-hydroxydaidzein
12′H	isoflavone hydroxylase
HER2	human epidermal growth factor receptor 2
HI4′OMT	2,7,4′-trihydroxyisoflavanone 4′-*O*-methyltransferase
HID	2-hydroxyisoflavone dehydratase
2-HIS	2-hydroxyisoflavone synthase
HNF4α	hepatocyte nuclear factor 4 alpha
HO-1	heme oxygenase 1
I2′H	isoflavone 2-hydroxylase
ICAM-1	intracellular adhesion molecule 1
ICHG	isoflavone conjugate-hydrolyzing β-glucosidase
IF	isoflavone
IF7GT	isoflavone-7-*O*-glucosyltransferase
IF7MaT	isoflavone-7-*O*-glucoside-6″-*O*-malonyltransferase
IFH	isoflavone hydroxylase
IFR	isoflavone reductase
IFS	isoflavone synthase
IκB	inhibitory protein of κB family
IKK	IκB kinase
IL-1	interleukin 1
IMT	isoflavone malonyl transferase
iNOS	inducible nitric oxide synthases
INSR	insulin receptor
IRβ	insulin receptor beta
IRS1/2	insulin receptor substrate-1 and 2
ISN1	inosine 5′-monophosphate (IMP)-specific 5′-nucleotidase
JAK2	Janus kinase 2
LAR	flavan-3,4-diol reductase
LDL	low-density lipoprotein
LOX	lipoxygenase
LPS	lipopolysaccharide
Maf	musculoaponeurotic fibrosarcoma protein
MAPK	mitogen-activated protein kinase
MEK	mitogen-activated protein kinase kinase
MKK1/7	mitogen-activated protein kinase kinase 1 and 7
MMP	matrix metalloproteinase
MT	malonyltransferase
MCP1	monocyte chemoattractant protein 1
MDA	malonaldehyde
MGO	methylglyoxal
NAD^+^	nicotinamide adenine dinucleotide (oxidized form)
NADPH	nicotinamide adenine dinucleotide phosphate (reduced form)
NFATc1	nuclear factor of activated T-cells cytoplasmic 1
NFIL3	nuclear factor interleukin-3-regulated protein
NF-κB	nuclear factor-kappa B
NGF	nerve growth factor
NIK	NF-κB-inducing kinase
nNOS	neuronal isoform of nitric oxide synthase
NO	nitrix oxide
NOX	NADPH oxidase
NOX4	NAPDH oxidase 4
NQO1	NAD(P)H quinone oxidoreductase 1
Nrf2	nuclear factor erythroid 2
OCN	osteocalcin
OPG	osteoprotegerin
PAL	phenylalanine ammonia lyase
p-AMPK	phosphorylated adenosine 5′-monophosphate-activated protein kinase
PARP	poly(ADP-ribose)-polymerase
PDK1/2	3-phosphoinositide–dependent protein kinase 1 and 2
PEPCK	phosphoenolpyruvate carboxykinase
p-ERK	phosphorylated extracellular signal-regulated kinase
PG	prostaglandin
PGC-1α	peroxisome proliferator-activated receptor-gamma coactivator-1alpha
PGE_2_	prostaglandin E2
PGF_2_	prostaglandin F2
PI3K	phosphatidylinositol 3-kinase
PIP2	phosphatidylinositol-4,5-biphosphate
PIP3	phosphatidylinositol-3,4,5-triphosphate
PKA	protein kinase A (cAMP-dependent protein kinase)
PKC	protein kinase C
PLA2	phospholipase A2
PPARα	peroxisome proliferator-activated receptor-alpha
PPARγ	peroxisome proliferator-activated receptors-gamma
PTS	pterocarpan synthase
PYK	pyruvate kinase
RANKL	receptor activator of NF-κB ligand
RIP	receptor-interacting protein
ROS	reactive oxygen species
RUNX2	runt-related transcription factor 2
S1P and S2P	site-specific protease 1 and 2
sAPP	soluble APP
SCAP	SREB cleavage-activating protein
SIRT1	sirtuin 1
SCD1	stearoyl-CoA desaturase 1
SGK1	serine/threonine-protein kinase
SMI	structure model index
SOD	superoxide dismutase
SREB	sterol regulatory element binding
STAT1	signal transducer and activator of transcription 1
STEBp1	SET-binding protein 1
T3	triiodothyronine
T4	thyroxine
TAK1	transforming growth factor β-activated kinase
TAT1	signal transducer and activator of transcription 1
TBARs	thiobarbituric acid reactive substances
Tb.N	trabecular number
Tb.Pf	trabecular pattern factor
Tb.Sp	trabecular separation
Tb.Th	trabecular thickness
TCF	T cell factor
TG	Triacylglycerol
TGF-α	transforming growth factor-alpha
TNF	tumor necrosis factor
TNF-α	tumor necrosis factor-alpha
TNFR	tumor necrosis factor receptor
TNFR1	TNF receptor-1
TORC2	target of rapamycin complex 2
TRADD	TNF receptor-associated death domain
TRAF2	TNF receptor-associated factor 2
TRAIL	TNF-related apoptosis-inducing ligand
TRAP	tartrate-resistant acid phosphatase
Ub	ubiquitination
UCP1	uncoupling protein 1
VR	vestitone reductase
Wnt/β-catenin	canonical Wnt pathway
XRE	xenobiotic response element

## References

[1] Calaf G.M., Ponce-Cusi R., Aguayo F., Munoz J.P., Bleak T.C. (2020). Endocrine disruptors from the environment affecting breast cancer. Oncol. Lett..

[2] Sirtori C.R. (2001). Risks and benefits of soy phytoestrogens in cardiovascular diseases, cancer, climacteric symptoms and osteoporosis. Drug. Saf..

[3] Petrine J.C.P., Del Bianco-Borges B. (2021). The influence of phytoestrogens on different physiological and pathological processes: An overview. Phytother. Res..

[4] Bacciottini L., Falchetti A., Pampaloni B., Bartolini E., Carossino A.M., Brandi M.L. (2007). Phytoestrogens: Food or drug?. Clin. Cases Miner. Bone Metab..

[5] Krizova L., Dadakova K., Kasparovska J., Kasparovsky T. (2019). Isoflavones. Molecules.

[6] Pabich M., Materska M. (2019). Biological effect of soy isoflavones in the prevention of civilization diseases. Nutrients.

[7] Kim I.S., Hwang C.W., Yang W.S., Kim C.H. (2021). Current perspectives on the physiological activities of fermented soybean-derived cheonggukjang. Int. J. Mol. Sci..

[8] Kim I.S., Kim C.H., Yang W.S. (2021). Physiologically active molecules and functional properties of soybeans in human health—A current perspective. Int. J. Mol. Sci..

[9] Szeja W., Grynkiewicz G., Rusin A. (2017). Isoflavones, their glycosides and glycoconjugates. Synthesis and biological activity. Curr. Org. Chem..

[10] Kim J.S., Lee J.H., Surh J., Kang S.A., Jang K.H. (2016). Aglycone isoflavones and exopolysaccharides produced by *Lactobacillus acidophilus* in fermented soybean paste. Prev. Nutr. Food Sci..

[11] Panche A.N., Diwan A.D., Chandra S.R. (2016). Flavonoids: An overview. J. Nutr. Sci..

[12] Chen L.R., Ko N.Y., Chen K.H. (2019). Isoflavone supplements for menopausal women: A Systematic review. Nutrients.

[13] Miadokova E. (2009). Isoflavonoids—An overview of their biological activities and potential health benefits. Interdiscip. Toxicol..

[14] Lecomte S., Demay F., Ferriere F., Pakdel F. (2017). Phytochemicals targeting estrogen receptors: Beneficial rather than adverse effects?. Int. J. Mol. Sci..

[15] Vitale D.C., Piazza C., Melilli B., Drago F., Salomone S. (2013). Isoflavones: Estrogenic activity, biological effect and bioavailability. Eur. J. Drug Metab. Pharmacokinet..

[16] He F.J., Chen J.Q. (2013). Consumption of soybean, soy foods, soy isoflavones and breast cancer incidence: Differences between Chinese women and women in Western countries and possible mechanisms. Food Sci. Hum. Well..

[17] Garcia-Calderon M., Perez-Delgado C.M., Palove-Balang P., Betti M., Marquez A.J. (2020). Flavonoids and isoflavonoids biosynthesis in the model legume *Lotus japonicus*; Connections to nitrogen metabolism and photorespiration. Plants.

[18] Gupta O.P., Nigam D., Dahuja A., Kumar S., Vinutha T., Sachdev A., Praveen S. (2017). Regulation of isoflavone biosynthesis by miRNAs in two contrasting soybean genotypes at different seed developmental stages. Front. Plant Sci..

[19] Yu O., Jung W., Shi J., Croes R.A., Fader G.M., McGonigle B., Odell J.T. (2000). Production of the isoflavones genistein and daidzein in non-legume dicot and monocot tissues. Plant Physiol..

[20] Akashi T., Aoki T., Ayabe S. (2005). Molecular and biochemical characterization of 2-hydroxyisoflavanone dehydratase. Involvement of carboxylesterase-like proteins in leguminous isoflavone biosynthesis. Plant Physiol..

[21] Ahmad M.Z., Li P., Wang J., Rehman N.U., Zhao J. (2017). Isoflavone malonyltransferases GmIMaT1 and GmIMaT3 differently modify isoflavone glucosides in soybean (*Glycine max*) under various stresses. Front. Plant Sci..

[22] Sugiyama A., Yamazaki Y., Hamamoto S., Takase H., Yazaki K. (2017). Synthesis and secretion of isoflavones by field-grown soybean. Plant Cell Physiol..

[23] Falcone Ferreyra M.L., Rius S.P., Casati P. (2012). Flavonoids: Biosynthesis, biological functions, and biotechnological applications. Front. Plant Sci..

[24] Dong N.Q., Lin H.X. (2021). Contribution of phenylpropanoid metabolism to plant development and plant-environment interactions. J. Integr. Plant Biol..

[25] Sarkar M.A.R., Watanabe S., Suzuki A., Hashimoto F., Anai T. (2019). Identification of novel MYB transcription factors involved in the isoflavone biosynthetic pathway by using the combination screening system with agroinfiltration and hairy root transformation. Plant Biotechnol..

[26] Chu S., Wang J., Zhu Y., Liu S., Zhou X., Zhang H., Wang C.E., Yang W., Tian Z., Cheng H. (2017). An R2R3-type MYB transcription factor, GmMYB29, regulates isoflavone biosynthesis in soybean. PLoS Genet..

[27] Izumi T., Piskula M.K., Osawa S., Obata A., Tobe K., Saito M., Kataoka S., Kubota Y., Kikuchi M. (2000). Soy isoflavone aglycones are absorbed faster and in higher amounts than their glucosides in humans. J. Nutr..

[28] Andres S., Hansen U., Niemann B., Palavinskas R., Lampen A. (2015). Determination of the isoflavone composition and estrogenic activity of commercial dietary supplements based on soy or red clover. Food Funct..

[29] Dixon R.A. (2001). Natural products and plant disease resistance. Nature.

[30] Singh S., Kaur I., Kariyat R. (2021). The Multifunctional roles of polyphenols in plant-herbivore interactions. Int. J. Mol. Sci..

[31] Jayaraman D., Forshey K.L., Grimsrud P.A., Ane J.M. (2012). Leveraging proteomics to understand plant-microbe interactions. Front. Plant Sci..

[32] Olanrewaju O.S., Ayangbenro A.S., Glick B.R., Babalola O.O. (2019). Plant health: Feedback effect of root exudates-rhizobiome interactions. Appl. Microbiol. Biotechnol..

[33] Sukumaran A., McDowell T., Chen L., Renaud J., Dhaubhadel S. (2018). Isoflavonoid-specific prenyltransferase gene family in soybean: GmPT01, a pterocarpan 2-dimethylallyltransferase involved in glyceollin biosynthesis. Plant J..

[34] Yu J., Bi X., Yu B., Chen D. (2016). Isoflavones: Anti-inflammatory benefit and possible caveats. Nutrients.

[35] Testa I., Salvatori C., Di Cara G., Latini A., Frati F., Troiani S., Principi N., Esposito S. (2018). Soy-based infant formula: Are phyto-oestrogens still in doubt?. Front. Nutr..

[36] Dutta S., Mitra M., Agarwal P., Mahapatra K., De S., Sett U., Roy S. (2018). Oxidative and genotoxic damages in plants in response to heavy metal stress and maintenance of genome stability. Plant Signal. Behav..

[37] Skalicky M., Kubes J., Hejnak V., Tumova L., Martinkova J., Martin J., Hnilickova H. (2018). Isoflavones production and possible mechanism of their exudation in *Genista tinctoria* L. suspension culture after treatment with vanadium compounds. Molecules.

[38] Kubes J., Skalicky M., Tumova L., Martin J., Hejnak V., Martinkova J. (2019). Vanadium elicitation of *Trifolium pratense* L. cell culture and possible pathways of produced isoflavones transport across the plasma membrane. Plant Cell Rep..

[39] Pessoa J.C., Etcheverry S., Gambino D. (2015). Vanadium compounds in medicine. Coord. Chem. Rev..

[40] Romera F.J., Garcia M.J., Lucena C., Martinez-Medina A., Aparicio M.A., Ramos J., Alcantara E., Angulo M., Perez-Vicente R. (2019). Induced systemic resistance (ISR) and Fe deficiency responses in dicot plants. Front. Plant Sci..

[41] Conrath U. (2006). Systemic acquired resistance. Plant Signal. Behav..

[42] Jaiswal S.K., Mohammed M., Ibny F.Y.I., Dakora F.D. (2021). Rhizobia as a source of plant growth-promoting molecules: Potential applications and possible operational mechanisms. Front. Sustain. Food Syst..

[43] Toth K., Stacey G. (2015). Does plant immunity play a critical role during initiation of the legume-rhizobium symbiosis?. Front. Plant Sci..

[44] Cowan M.M. (1999). Plant products as antimicrobial agents. Clin. Microbiol. Rev..

[45] Gonzalez-Lamothe R., Mitchell G., Gattuso M., Diarra M.S., Malouin F., Bouarab K. (2009). Plant antimicrobial agents and their effects on plant and human pathogens. Int. J. Mol. Sci..

[46] Lygin A.V., Zernova O.V., Hill C.B., Kholina N.A., Widholm J.M., Hartman G.L., Lozovaya V.V. (2013). Glyceollin is an important component of soybean plant defense against *Phytophthora sojae* and *Macrophomina phaseolina*. Phytopathology.

[47] Abdel-Lateif K., Bogusz D., Hocher V. (2012). The role of flavonoids in the establishment of plant roots endosymbioses with arbuscular mycorrhiza fungi, rhizobia and Frankia bacteria. Plant Signal. Behav..

[48] Sugiyama A. (2019). The soybean rhizosphere: Metabolites, microbes, and beyond—A review. J. Adv. Res..

[49] Rice-Evans C. (2004). Flavonoids and isoflavones: Absorption, metabolism, and bioactivity. Free Radic. Biol. Med..

[50] Setchell K.D. (2000). Absorption and metabolism of soy isoflavones-from food to dietary supplements and adults to infants. J. Nutr..

[51] Nanashima N., Horie K., Maeda H. (2017). Phytoestrogenic activity of blackcurrant anthocyanins is partially mediated through estrogen receptor beta. Molecules.

[52] Huser S., Guth S., Joost H.G., Soukup S.T., Kohrle J., Kreienbrock L., Diel P., Lachenmeier D.W., Eisenbrand G., Vollmer G. (2018). Effects of isoflavones on breast tissue and the thyroid hormone system in humans: A comprehensive safety evaluation. Arch. Toxicol..

[53] Simons R., Gruppen H., Bovee T.F., Verbruggen M.A., Vincken J.P. (2012). Prenylated isoflavonoids from plants as selective estrogen receptor modulators (phytoSERMs). Food Funct..

[54] Amaral C., Toloi M.R.T., Vasconcelos L.D., Fonseca M.J.V., Correia-da-Silva G., Teixeira N. (2017). The role of soybean extracts and isoflavones in hormone-dependent breast cancer: Aromatase activity and biological effects. Food Funct..

[55] Lu C., Wang Y., Wang D., Zhang L., Lv J., Jiang N., Fan B., Liu X., Wang F. (2018). Neuroprotective effects of soy isoflavones on scopolamine-induced amnesia in mice. Nutrients.

[56] Lu Y., An Y., Lv C., Ma W., Xi Y., Xiao R. (2018). Dietary soybean isoflavones in Alzheimer’s disease prevention. Asia Pac. J. Clin. Nutr..

[57] Uddin M.S., Kabir M.T. (2019). Emerging signal regulating potential of genistein against Alzheimer’s disease: A promising molecule of interest. Front. Cell Dev. Biol..

[58] Moran J., Garrido P., Cabello E., Alonso A., Gonzalez C. (2014). Effects of estradiol and genistein on the insulin signaling pathway in the cerebral cortex of aged female rats. Exp. Gerontol..

[59] Liao W., Jin G., Zhao M., Yang H. (2013). The effect of genistein on the content and activity of alpha- and beta-secretase and protein kinase C in Abeta-injured hippocampal neurons. Basic Clin. Pharmacol. Toxicol..

[60] Jiang T., Wang X.Q., Ding C., Du X.L. (2017). Genistein attenuates isoflurane-induced neurotoxicity and improves impaired spatial learning and memory by regulating cAMP/CREB and BDNF-TrkB-PI3K/Akt signaling. Korean J. Physiol. Pharmacol..

[61] Ko Y.H., Kim S.Y., Lee S.Y., Jang C.G. (2018). 6,7,4’-Trihydroxyisoflavone, a major metabolite of daidzein, improves learning and memory via the cholinergic system and the p-CREB/BDNF signaling pathway in mice. Eur. J. Pharmacol..

[62] Rizzo G. (2020). The antioxidant role of soy and soy foods in human health. Antioxidants.

[63] Messina M., Gleason C. (2016). Evaluation of the potential antidepressant effects of soybean isoflavones. Menopause.

[64] Thangavel P., Puga-Olguín A., Rodríguez-Landa J.F., Zepeda R.C. (2019). Genistein as potential therapeutic candidate for menopausal symptoms and other related diseases. Molecules.

[65] Clark L.A., Cuthbert B., Lewis-Fernandez R., Narrow W.E., Reed G.M. (2017). Three approaches to understanding and classifying mental disorder: ICD-11, DSM-5, and the National Institute of Mental Health’s Research Domain Criteria (RDoC). Psychol. Sci. Public Interest..

[66] Hu P., Ma L., Wang Y.G., Ye F., Wang C., Zhou W.H., Zhao X. (2017). Genistein, a dietary soy isoflavone, exerts antidepressant-like effects in mice: Involvement of serotonergic system. Neurochem. Int..

[67] Miyake Y., Tanaka K., Okubo H., Sasaki S., Furukawa S., Arakawa M. (2018). Soy isoflavone intake and prevalence of depressive symptoms during pregnancy in Japan: Baseline data from the Kyushu Okinawa Maternal and Child Health Study. Eur. J. Nutr..

[68] Hruby A., Hu F.B. (2015). The epidemiology of obesity: A big picture. Pharmacoeconomics.

[69] Pischon T., Nothlings U., Boeing H. (2008). Obesity and cancer. Proc. Nutr. Soc..

[70] Nakai S., Fujita M., Kamei Y. (2020). Health promotion effects of soy isoflavones. J. Nutr. Sci. Vitaminol..

[71] Wang S., Wang Y., Pan M.H., Ho C.T. (2017). Anti-obesity molecular mechanism of soy isoflavones: Weaving the way to new therapeutic routes. Food Funct..

[72] Kopp W. (2019). How western diet and lifestyle drive the pandemic of obesity and civilization diseases. Diabetes Metab. Syndr. Obes..

[73] Lu Y., Zhao A., Wu Y., Zhao Y., Yang X. (2019). Soybean soluble polysaccharides enhance bioavailability of genistein and its prevention against obesity and metabolic syndrome of mice with chronic high fat consumption. Food Funct..

[74] Grossini E., Farruggio S., Raina G., Mary D., Deiro G., Gentilli S. (2018). Effects of genistein on differentiation and viability of human visceral adipocytes. Nutrients.

[75] Shin H.W., Jang E.S., Moon B.S., Lee J.J., Lee D.E., Lee C.H., Shin C.S. (2016). Anti-obesity effects of gochujang products prepared using rice koji and soybean meju in rats. J. Food Sci. Technol..

[76] Aziz S.A., Wakeling L.A., Miwa S., Alberdi G., Hesketh J.E., Ford D. (2017). Metabolic programming of a beige adipocyte phenotype by genistein. Mol. Nutr. Food Res..

[77] Huang C., Pang D., Luo Q., Chen X., Gao Q., Shi L., Liu W., Zou Y., Li L., Chen Z. (2016). Soy isoflavones regulate lipid metabolism through an AKT/mTORC1 pathway in diet-induced obesity (DIO) male rats. Molecules.

[78] Hsiao Y.H., Ho C.T., Pan M.H. (2020). Bioavailability and health benefits of major isoflavone aglycones and their metabolites. J. Funct. Foods.

[79] Kurrat A., Blei T., Kluxen F.M., Mueller D.R., Piechotta M., Soukup S.T., Kulling S.E., Diel P. (2015). Lifelong exposure to dietary isoflavones reduces risk of obesity in ovariectomized Wistar rats. Mol. Nutr. Food Res..

[80] Wang M. (2005). The role of glucocorticoid action in the pathophysiology of the Metabolic Syndrome. Nutr. Metab..

[81] Tagawa N., Kubota S., Kobayashi Y., Kato I. (2015). Genistein inhibits glucocorticoid amplification in adipose tissue by suppression of 11beta-hydroxysteroid dehydrogenase type 1. Steroids.

[82] Kurylowicz A., Cakala-Jakimowicz M., Puzianowska-Kuznicka M. (2020). Targeting abdominal obesity and its complications with dietary phytoestrogens. Nutrients.

[83] Kurylowicz A. (2020). The role of isoflavones in type 2 diabetes prevention and treatment-A narrative review. Int. J. Mol. Sci..

[84] Abdelrazek H.M.A., Mahmoud M.M.A., Tag H.M., Greish S.M., Eltamany D.A., Soliman M.T.A. (2019). Soy isoflavones ameliorate metabolic and immunological alterations of ovariectomy in female Wistar rats: Antioxidant and estrogen sparing potential. Oxid. Med. Cell Longev..

[85] Rotundo L., Persaud A., Feurdean M., Ahlawat S., Kim H.S. (2018). The association of leptin with severity of non-alcoholic fatty liver disease: A population-based study. Clin. Mol. Hepatol..

[86] Arias-Loste M.T., Ranchal I., Romero-Gomez M., Crespo J. (2014). Irisin, a link among fatty liver disease, physical inactivity and insulin resistance. Int. J. Mol. Sci..

[87] Martinez-Una M., Lopez-Mancheno Y., Dieguez C., Fernandez-Rojo M.A., Novelle M.G. (2020). Unraveling the role of leptin in liver function and its relationship with liver diseases. Int. J. Mol. Sci..

[88] Hemati N., Asis M., Moradi S., Mollica A., Stefanucci A., Nikfar S., Mohammadi E., Farzaei M.H., Abdollahi M. (2020). Effects of genistein on blood pressure: A systematic review and meta-analysis. Food Res. Int..

[89] Huang P.L. (2009). A comprehensive definition for metabolic syndrome. Dis. Model. Mech..

[90] Lim S., Shin H., Song J.H., Kwak S.H., Kang S.M., Yoon J.W., Choi S.H., Cho S.I., Park K.S., Lee H.K. (2011). Increasing prevalence of metabolic syndrome in Korea: The Korean National Health and Nutrition Examination Survey for 1998–2007. Diabetes Care.

[91] Jalili M., Vahedi H., Poustchi H., Hekmatdoost A. (2019). Soy isoflavones and cholecalciferol reduce inflammation, and gut permeability, without any effect on antioxidant capacity in irritable bowel syndrome: A randomized clinical trial. Clin. Nutr. ESPEN.

[92] Abron J.D., Singh N.P., Price R.L., Nagarkatti M., Nagarkatti P.S., Singh U.P. (2018). Genistein induces macrophage polarization and systemic cytokine to ameliorate experimental colitis. PLoS ONE.

[93] Liu J., Chang R., Zhang X., Wang Z., Wen J., Zhou T. (2018). Non-isoflavones diet incurred metabolic modifications induced by constipation in rats via targeting gut microbiota. Front. Microbiol..

[94] Paley C.A., Johnson M.I. (2018). Abdominal obesity and metabolic syndrome: Exercise as medicine?. BMC Sports Sci. Med. Rehabil..

[95] Silva H. (2021). The vascular effects of isolated isoflavones—A focus on the determinants of blood pressure regulation. Biology.

[96] Buttar H.S., Li T., Ravi N. (2005). Prevention of cardiovascular diseases: Role of exercise, dietary interventions, obesity and smoking cessation. Exp. Clin. Cardiol..

[97] Harsha D.W., Bray G.A. (2008). Weight loss and blood pressure control (Pro). Hypertension.

[98] Lu L.W., Chen N.W., Nayeem F., Nagamani M., Anderson K.E. (2020). Soy isoflavones interact with calcium and contribute to blood pressure homeostasis in women: A randomized, double-blind, placebo controlled trial. Eur. J. Nutr..

[99] Lu J.F., Chen C.M., Hsu C.Y. (2019). Effect of home telehealth care on blood pressure control: A public healthcare centre model. J. Telemed. Telecare.

[100] Richardson S.I., Steffen L.M., Swett K., Smith C., Burke L., Zhou X., Shikany J.M., Rodriguez C.J. (2016). Dietary total isoflavone intake is associated with lower systolic blood pressure: The coronary artery risk development in young adults (CARDIA) study. J. Clin. Hypertens..

[101] Chen L.R., Chen K.H. (2021). Utilization of isoflavones in soybeans for women with menopausal syndrome: An overview. Int. J. Mol. Sci..

[102] Lee H., Choue R., Lim H. (2017). Effect of soy isoflavones supplement on climacteric symptoms, bone biomarkers, and quality of life in Korean postmenopausal women: A randomized clinical trial. Nutr. Res. Pract..

[103] Im J., Park K. (2021). Association between soy food and dietary soy isoflavone intake and the risk of cardiovascular disease in women: A prospective cohort study in Korea. Nutrients.

[104] Gorzkiewicz J., Bartosz G., Sadowska-Bartosz I. (2021). The potential effects of phytoestrogens: The role in neuroprotection. Molecules.

[105] Mirmiran P., Bahadoran Z., Azizi F. (2014). Functional foods-based diet as a novel dietary approach for management of type 2 diabetes and its complications: A review. World J. Diabetes.

[106] Qin W., Du N., Zhang L., Wu X., Hu Y., Li X., Shen N., Li Y., Yang B., Xu C. (2015). Genistein alleviates pressure overload-induced cardiac dysfunction and interstitial fibrosis in mice. Br. J. Pharmacol..

[107] Tsai Y.C., Leu S.Y., Peng Y.J., Lee Y.M., Hsu C.H., Chou S.C., Yen M.H., Cheng P.Y. (2017). Genistein suppresses leptin-induced proliferation and migration of vascular smooth muscle cells and neointima formation. J. Cell. Mol. Med..

[108] Qin Y., Shu F., Zeng Y., Meng X., Wang B., Diao L., Wang L., Wan J., Zhu J., Wang J. (2014). Daidzein supplementation decreases serum triglyceride and uric acid concentrations in hypercholesterolemic adults with the effect on triglycerides being greater in those with the GA compared with the GG genotype of ESR-beta RsaI. J. Nutr..

[109] Preston R.A. (2007). Effects of blood pressure reduction on cardiovascular risk estimates in hypertensive postmenopausal women. Climacteric.

[110] Sathyapalan T., Aye M., Rigby A.S., Thatcher N.J., Dargham S.R., Kilpatrick E.S., Atkin S.L. (2018). Soy isoflavones improve cardiovascular disease risk markers in women during the early menopause. Nutr. Metab. Cardiovasc. Dis..

[111] Tang G.Y., Meng X., Li Y., Zhao C.N., Liu Q., Li H.B. (2017). Effects of vegetables on cardiovascular diseases and related mechanisms. Nutrients.

[112] Wong W.W., Taylor A.A., Smith E.O., Barnes S., Hachey D.L. (2012). Effect of soy isoflavone supplementation on nitric oxide metabolism and blood pressure in menopausal women. Am. J. Clin. Nutr..

[113] Jang J.Y., Kim D.J. (2018). Epidemiology of alcoholic liver disease in Korea. Clin. Mol. Hepatol..

[114] Xiao C.W., Wood C.M., Weber D., Aziz S.A., Mehta R., Griffin P., Cockell K.A. (2014). Dietary supplementation with soy isoflavones or replacement with soy proteins prevents hepatic lipid droplet accumulation and alters expression of genes involved in lipid metabolism in rats. Genes Nutr..

[115] Ahmed M. (2015). Non-alcoholic fatty liver disease in 2015. World J. Hepatol..

[116] Liu H., Zhong H., Leng L., Jiang Z. (2017). Effects of soy isoflavone on hepatic steatosis in high fat-induced rats. J. Clin. Biochem. Nutr..

[117] Zhao L., Wang Y., Liu J., Wang K., Guo X., Ji B., Wu W., Zhou F. (2016). Protective effects of genistein and puerarin against chronic alcohol-induced liver injury in mice via antioxidant, anti-inflammatory, and anti-apoptotic mechanisms. J. Agric. Food Chem..

[118] Emami N.K., Jung U., Voy B., Dridi S. (2020). Radical response: Effects of heat stress-induced oxidative stress on lipid metabolism in the avian liver. Antioxidants.

[119] Yoo N.Y., Jeon S., Nam Y., Park Y.J., Won S.B., Kwon Y.H. (2015). Dietary supplementation of genistein alleviates liver inflammation and fibrosis mediated by a methionine-choline-deficient diet in db/db mice. J. Agric. Food Chem..

[120] Jing Z., Wei-Jie Y. (2016). Effects of soy protein containing isoflavones in patients with chronic kidney disease: A systematic review and meta-analysis. Clin. Nutr..

[121] Ranich T., Bhathena S.J., Velasquez M.T. (2001). Protective effects of dietary phytoestrogens in chronic renal disease. J. Ren. Nutr..

[122] Velasquez M.T., Bhathena S.J. (2001). Dietary phytoestrogens: A possible role in renal disease protection. Am. J. Kidney Dis..

[123] Li W.F., Yang K., Zhu P., Zhao H.Q., Song Y.H., Liu K.C., Huang W.F. (2017). Genistein ameliorates ischemia/reperfusion-induced renal injury in a SIRT1-dependent manner. Nutrients.

[124] Liu Z.M., Ho S.C., Chen Y.M., Tang N., Woo J. (2014). Effect of whole soy and purified isoflavone daidzein on renal function—A 6-month randomized controlled trial in equol-producing postmenopausal women with prehypertension. Clin. Biochem..

[125] Jheng H.F., Hayashi K., Matsumura Y., Kawada T., Seno S., Matsuda H., Inoue K., Nomura W., Takahashi H., Goto T. (2020). Anti-inflammatory and antioxidative properties of isoflavones provide renal protective effects distinct from those of dietary soy proteins against diabetic nephropathy. Mol. Nutr. Food Res..

[126] Liu C.L., Yan L., Cai K.R., Sun K., Qi Y., Han Y.L., Zhang X.D., Sun X.D. (2018). Effects of soybean isoflavones on Wnt/beta-catenin and the TGF-beta1 signaling pathway in renal tissue of type 2 diabetic rats. J. Biol. Regul. Homeost. Agents.

[127] Kim M.J., Lim Y. (2013). Protective effect of short-term genistein supplementation on the early stage in diabetes-induced renal damage. Mediators Inflamm..

[128] Schreihofer D.A., Oppong-Gyebi A. (2019). Genistein: Mechanisms of action for a pleiotropic neuroprotective agent in stroke. Nutr. Neurosci..

[129] Li Y., Chen F., Wei A., Bi F., Zhu X., Yin S., Lin W., Cao W. (2019). Klotho recovery by genistein via promoter histone acetylation and DNA demethylation mitigates renal fibrosis in mice. J. Mol. Med..

[130] Hardy T.M., Tollefsbol T.O. (2011). Epigenetic diet: Impact on the epigenome and cancer. Epigenomics.

[131] Bernatoniene J., Kazlauskaite J.A., Kopustinskiene D.M. (2021). Pleiotropic effects of isoflavones in inflammation and chronic degenerative diseases. Int. J. Mol. Sci..

[132] Basson A.R., Ahmed S., Almutairi R., Seo B., Cominelli F. (2021). Regulation of intestinal inflammation by soybean and soy-derived compounds. Foods.

[133] Hariri M., Ghasemi A., Baradaran H.R., Mollanoroozy E., Gholami A. (2021). Beneficial effect of soy isoflavones and soy isoflavones plus soy protein on serum concentration of C-reactive protein among postmenopausal women: An updated systematic review and meta-analysis of randomized controlled trials. Complement Ther. Med..

[134] Jubaidi F.F., Zainalabidin S., Taib I.S., Hamid Z.A., Budin S.B. (2021). The potential role of flavonoids in ameliorating diabetic cardiomyopathy via alleviation of cardiac oxidative stress, inflammation and apoptosis. Int. J. Mol. Sci..

[135] Tan B.L., Norhaizan M.E., Liew W.P. (2018). Nutrients and oxidative stress: Friend or foe?. Oxid. Med. Cell. Longev..

[136] Xu D.P., Li Y., Meng X., Zhou T., Zhou Y., Zheng J., Zhang J.J., Li H.B. (2017). Natural antioxidants in foods and medicinal plants: Extraction, assessment and resources. Int. J. Mol. Sci..

[137] Abernathy L.M., Fountain M.D., Rothstein S.E., David J.M., Yunker C.K., Rakowski J., Lonardo F., Joiner M.C., Hillman G.G. (2015). Soy isoflavones promote radioprotection of normal lung tissue by inhibition of radiation-induced activation of macrophages and neutrophils. J. Thorac. Oncol..

[138] Abernathy L.M., Fountain M.D., Joiner M.C., Hillman G.G. (2017). Innate immune pathways associated with lung radioprotection by soy isoflavones. Front. Oncol..

[139] Feng G., Sun B., Li T.Z. (2015). Daidzein attenuates lipopolysaccharide-induced acute lung injury via toll-like receptor 4/NF-kappaB pathway. Int. Immunopharmacol..

[140] Liu X.J., Bao H.R., Zeng X.L., Wei J.M. (2016). Effects of resveratrol and genistein on nuclear factor kappaB, tumor necrosis factor alpha and matrix metalloproteinase 9 in patients with chronic obstructive pulmonary disease. Mol. Med. Rep..

[141] Parida S., Singh T.U., Thangamalai R., Narasimha Reddy C.E., Panigrahi M., Kandasamy K., Singh V., Mishra S.K. (2015). Daidzein pretreatment improves survival in mouse model of sepsis. J. Surg. Res..

[142] Prasad K. (1999). Homocysteine, a risk factor for cardiovascular disease. Int. J. Angiol..

[143] Han S., Wu H., Li W., Gao P. (2015). Protective effects of genistein in homocysteine-induced endothelial cell inflammatory injury. Mol. Cell. Biochem..

[144] Susutlertpanya W., Werawatganon D., Siriviriyakul P., Klaikeaw N. (2015). Genistein attenuates nonalcoholic steatohepatitis and increases hepatic PPARγ in a rat model. Evid. Based. Complement. Alternat. Med..

[145] Ganai A.A., Khan A.A., Malik Z.A., Farooqi H. (2015). Genistein modulates the expression of NF-κB and MAPK (p-38 and ERK1/2), thereby attenuating d-Galactosamine induced fulminant hepatic failure in Wistar rats. Toxicol. Appl. Pharmacol..

[146] Wang B., Wu C. (2017). Dietary soy isoflavones alleviate dextran sulfate sodium-induced inflammation and oxidative stress in mice. Exp. Ther. Med..

[147] Li Y., Kong D., Bao B., Ahmad A., Sarkar F.H. (2011). Induction of cancer cell death by isoflavone: The role of multiple signaling pathways. Nutrients.

[148] Diana T., Brown R.S., Bossowski A., Segni M., Niedziela M., Konig J., Bossowska A., Ziora K., Hale A., Smith J. (2014). Clinical relevance of thyroid-stimulating autoantibodies in pediatric graves’ disease-a multicenter study. J. Clin. Endocrinol. Metab..

[149] Anderson L.N., Cotterchio M., Boucher B.A., Kreiger N. (2013). Phytoestrogen intake from foods, during adolescence and adulthood, and risk of breast cancer by estrogen and progesterone receptor tumor subgroup among Ontario women. Int. J. Cancer..

[150] Wang G., Zhang D., Yang S., Wang Y., Tang Z., Fu X. (2018). Co-administration of genistein with doxorubicin-loaded polypeptide nanoparticles weakens the metastasis of malignant prostate cancer by amplifying oxidative damage. Biomater. Sci..

[151] Applegate C.C., Rowles J.L., Ranard K.M., Jeon S., Erdman J.W. (2018). Soy consumption and the risk of prostate cancer: An updated systematic review and meta-analysis. Nutrients.

[152] Wong W.C., Wong E.L., Li H., You J.H., Ho S., Woo J., Hui E. (2012). Isoflavones in treating watchful waiting benign prostate hyperplasia: A double-blinded, randomized controlled trial. J. Altern. Complement. Med..

[153] Chen F.P., Chien M.H. (2014). Phytoestrogens induce apoptosis through a mitochondria/caspase pathway in human breast cancer cells. Climacteric.

[154] Zhang X., Cook K.L., Warri A., Cruz I.M., Rosim M., Riskin J., Helferich W., Doerge D., Clarke R., Hilakivi-Clarke L. (2017). Lifetime genistein intake increases the response of mammary tumors to Tamoxifen in tats. Clin. Cancer Res..

[155] Zhang F.F., Haslam D.E., Terry M.B., Knight J.A., Andrulis I.L., Daly M.B., Buys S.S., John E.M. (2017). Dietary isoflavone intake and all-cause mortality in breast cancer survivors: The Breast Cancer Family Registry. Cancer.

[156] Yu D., Shin H.S., Lee Y.S., Lee D., Kim S., Lee Y.C. (2014). Genistein attenuates cancer stem cell characteristics in gastric cancer through the downregulation of Gli1. Oncol. Rep..

[157] Fang Y., Zhang Q., Wang X., Wang X., Huang Z., Jiao Y., Wang J. (2016). Quantitiative phosphoproteomics reveals genistein as a modulator of cell cycle and DNA damage response pathways in triple-negative breast cancer cells. Int. J. Oncol..

[158] Lee J.Y., Kim H.S., Song Y.S. (2012). Genistein as a potential anticancer agent against ovarian cancer. J. Tradit. Complement. Med..

[159] Roh T., Kim S.W., Moon S.H., Nam M.J. (2016). Genistein induces apoptosis by down-regulating thioredoxin-1 in human hepatocellular carcinoma SNU-449 cells. Food Chem. Toxicol..

[160] Yang Y.M., Zang A., Jia Y., Shang Y., Zhang Z., Ge K., Zhang J., Fan W., Wang B. (2016). Genistein inhibits A549 human lung cell proliferation via miR-27a and MET signaling. Oncol. Lett..

[161] Tian T., Li J., Li B., Wang Y., Li M., Ma D., Wang X. (2014). Genistein exhibits anti-cancer effects via down-regulating FoxM1 in H446 small-cell lung cancer cells. Tumour Biol..

[162] Jaudan A., Sharma S., Malek S.N.A., Dixit A. (2018). Induction of apoptosis by pinostrobin in human cervical cancer cells: Possible mechanism of action. PLoS ONE.

[163] Chaitanya G.V., Steven A.J., Babu P.P. (2010). PARP-1 cleavage fragments: Signatures of cell-death proteases in neurodegeneration. Cell Commun. Signal..

[164] Martucciello S., Masullo M., Cerulli A., Piacente S. (2020). Natural products targeting ER stress, and the functional link to mitochondria. Int. J. Mol. Sci..

[165] Tranche S., Brotons C., Pascual de la Pisa B., Macias R., Hevia E., Marzo-Castillejo M. (2016). Impact of a soy drink on climacteric symptoms: An open-label, crossover, randomized clinical trial. Gynecol. Endocrinol..

[166] Ghaemi A., Soleimanjahi H., Razeghi S., Gorji A., Tabaraei A., Wada K., Tsuji M., Tamura T., Konishi K., Kawachi T. (2015). Soy isoflavone intake and stomach cancer risk in Japan: From the Takayama study. Int. J. Cancer.

[167] Moradi A., Alizadeh A., Vakili M.A. (2012). Genistein induces a protective immunomodulatory effect in a mouse model of cervical cancer. Iran J. Immunol..

[168] Liu Z.M., Ho S., Hao Y.T., Chen Y.M., Woo J., Wong S.Y., He Q., Xie Y.J., Tse L.A., Chen B. (2016). Randomised controlled trial of effect of whole soy replacement diet on features of metabolic syndrome in postmenopausal women: Study protocol. BMJ Open.

[169] Yu Y., Jing X., Li H., Zhao X., Wang D. (2016). Soy isoflavone consumption and colorectal cancer risk: A systematic review and meta-analysis. Sci. Rep..

[170] Shin A., Lee J., Lee J., Park M.S., Park J.W., Park S.C., Oh J.H., Kim J. (2015). Isoflavone and soyfood intake and colorectal cancer risk: A case-control study in Korea. PLoS ONE.

[171] Lee A.H., Su D., Pasalich M., Tang L., Binns C.W., Qiu L. (2014). Soy and isoflavone intake associated with reduced risk of ovarian cancer in southern Chinese women. Nutr. Res..

[172] Kim Y.S., Choi K.C., Hwang K.A. (2015). Genistein suppressed epithelial-mesenchymal transition and migration efficacies of BG-1 ovarian cancer cells activated by estrogenic chemicals via estrogen receptor pathway and downregulation of TGF-β signaling pathway. Phytomedicine.

[173] Lazarevic B., Hammarstrom C., Yang J., Ramberg H., Diep L.M., Karlsen S.J., Kucuk O., Saatcioglu F., Tasken K.A., Svindland A. (2012). The effects of short-term genistein intervention on prostate biomarker expression in patients with localised prostate cancer before radical prostatectomy. Br. J. Nutr..

[174] Chiyomaru T., Yamamura S., Fukuhara S., Yoshino H., Kinoshita T., Majid S., Saini S., Chang I., Tanaka Y., Enokida H. (2013). Genistein inhibits prostate cancer cell growth by targeting miR-34a and oncogenic HOTAIR. PLoS ONE.

[175] Bilir B., Sharma N.V., Lee J., Hammarstrom B., Svindland A., Kucuk O., Moreno C.S. (2017). Effects of genistein supplementation on genomewide DNA methylation and gene expression in patients with localized prostate cancer. Int. J. Oncol..

[176] Mayo B., Vazquez L., Florez A.B. (2019). Equol: A bacterial metabolite from the daidzein isoflavone and its presumed beneficial health effects. Nutrients.

[177] Lu Z., Zhou R., Kong Y., Wang J., Xia W., Guo J., Liu J., Sun H., Liu K., Yang J. (2016). *S*-equol, a secondary metabolite of natural anticancer isoflavone daidzein, inhibits prostate cancer growth in vitro and in vivo, though activating the Akt/FOXO3a pathway. Curr. Cancer Drug Targets.

[178] Lesinski G.B., Reville P.K., Mace T.A., Young G.S., Ahn-Jarvis J., Thomas-Ahner J., Vodovotz Y., Ameen Z., Grainger E., Riedl K. (2015). Consumption of soy isoflavone enriched bread in men with prostate cancer is associated with reduced proinflammatory cytokines and immunosuppressive cells. Cancer Prev. Res..

[179] Wu Y., Zhang L., Na R., Xu J., Xiong Z., Zhang N., Dai W., Jiang H., Ding Q. (2015). Plasma genistein and risk of prostate cancer in Chinese population. Int. Urol. Nephrol..

[180] Dong X., Xu W., Sikes R.A., Wu C. (2013). Combination of low dose of genistein and daidzein has synergistic preventive effects on isogenic human prostate cancer cells when compared with individual soy isoflavone. Food Chem..

[181] Yang L., Shi P., Zhao G., Xu J., Peng W., Zhang J., Zhang G., Wang X., Dong Z., Chen F. (2020). Targeting cancer stem cell pathways for cancer therapy. Signal Transduct. Target Ther..

[182] Li E., Zhang T., Sun X., Li Y., Geng H., Yu D., Zhong C. (2019). Sonic hedgehog pathway mediates genistein inhibition of renal cancer stem cells. Oncol. Lett..

[183] Fritz H., Seely D., Flower G., Skidmore B., Fernandes R., Vadeboncoeur S., Kennedy D., Cooley K., Wong R., Sagar S. (2013). Soy, red clover, and isoflavones and breast cancer: A systematic review. PLoS ONE.

[184] Xu J., Xiong H., Zhao Z., Luo M., Ju Y., Yang G., Mei Z. (2021). Genistein suppresses allergic contact dermatitis through regulating the MAP2K2/ERK pathway. Food Funct..

[185] Molina L., Bustamante F.A., Bhoola K.D., Figueroa C.D., Ehrenfeld P. (2018). Possible role of phytoestrogens in breast cancer via GPER-1/GPR30 signaling. Clin. Sci..

[186] Messina M. (2016). Impact of soy foods on the development of breast cancer and the prognosis of breast cancer patients. Forsch. Komplementmed..

[187] Betancourt A.M., Wang J., Jenkins S., Mobley J., Russo J., Lamartiniere C.A. (2012). Altered carcinogenesis and proteome in mammary glands of rats after prepubertal exposures to the hormonally active chemicals bisphenol A and genistein. J. Nutr..

[188] Van Duursen M.B., Nijmeijer S.M., de Morree E.S., de Jong P.C., van den Berg M. (2011). Genistein induces breast cancer-associated aromatase and stimulates estrogen-dependent tumor cell growth in *in vitro* breast cancer model. Toxicology.

[189] Allred C.D., Allred K.F., Ju Y.H., Goeppinger T.S., Doerge D.R., Helferich W.G. (2004). Soy processing influences growth of estrogen-dependent breast cancer tumors. Carcinogenesis.

[190] Allred C.D., Ju Y.H., Allred K.F., Chang J., Helferich W.G. (2001). Dietary genistin stimulates growth of estrogen-dependent breast cancer tumors similar to that observed with genistein. Carcinogenesis.

[191] Poschner S., Maier-Salamon A., Zehl M., Wackerlig J., Dobusch D., Pachmann B., Sterlini K.L., Jager W. (2017). The impacts of genistein and daidzein on estrogen conjugations in human breast cancer cells: A targeted metabolomics approach. Front. Pharmacol..

[192] Jurkiewicz-Przondziono J., Lemm M., Kwiatkowska-Pamula A., Ziolko E., Wojtowicz M.K. (2017). Influence of diet on the risk of developing endometriosis. Ginekol. Pol..

[193] Mvondo M.A., Ekenfack J.D., Minko Essono S., Saah Namekong H., Awounfack C.F., Laschke M.W., Njamen D. (2019). Soy intake since the prepubertal age may contribute to the pathogenesis of endometriosis in adulthood. J. Med. Food.

[194] Noel J.C., Anaf V., Fayt I., Wespes E. (2006). Ureteral mullerian carcinosarcoma (mixed mullerian tumor) associated with endometriosis occurring in a patient with a concentrated soy isoflavones supplementation. Arch. Gynecol. Obstet..

[195] Sea J.L., Abramyan M., Chiu H.K. (2021). Prepubescent unilateral gynecomastia secondary to excessive soy consumption. J. Pediatr. Endocrinol. Metab..

[196] Martinez J., Lewi J.E. (2008). An unusual case of gynecomastia associated with soy product consumption. Endocr. Pract..

[197] Ranjan A., Ramachandran S., Gupta N., Kaushik I., Wright S., Srivastava S., Das H., Srivastava S., Prasad S., Srivastava S.K. (2019). Role of phytochemicals in cancer prevention. Int. J. Mol. Sci..

[198] Ziaei S., Halaby R. (2017). Dietary isoflavones and breast cancer risk. Medicines.

[199] Kucuk O. (2017). Soy foods, isoflavones, and breast cancer. Cancer.

[200] Kanadys W., Baranska A., Blaszczuk A., Polz-Dacewicz M., Drop B., Malm M., Kanecki K. (2021). Effects of soy isoflavones on biochemical markers of bone metabolism in postmenopausal women: A systematic review and meta-analysis of randomized controlled trials. Int. J. Environ. Res. Public Health.

[201] Alswat K.A. (2017). Gender disparities in osteoporosis. J. Clin. Med. Res..

[202] Ji M.X., Yu Q. (2015). Primary osteoporosis in postmenopausal women. Chronic. Dis. Transl. Med..

[203] Ho S.C., Wong E., Chan S.G., Lau J., Chan C., Leung P.C. (1997). Determinants of peak bone mass in Chinese women aged 21-40 years. III. Physical activity and bone mineral density. J. Bone Miner. Res..

[204] Gordon C.M., Zemel B.S., Wren T.A., Leonard M.B., Bachrach L.K., Rauch F., Gilsanz V., Rosen C.J., Winer K.K. (2017). The determinants of peak bone mass. J. Pediatr..

[205] Noh D., Lim Y., Lee H., Kim H., Kwon O. (2018). Soybean-Hop alleviates estrogen deficiency-related bone loss and metabolic dysfunction in ovariectomized rats fed a high-fat diet. Molecules.

[206] Yu F., Liu Z., Tong Z., Zhao Z., Liang H. (2015). Soybean isoflavone treatment induces osteoblast differentiation and proliferation by regulating analysis of Wnt/beta-catenin pathway. Gene.

[207] Ahn H., Park Y.K. (2017). Soy isoflavone supplementation improves longitudinal bone growth and bone quality in growing female rats. Nutrition.

[208] Santos M.A., Florencio-Silva R., Medeiros V.P., Nader H.B., Nonaka K.O., Sasso G.R., Simoes M.J., Reginato R.D. (2014). Effects of different doses of soy isoflavones on bone tissue of ovariectomized rats. Climacteric.

[209] Zheng X., Lee S.K., Chun O.K. (2016). Soy Isoflavones and osteoporotic bone loss: A review with an emphasis on modulation of bone remodeling. J. Med. Food.

[210] Ward W.E., Kaludjerovic J., Dinsdale E.C. (2016). A mouse model for studying nutritional programming: Effects of early life exposure to soy isoflavones on bone and reproductive health. Int. J. Environ. Res. Public Health.

[211] Kim S.M., Lee H.S., Jung J.I., Lim S.M., Lim J.H., Ha W.H., Jeon C.L., Lee J.Y., Kim E.J. (2018). Effect of isoflavone-enriched whole soy milk powder supplementation on bone metabolism in ovariectomized mice. Nutr. Res. Pract..

[212] Al-Ishaq R.K., Abotaleb M., Kubatka P., Kajo K., Busselberg D. (2019). Flavonoids and their anti-diabetic effects: Cellular mechanisms and effects to improve blood sugar levels. Biomolecules.

[213] Weng L., Zhang F., Wang R., Ma W., Song Y. (2019). A review on protective role of genistein against oxidative stress in diabetes and related complications. Chem. Biol. Interact..

[214] Maliehe A., Ghahremani S., Kharghani S., Ghazanfarpour M., Shariati K., Kazemi M., Khadivzadeh T. (2019). Effect of isoflavones and genistein on glucose metabolism in peri- and post-menopausal women: An overview of meta-analysis. J. Menopausal Med..

[215] Chen X., Yu J., Shi J. (2018). Management of diabetes mellitus with puerarin, a natural isoflavone from *Pueraria lobata*. Am. J. Chin. Med..

[216] Glisic M., Kastrati N., Gonzalez-Jaramillo V., Bramer W.M., Ahmadizar F., Chowdhury R., Danser A.J., Roks A.J., Voortman T., Franco O.H. (2018). Associations between phytoestrogens, glucose homeostasis, and risk of diabetes in women: A systematic review and meta-analysis. Adv. Nutr..

[217] Cheng S.Y., Shaw N.S., Tsai K.S., Chen C.Y. (2004). The hypoglycemic effects of soy isoflavones on postmenopausal women. J. Womens Health.

[218] Gilbert E.R., Liu D. (2013). Anti-diabetic functions of soy isoflavone genistein: Mechanisms underlying its effects on pancreatic beta-cell function. Food Funct..

[219] Zhang Y.B., Chen W.H., Guo J.J., Fu Z.H., Yi C., Zhang M., Na X.L. (2013). Soy isoflavone supplementation could reduce body weight and improve glucose metabolism in non-Asian postmenopausal women—A meta-analysis. Nutrition.

[220] Jamilian M., Asemi Z. (2016). The effects of soy isoflavones on metabolic status of patients with polycystic ovary syndrome. J. Clin. Endocrinol. Metab..

[221] Guo T.L., Germolec D.R., Zheng J.F., Kooistra L., Auttachoat W., Smith M.J., White K.L., Elmore S.A. (2015). Genistein protects female nonobese diabetic mice from developing type 1 diabetes when fed a soy- and alfalfa-free diet. Toxicol. Pathol..

[222] Sharma B., Mittal A., Dabur R. (2018). Mechanistic approach of anti-diabetic compounds identified from natural sources. Chem. Biol. Lett..

[223] Cheong S.H., Furuhashi K., Ito K., Nagaoka M., Yonezawa T., Miura Y., Yagasaki K. (2014). Antihyperglycemic effect of equol, a daidzein derivative, in cultured L6 myocytes and ob/ob mice. Mol. Nutr. Food Res..

[224] Li G., Prior J.C., Leslie W.D., Thabane L., Papaioannou A., Josse R.G., Kaiser S.M., Kovacs C.S., Anastassiades T., Towheed T. (2019). Frailty and risk of fractures in patients with type 2 diabetes. Diabetes Care.

[225] Sathyapalan T., Aye M., Rigby A.S., Fraser W.D., Kilpatrick E.S., Atkin S.L. (2017). Effect of soy on bone turn-over markers in men with type 2 diabetes and hypogonadism—A randomised controlled study. Sci. Rep..

[226] Ding M., Franke A.A., Rosner B.A., Giovannucci E., van Dam R.M., Tworoger S.S., Hu F.B., Sun Q. (2015). Urinary isoflavonoids and risk of type 2 diabetes: A prospective investigation in US women. Br. J. Nutr..

[227] Ding M., Pan A., Manson J.E., Willett W.C., Malik V., Rosner B., Giovannucci E., Hu F.B., Sun Q. (2016). Consumption of soy foods and isoflavones and risk of type 2 diabetes: A pooled analysis of three US cohorts. Eur. J. Clin. Nutr..

[228] Jin M., Shen M.H., Jin M.H., Jin A.H., Yin X.Z., Quan J.S. (2018). Hypoglycemic property of soy isoflavones from hypocotyl in Goto-Kakizaki diabetic rats. J. Clin. Biochem. Nutr..

[229] Zhang C., Gao F., Luo H., Zhang C.T., Zhang R. (2015). Differential response in levels of high-density lipoprotein cholesterol to one-year metformin treatment in prediabetic patients by race/ethnicity. Cardiovasc. Diabetol..

[230] Horiuchi H., Usami A., Shirai R., Harada N., Ikushiro S., Sakaki T., Nakano Y., Inui H., Yamaji R. (2017). *S*-equol activates cAMP signaling at the plasma membrane of INS-1 pancreatic β-cells and protects against streptozotocin-induced hyperglycemia by increasing β-Cell function in male mice. J. Nutr..

[231] Zebrowitz L.A. (2017). First impressions from faces. Curr. Dir. Psychol. Sci..

[232] Bochenska K., Gabig-Ciminska M. (2020). Unbalanced sphingolipid metabolism and its implications for the pathogenesis of psoriasis. Molecules.

[233] Blair M.J., Jones J.D., Woessner A.E., Quinn K.P. (2020). Skin structure-function relationships and the wound healing response to intrinsic aging. Adv. Wound Care.

[234] Kim H., Park S.Y., Lee G. (2019). Potential therapeutic applications of bee venom on skin disease and its mechanisms: A literature review. Toxins.

[235] Lee T.H., Do M.H., Oh Y.L., Cho D.W., Kim S.H., Kim S.Y. (2014). Dietary fermented soybean suppresses UVB-induced skin inflammation in hairless mice via regulation of the MAPK signaling pathway. J. Agric. Food Chem..

[236] Iovine B., Iannella M.L., Gasparri F., Giannini V., Monfrecola G., Bevilacqua M.A. (2012). A comparative analysis of the photo-protective effects of soy isoflavones in their aglycone and glucoside forms. Int. J. Mol. Sci..

[237] Liu T., Li N., Yan Y.Q., Liu Y., Xiong K., Liu Y., Xia Q.M., Zhang H., Liu Z.D. (2020). Recent advances in the anti-aging effects of phytoestrogens on collagen, water content, and oxidative stress. Phytother. Res..

[238] Park E., Lee S.M., Jung I.K., Lim Y., Kim J.H. (2011). Effects of genistein on early-stage cutaneous wound healing. Biochem. Biophys. Res. Commun..

[239] Kim Y.M., Huh J.S., Lim Y., Cho M. (2015). Soy isoflavone glycitin (4′-hydroxy-6-methoxyisoflavone-7-D-glucoside) promotes human dermal fibroblast cell proliferation and migration via TGF-β signaling. Phytother. Res..

[240] Lim T.G., Kim J.E., Lee S.Y., Park J.S., Yeom M.H., Chen H., Bode A.M., Dong Z., Lee K.W. (2014). The daidzein metabolite, 6,7,4′-trihydroxyisoflavone, is a novel inhibitor of PKCα in suppressing solar UV-induced matrix metalloproteinase 1. Int. J. Mol. Sci..

[241] Mason A., Kinugasa T. (2008). East Asian economic development: Two demographic dividends. J. Asian Econ..

[242] Shin S., Lee J.A., Son D., Park D., Jung E. (2017). Anti-skin-aging activity of a standardized extract from *Panax ginseng* leaves in vitro and in human volunteer. Cosmetics.

[243] Jenkins G., Wainwright L.J., Holland R., Barrett K.E., Casey J. (2014). Wrinkle reduction in post-menopausal women consuming a novel oral supplement: A double-blind placebo-controlled randomized study. Int. J. Cosmet. Sci..

[244] Faber L., Kovac I., Mitrengova P., Novotny M., Varinska L., Vasilenko T., Kello M., Coma M., Kuruc T., Petrova K. (2018). Genistein improves skin flap viability in rats: A preliminary in vivo and in vitro investigation. Molecules.

[245] Li H.J., Wu N.L., Lee G.A., Hung C.F. (2018). The therapeutic potential and molecular mechanism of isoflavone extract against psoriasis. Sci. Rep..

[246] Back P.I., Furtado L.R., Nemitz M.C., Balestrin L.A., Fachel F.N.S., Gomes H.M., Schuh R.S., Moreira J.C., von Poser G.L., Teixeira H.F. (2018). Skin permeation and oxidative protection effect of soybean isoflavones from topical nanoemulsions-a comparative study of extracts and pure compounds. AAPS PharmSciTech.

[247] Savoia P., Raina G., Camillo L., Farruggio S., Mary D., Veronese F., Graziola F., Zavattaro E., Tiberio R., Grossini E. (2018). Anti-oxidative effects of 17 β-estradiol and genistein in human skin fibroblasts and keratinocytes. J. Dermatol. Sci..

[248] Duchnik E., Kruk J., Baranowska-Bosiacka I., Pilutin A., Maleszka R., Marchlewicz M. (2019). Effects of the soy isoflavones, genistein and daidzein, on male rats’ skin. Postepy Dermatol. Alergol..

[249] Wang A., Wei J., Lu C., Chen H., Zhong X., Lu Y., Li L., Huang H., Dai Z., Han L. (2019). Genistein suppresses psoriasis-related inflammation through a STAT3-NF-κB-dependent mechanism in keratinocytes. Int. Immunopharmacol..

[250] Sansai K., Na Takuathung M., Khatsri R., Teekachunhatean S., Hanprasertpong N., Koonrungsesomboon N. (2020). Effects of isoflavone interventions on bone mineral density in postmenopausal women: A systematic review and meta-analysis of randomized controlled trials. Osteoporos. Int..

[251] Qiu S., Ma Y., Jiang C. (2020). Isoflavone combined with exercise on bone mineral density in postmenopausal women: A systematic review and meta-analysis of randomized controlled trials. J. Chin. Med. Assoc..

[252] Lambert M.N.T., Hu L.M., Jeppesen P.B. (2017). A systematic review and meta-analysis of the effects of isoflavone formulations against estrogen-deficient bone resorption in peri- and postmenopausal women. Am. J. Clin. Nutr..

[253] Sankar P., Zachariah B., Anissa A.M. (2017). Soy isoflavones (from *Glycine max*) in menopause health and diseases. Biochem.Physiol..

[254] Chilibeck P.D., Vatanparast H., Pierson R., Case A., Olatunbosun O., Whiting S.J., Beck T.J., Pahwa P., Biem H.J. (2013). Effect of exercise training combined with isoflavone supplementation on bone and lipids in postmenopausal women: A randomized clinical trial. J. Bone Miner. Res..

[255] Liu Z.M., Ho S.C., Chen Y.M., Woo J. (2013). Effect of soy protein and isoflavones on blood pressure and endothelial cytokines: A 6-month randomized controlled trial among postmenopausal women. J. Hypertens..

[256] Xiao Y., Zhang S., Tong H., Shi S. (2018). Comprehensive evaluation of the role of soy and isoflavone supplementation in humans and animals over the past two decades. Phytother. Res..

[257] Ahsan M., Mallick A.K. (2017). The effect of soy isoflavones on the menopause rating scale scoring in perimenopausal and postmenopausal women: A pilot study. J. Clin. Diagn. Res..

[258] De Franciscis P., Grauso F., Luisi A., Schettino M.T., Torella M., Colacurci N. (2017). Adding agnus castus and magnolia to soy isoflavones relieves sleep disturbances besides postmenopausal vasomotor symptoms-long term safety and effectiveness. Nutrients.

[259] Yang D., Yang S., Zhang B., Lu Y. (2016). Certification of reference materials for analysis of isoflavones genistin and genistein in soy products. Anal. Methods.

[260] Atteritano M., Mazzaferro S., Bitto A., Cannata M.L., D’Anna R., Squadrito F., Macri I., Frisina A., Frisina N., Bagnato G. (2014). Genistein effects on quality of life and depression symptoms in osteopenic postmenopausal women: A 2-year randomized, double-blind, controlled study. Osteoporos. Int..

[261] Furlong O., Parr H., Hodge S.J., Slevin M.M., Simpson E.E., McSorley E.M., McCormack J.M., Magee P.J. (2020). Consumption of soy drink has no drink on cognitive function but may alleviate vasomotor symptoms in post-menopausal women—A randomised trial. Eur. J. Nutr..

[262] Allahdadi K.J., Tostes R.C., Webb R.C. (2009). Female sexual dysfunction: Therapeutic options and experimental challenges. Cardiovasc. Hematol. Agents Med. Chem..

[263] Basson R., Gilks T. (2018). Women’s sexual dysfunction associated with psychiatric disorders and their treatment. Womens Health.

[264] Jaafarpour M., Khani A., Khajavikhan J., Suhrabi Z. (2013). Female sexual dysfunction: Prevalence and risk factors. J. Clin. Diagn. Res..

[265] Zhang J., Zhu Y., Pan L., Xia H., Ma J., Zhang A. (2019). Soy isoflavone improved female sexual dysfunction of mice via endothelial nitric oxide synthase pathway. Sex. Med..

[266] Daily J.W., Ko B.S., Ryuk J., Liu M., Zhang W., Park S. (2019). Equol decreases hot flashes in postmenopausal women: A systematic review and meta-analysis of randomized clinical trials. J. Med. Food.

[267] Hachul H., Brandao L.C., D’Almeida V., Bittencourt L.R., Baracat E.C., Tufik S. (2011). Isoflavones decrease insomnia in postmenopause. Menopause.

[268] Burt Solorzano C.M., McCartney C.R. (2010). Obesity and the pubertal transition in girls and boys. Reproduction.

[269] Desmawati D., Sulastri D. (2019). A phytoestrogens and their health effect. Open Access Maced. J. Med. Sci..

[270] Kim S.H., Park M.J. (2012). Effects of phytoestrogen on sexual development. Korean J. Pediatr..

[271] Patisaul H.B., Jefferson W. (2010). The pros and cons of phytoestrogens. Front. Neuroendocrinol..

[272] Macon M.B., Fenton S.E. (2013). Endocrine disruptors and the breast: Early life effects and later life disease. J. Mammary Gland Biol. Neoplasia.

[273] Yuan X.X., Zhang B., Li L.L., Xiao C.W., Fan J.X., Geng M.M., Yin Y.L. (2012). Effects of soybean isoflavones on reproductive parameters in Chinese mini-pig boars. J. Anim. Sci. Biotechnol..

[274] Woclawek-Potocka I., Mannelli C., Boruszewska D., Kowalczyk-Zieba I., Wasniewski T., Skarzynski D.J. (2013). Diverse effects of phytoestrogens on the reproductive performance: Cow as a model. Int. J. Endocrinol..

[275] Dinsdale E.C., Ward W.E. (2010). Early exposure to soy isoflavones and effects on reproductive health: A review of human and animal studies. Nutrients.

[276] Sinai T., Ben-Avraham S., Guelmann-Mizrahi I., Goldberg M.R., Naugolni L., Askapa G., Katz Y., Rachmiel M. (2019). Consumption of soy-based infant formula is not associated with early onset of puberty. Eur. J. Nutr..

[277] Duitama S.M., Zurita J., Cordoba D., Duran P., Ilag L., Mejia W. (2018). Soy protein supplement intake for 12 months has no effect on sexual maturation and may improve nutritional status in pre-pubertal children. J. Paediatr. Child Health.

[278] Ronis M.J., Gomez-Acevedo H., Shankar K., Sharma N., Blackburn M., Singhal R., Mercer K.E., Badger T.M. (2018). EB 2017 Article: Soy protein isolate feeding does not result in reproductive toxicity in the pre-pubertal rat testis. Exp. Biol. Med..

[279] Bernardo B.D., Brandt J.Z., Grassi T.F., Silveira L.T., Scarano W.R., Barbisan L.F. (2015). Genistein reduces the noxious effects of in utero bisphenol A exposure on the rat prostate gland at weaning and in adulthood. Food Chem. Toxicol..

[280] Rizzo G., Baroni L. (2018). Soy, soy foods and their role in vegetarian diets. Nutrients.

[281] Messina M., Rogero M.M., Fisberg M., Waitzberg D. (2017). Health impact of childhood and adolescent soy consumption. Nutr. Rev..

[282] Miyake Y., Tanaka K., Okubo H., Sasaki S., Tokinobu A., Arakawa M. (2021). Maternal consumption of soy and isoflavones during pregnancy and risk of childhood behavioural problems: The Kyushu Okinawa Maternal and Child Health Study. Int. J. Food Sci. Nutr..

[283] Korde L.A., Wu A.H., Fears T., Nomura A.M., West D.W., Kolonel L.N., Pike M.C., Hoover R.N., Ziegler R.G. (2009). Childhood soy intake and breast cancer risk in Asian American women. Cancer Epidemiol. Biomarkers Prev..

[284] Upson K., Sathyanarayana S., Scholes D., Holt V.L. (2015). Early-life factors and endometriosis risk. Fertil. Steril..

[285] Gencel V.B., Benjamin M.M., Bahou S.N., Khalil R.A. (2012). Vascular effects of phytoestrogens and alternative menopausal hormone therapy in cardiovascular disease. Mini Rev. Med. Chem..

[286] Cavallini D.C., Manzoni M.S., Bedani R., Roselino M.N., Celiberto L.S., Vendramini R.C., de Valdez G., Abdalla D.S., Pinto R.A., Rosetto D. (2016). Probiotic soy product supplemented with isoflavones improves the lipid profile of moderately hypercholesterolemic men: A randomized controlled trial. Nutrients.

[287] Kladna A., Berczynski P., Kruk I., Piechowska T., Aboul-Enein H.Y. (2016). Studies on the antioxidant properties of some phytoestrogens. Luminescence.

[288] Rufer C.E., Kulling S.E. (2006). Antioxidant activity of isoflavones and their major metabolites using different in vitro assays. J. Agric. Food. Chem..

[289] Yoon G.A., Park S. (2014). Antioxidant action of soy isoflavones on oxidative stress and antioxidant enzyme activities in exercised rats. Nutr. Res. Pract..

[290] Tronina T., Poplonski J., Bartmanska A. (2020). Flavonoids as phytoestrogenic components of hops and beer. Molecules.

[291] Tava A., Stochmal A., Pecetti L. (2016). Isoflavone content in subterranean clover germplasm from Sardinia. Chem. Biodivers..

[292] Budryn G., Galazka-Czarnecka I., Brzozowska E., Grzelczyk J., Mostowski R., Zyzelewicz D., Ceron-Carrasco J.P., Perez-Sanchez H. (2018). Evaluation of estrogenic activity of red clover (*Trifolium pratense* L.) sprouts cultivated under different conditions by content of isoflavones, calorimetric study and molecular modelling. Food Chem..

[293] Borras C., Gambini J., Gomez-Cabrera M.C., Sastre J., Pallardo F.V., Mann G.E., Vina J. (2006). Genistein, a soy isoflavone, up-regulates expression of antioxidant genes: Involvement of estrogen receptors, ERK1/2, and NFkeppaB. FASEB J..

[294] Ma D., Zhang Y., Yang T., Xue Y., Wang P. (2014). Isoflavone intake inhibits the development of 7,12-dimethylbenz(a)anthracene(DMBA)-induced mammary tumors in normal and ovariectomized rats. J. Clin. Biochem. Nutr..

[295] Lee J.S. (2006). Effects of soy protein and genistein on blood glucose, antioxidant enzyme activities, and lipid profile in streptozotocin-induced diabetic rats. Life Sci..

[296] Valsecchi A.E., Franchi S., Panerai A.E., Rossi A., Sacerdote P., Colleoni M. (2011). The soy isoflavone genistein reverses oxidative and inflammatory state, neuropathic pain, neurotrophic and vasculature deficits in diabetes mouse model. Eur. J. Pharmacol..

[297] Kasapoglu M., Özben T. (2001). Alterations of antioxidant enzymes and oxidative stress markers in aging. Exp. Gerontol..

[298] Gou Z., Jiang S., Zheng C., Tian Z., Lin X. (2015). Equol inhibits LPS-induced oxidative stress and enhances the immune response in chicken HD11 macrophages. Cell. Physiol. Biochem..

[299] Qian K., Gao A.J., Zhu M.Y., Shao H.X., Jin W.J., Ye J.Q., Qin A.J. (2014). Genistein inhibits the replication of avian leucosis virus subgroup J in DF-1 cells. Virus Res..

[300] Andres A., Donovan S.M., Kuhlenschmidt M.S. (2009). Soy isoflavones and virus infections. J. Nutr. Biochem..

[301] Sithisarn P., Michaelis M., Schubert-Zsilavecz M., Cinatl Jr J. (2013). Differential antiviral and anti-inflammatory mechanisms of the flavonoids biochanin A and baicalein in H5N1 influenza A virus-infected cells. Antiviral Res..

[302] Miyake Y., Sasaki S., Ohya Y., Miyamoto S., Matsunaga I., Yoshida T., Hirota Y., Oda H. (2005). Soy, isoflavones, and prevalence of allergic rhinitis in Japanese women: The Osaka Maternal and Child Health Study. J. Allergy Clin. Immunol..

[303] Messina M.J., Wood C.E. (2008). Soy isoflavones, estrogen therapy, and breast cancer risk: Analysis and commentary. Nutr. J..

[304] Marini H., Polito F., Adamo E.B., Bitto A., Squadrito F., Benvenga S. (2012). Update on genistein and thyroid: An overall message of safety. Front. Endocrinol. (Lausanne).

[305] Benvenga S., Ducharme M., Yue C.S., Colucci P. (2010). A review of the pharmacokinetics of levothyroxine for the treatment of hypothyroidism. Eur. Endocrinol..

[306] Jonklaas J., Bianco A.C., Bauer A.J., Burman K.D., Cappola A.R., Celi F.S., Cooper D.S., Kim B.W., Peeters R.P., Rosenthal M.S. (2014). Guidelines for the treatment of hypothyroidism: Prepared by the American thyroid association task force on thyroid hormone replacement. Thyroid.

[307] Messina M., Mejia S.B., Cassidy A., Duncan A., Kurzer M., Nagato C., Ronis M., Rowland I., Sievenpiper J., Barnes S. (2021). Neither soyfoods nor isoflavones warrant classification as endocrine disruptors: A technical review of the observational and clinical data. Crit. Rev. Food Sci. Nutr..

[308] Setchell K.D.R. (2017). The history and basic science development of soy isoflavones. Menopause.

[309] Rachon D., Menche A., Vortherms T., Seidlova-Wuttke D., Wuttke W. (2008). Effects of dietary equol administration on the mammary gland in ovariectomized Sprague-Dawley rats. Menopause.

[310] Upson K., Adgent M.A., Wegienka G., Baird D.D. (2019). Soy-based infant formula feeding and menstrual pain in a cohort of women aged 23-35 years. Hum. Reprod..

[311] Tsuchiya M., Miura T., Hanaoka T., Iwasaki M., Sasaki H., Tanaka T., Nakao H., Katoh T., Ikenoue T., Kabuto M. (2007). Effect of soy isoflavones on endometriosis: Interaction with estrogen receptor 2 gene polymorphism. Epidemiology.

[312] Yamamoto A., Johnstone E.B., Bloom M.S., Huddleston H.G., Fujimoto V.Y. (2017). A higher prevalence of endometriosis among Asian women does not contribute to poorer IVF outcomes. J. Assist. Reprod. Genet..

[313] Zhang G.Q., Chen J.L., Liu Q., Zhang Y., Zeng H., Zhao Y. (2015). Soy intake is associated with lower endometrial cancer risk: A systematic review and meta-analysis of observational studies. Medicine.

[314] Murray M.J., Meyer W.R., Lessey B.A., Oi R.H., DeWire R.E., Fritz M.A. (2003). Soy protein isolate with isoflavones does not prevent estradiol-induced endometrial hyperplasia in postmenopausal women: A pilot trial. Menopause.

[315] Unfer V., Casini M.L., Costabile L., Mignosa M., Gerli S., Di Renzo G.C. (2004). High dose of phytoestrogens can reverse the antiestrogenic effects of clomiphene citrate on the endometrium in patients undergoing intrauterine insemination: A randomized trial. J. Soc. Gynecol. Investig..

[316] Unfer V., Casini M.L., Costabile L., Mignosa M., Gerli S., Di Renzo G.C. (2004). Endometrial effects of long-term treatment with phytoestrogens: A randomized, double-blind, placebo-controlled study. Fertil. Steril..

[317] Garg S., Lule V.K., Malik R.K., Tomar S.K. (2016). Soy bioactive components in functional perspective: A review. Int. J. Food Prop..

[318] Song W.O., Chun O.K., Hwang I., Shin H.S., Kim B.G., Kim K.S., Lee S.Y., Shin D., Lee S.G. (2007). Soy isoflavones as safe functional ingredients. J. Med. Food.

[319] Lee M.J., Kim J.H. (2007). Estimated dietary isoflavone intake among Korean adults. Nutr. Res. Pract..

