# Supplementary material for: Current Perspectives on the Beneficial Effects of Soybean Isoflavones and Their Metabolites for Humans
Source: Antioxidants (Basel). 2021 Jun 30;10(7):1064. doi: 10.3390/antiox10071064 (PMC8301030; doi:10.3390/antiox10071064)
Supplement: Supplementary file 1 [file antioxidants-10-01064-s001.zip › antioxidants-1275614-supplementary.pdf]

antioxidants

MDPI

Supplementary Materials: Current Perspectives on the Beneficial Effects of Soybean Isoflavones and Their Metabolites for Humans

Il-Sup Kim

Advanced Bio-Resource Research Center, Kyungpook National University, Daegu 41566, Korea; 92kis@hanmail.net

Table S1. Experimental design strategy.

Model	Isoflavone type	Approach	Assay	Ref
Neuroprotective effect and improvement of cognitive impairment				
Institute Cancer Research (ICR) mice	Isoflavones	*In vivo*	Observed	55
Virgin female Wistar rats	Genistein	*In vivo*	Observed	58
Pregnant Sprague–Dawley (SD) rats	Genistein	*In vivo*	Observed	61
Antidepressant effects				
Male ICR mice	Genistein	*In vivo*	Observed	66
Depressive pregnant women	Isoflavones	*In vivo*	Clinical	67
Anti-obesity effects and protection against metabolic diseases				
Male SD rats	Soybean-fermented paste including isoflavone	*In vivo*	Observed	75
Male SD rats	Isoflavones	*In vivo*	Observed	77
Female ovariectomized (OVX) Wistar rats	Isoflavones	*In vivo*	Observed	79
Effects on improving metabolic syndrome				
Female patients with irritable bowel syndrome (IBS)	Isoflavone supplement	*In vivo*	Observed	91
Female wild-type C57BL/6 mice	Genistein	*In vivo*	Observed	92
Male SD rats	Normal chow diet containing isoflavone	*In vivo*	Observed	93
Regulation of blood pressure				
Healthy 30–42-year-old premenopausal women	Novasoy™	*In vivo*	Clinical	98
African American and Caucasian men and women	Isoflavones	*In vivo*	Clinical	100
Improvement of cardiac and lung function				
Healthy male Kunming mice	Genistein	*In vivo*	Observed	106
Rat aortic smooth muscle A10 cells	Genistein	*In vitro*	Observed	107
Post-menopausal women	Isoflavone	*In vivo*	Clinical	110
Adult male SD rats	Daidzein	*In vivo*	Observed	139
Protection against liver damage				
SD rats	Isoflavones	*In vivo*	Observed	114
Male SD rats	Isoflavone	*In vivo*	Observed	116
Male C57BLKS/J-*db*/*db* mice	Genistein	*In vivo*	Observed	119
Improvement of renal dysfunction				

Patients with chronic kidney diseases (CDS)	Isoflavones	*In vivo*	Clinical	120
Ischemia/reperfusion (I/R) mice	Genistein	*In vivo*	Observed	121
I/R-induced mice	Genistein	*In vivo*	Observed	123
Diabetic nephropathic rats	Isoflavone	*In vivo*	Observed	126
Female ICR mice	Genistein	*In vivo*	Observed	127
C57BL/6 mice; human kidney tubular HK2 and human embryo kidney HEK293 cells	Genistein	*In vivo* *In vitro*	Observed	129
Anti-inflammatory effects				
Female BALB/c mice	Isoflavones	*In vivo*	Observed	137
BALB/c mice	Isoflavones	*In vivo*	Observed	138
Adult male SD rats	Daidzein	*In vivo*	Observed	139
Healthy adult male Albino mice	Daidzein	*In vivo*	Observed	141
Human umbilical vein endothelial cell ECV-304	Genistein	*In vitro*	Observed	143
Female ICR mice	Isoflavone	*In vitro*	Observed	146
Anticancer effect				
Healthy women and postmenopausal women	Isoflavone	*In vivo*	Clinical	149
Mouse prostate cancer RM-1 cells; RM-1 tumor-bearing C57BL/6 mice	Genistein	*In vivo* *In vitro*	Observed	150
Patients diagnosed with benign prostate hyperplasia	Isoflavone	*In vivo*	Clinical	152
Breast cancer MCF-7 cells	Genistein and quercetin	*In vitro*	Observed	153
Female SD rats	Genistein and Tamoxifen	*In vivo*	Observed	154
Women with breast cancer	Isoflavone	*In vivo*	Clinical	155
Gastric cancer AGS and MKN45 cells	Genistein	*In vitro*	Observed	156
Human breast cancer MDA-MB-231 cells	Daidzein	*In vitro*	Observed	157
Hepatocellular carcinoma (HCC) cells (Huh7, SK-HEP-1, and SNU-449)	Genistein	*In vitro*	Observed	159
Human lung cancer A549 cells	Genistein	*In vitro*	Observed	160
Small-cell lung cancer H446 cells	Genistein	*In vitro*	Observed	161
Human adeno cervical carcinoma HeLa cells	Pinostrobin	*In vitro*	Observed	162
TC-1 tumor cells derived from lung epithelial cells of C57BL/6 mice	Genistein	*In vivo* *In vitro*	Observed	166
Individuals	Isoflavone	*In vivo*	Clinical	169
Colorectal cancer patients	Isoflavones	*In vivo*	Clinical	170
Chinese women diagnosed with ovarian cancer	Isoflavones	*In vivo*	Clinical	171
Human ovarian cancer BG-1 cells	Genistein	*In vitro*	Observed	172

Patients diagnosed with prostate cancer	Synthetic genistein	*In vivo*	Clinical	173<br>175
Human prostate cancer (PCa) cells	Genistein	*In vitro*	Observed	174
Human prostate cancer cells (PC3, DU145, and LnCaP); male BALB/c nu/nu nude mice	*S*-equol	*In vivo*<br>*In vitro*	Observed	177
Men diagnosed with prostate cancer	Isoflavones	*In vivo*	Clinical	178
Patients diagnosed with prostate cancer	Genistein	*In vivo*	Clinical	179
PCa (LNCap and C4-2B) cells	Daidzein and genistein	*In vivo*	Observed	180
Renal cancer cells (786-O and ACHN)	Genistein	*In vitro*	Observed	182
Rats	Genistein	*In vivo*	Observed	187
Human mammary epithelial tumor MCF-7 cells	Genistein	*In vitro*	Observed	188
Female athymic nude mice	Soy flour containing isoflavones	*In vivo*	Observed	189
Female athymic nude mice	Genistein	*In vivo*	Observed	190
Breast cancer MCF-7 cells	Genistein and daidzein	*In vitro*	Observed	191
Female Wistar rats	Soy formulas	*In vivo*	Observed	193
Female aged 75 years	Super concentrated soy isoflavone	*In vivo*	Clinical	194
Eight-year-old male diagnosed with prepubertal gynecomastia	Dietary soybean and soy-derived consumption	*In vivo*	Clinical	195
Sixty-year-old man diagnosed with gynecomastia	Dietary soy-product	*In vivo*	Clinical	196
Promotion of osteogenesis and the prevention of osteoporosis				
OVX/high-fat diet (HFD) rat	Soy isoflavones plus hop prenylflavanones	*In vitro*	Observed	205
Primary osteoblasts	Soy isoflavone mixture of GS, DZ and GT	*In vitro*	Observed	206
Female SD rats	Isoflavone	*In vivo*	Observed	208
Hsd:ICR (CD-1®) mice	Isoflavones	*In vivo*	Observed	210
Female ICR mice	Isoflavone-enriched whole soy milk powder	*In vivo*	Observed	211
Anti-diabetes effects				
Non-Asian postmenopausal women	Isoflavone	*In vivo*	Clinical	219
Women diagnosed with polycystic ovary syndrome	Isoflavones	*In vivo*	Clinical	220
Female non-obese diabetic mice	Genistein	*In vivo*	Observed	221
General men	Isoflavones	*In vivo*	Clinical	222
Rat skeletal muscle-derived cells; male C57BL/6J-*ob/ob* (*ob/ob*) and C57BL/6J *Jcl* mice	Equol	*In vivo*<br>*In vitro*	Observed	223
Men	Soy protein containing isoflavone	*In vivo*	Clinical	225

Men and women diagnosed with type 2 diabetes (T2D)	Isoflavones	*In vivo*	Clinic	227
Goto-Kakizaki (GK) T2D rats	Soy isoflavones	*In vivo*	Observed	228
Rat pancreatic β-cells (INS-1); human HuH-7 hepatoma and Hepa-1c1c7 mouse hepatoma; five-week-old male ICR mice	*S*-equol	*In vivo* *In vitro*	Observed	230
Skin improvement				
Human keratinocyte (HaCaT) cells; male C57BL/6 mice	Genistein	*In vivo* *In vitro*	Observed	184
Normal human dermal fibroblasts and HaCaT; albino and hairless male mice (SKH:HR-1)	Daidzein and genistein	*In vivo* *In vitro*	Observed	235
Human skin fibroblast cells	Genistein and daidzein	*In vitro*	Observed	236
Female ICR mice	Genistein	*In vivo*	Observed	238
Healthy, non-smoking, and post-menopausal females	Isoflavone	*In vivo*	Clinical	243
Female SD rats	Genistein	*In vivo*	Observed	244
Normal human epidermal keratinocytes; male BALB/c mice	Isoflavone extract	*In vivo* *In vitro*	Observed	245
Porcine ear	Genistein	*In vivo*	Observed	246
Human keratinocytes and fibroblasts	Genistein	*In vitro*	Observed	247
Female Wistar rats	Isoflavone mixed daidzein and genistein	*In vivo*	Observed	248
HaCaT cells; male BALB/c mice	Genistein	*In vivo* *In vitro*	Observed	249
Enhanced quality of life for postmenopausal women based on improved physical and emotional symptoms				
Perimenopausal or postmenopausal women	Isoflavones	*In vivo*	Clinical	165
Perimenopausal and postmenopausal women	Isoflavone	*In vivo*	Clinical	257
Menopausal women	Isoflavone	*In vivo*	Clinical	258
Osteopenic postmenopausal women	Genistein	*In vivo*	Clinical	260
Female C57BL/6 mice	Isoflavone	*In vivo*	Observed	265
Menopausal women	Daidzein	*In vivo*	Clinical	266
Safety of isoflavones in reproductive development				
Infants and child	Soy protein containing isoflavone	*In vivo*	Clinical	273
Weanling male SD rats	Soy protein containing isoflavones	*In vivo*	Observed	275
Improvement of arteriosclerosis				
Pregnant SD rats	Genistein	*In vivo*	Observed	279
Healthy men	Isoflavones	*In vivo*	Clinical	286
Antioxidant activity				
Cells	Genistein, daidzein, And equol	*In vitro*	Observed	287

Chicken macrophage HD11 cells	Equol	*In vitro*	Observed	288
Hops and beer	Flavonoids	*In vivo*<br>*In vitro*	Observed	290
Subterranean and red clover	Phenolic compounds	*In vitro*	Observed	291
Red clover	Isoflavones	*In vitro*	Observed	292
		Antiviral effects		
Chicken embryo fibroblast DF-1 cells	Genistein	*In vitro*	Observed	299
Human A549 pneumocytes and Vero kidney cells	Biochain A	*In vitro*	Observed	301
		Anti-allergic effects		
HaCaT cells; male C57BL/6 mice	Genistein	*In vitro*<br>*In vivo*	Observed	184
Japanese pregnant women	Daidzein and genistein	*In vivo*	Clinical	302

*GS, genistein; DZ, daidzein; GT, glycitein.

Table S2. Neuroprotection and antidepressant effects of isoflavone and its metabolites.

Model	Isoflavone (Conc.)/Assay	Mode of action	Ref
		Neuroprotective effect and improvement of cognitive impairment	
Scopolamine (SCOP)-induced ICR mice aged 4 weeks	Isoflavones (40 mg/kg)/OB	Upregulating phosphorylation levels of extracellular signal-regulated kinase (ERK), cyclic adenosine monophosphate (cAMP) response element-binding protein (CREB) and brain-derived neurotrophic factor (BDNF) expression levels in the hippocampus	55
Virgin female Wistar rats	Genistein [10 and 40 mg/kg body weight (BW)]/OB	Promoting neuroprotection by activating protein kinase B (Akt) pathway during aging	58
Pregnant SD rats	Genistein (~ 80 mg/kg)/OB	Improving neuroprotective effects by inhibiting isoflurane-induced neuronal apoptosis and activating cAMP/CREB-BDNF-tropomyosin receptor kinase B (TrkB)-phosphoinositol 3-kinase (PI3K)/Akt signaling	60
Male ICR mice aged four weeks	6,7,4'-Trihydroxyisoflavone (6,7,4–THIF; 0.5, 1.5 mg/kg)/OB	Increasing neuroprotective effect against SCOP-induced cognitive dysfunction and enhancing learning and memory performance by activating the cholinergic system and the phosphorylated CREB (p-CREB)/BDNF signaling pathway	61
		Antidepressant effects	
Male ICR mice	Genistein (~ 45 mg/kg)/OB	Playing a pivotal role in genistein-mediated anti-depression through the 5-hydroxytryptamine (5-HT; serotonin) system coupled with serotonin 1A (5-HT_{1A}) receptors	66
Depressive pregnant women aged 31–69 years	Isoflavones (GS and DZ; 15.2–36.3 mg/d)/CL	Exhibiting independent inverse relationships between intake of isoflavones and depressive symptoms during pregnancy	67

*OB, observed; CL, clinical, GS, genistein; DZ, daidzein.

Table S3. Anti-obesity, effects on improving metabolic syndrome and regulation of blood pressure effects of isoflavone and its metabolites.

Model	Isoflavone (Conc.)/Assay	Mode of action	Ref
		Anti-obesity effects	
Male SD rats	Soybean-fermented Meju containing isoflavones (100 g/kg)/OB	Reducing body weight (BW) gain, epididymal fat weight, and triglyceride (TG) levels in the serum and liver by inhibiting the lipogenic enzymes fatty acid synthase (FAS), malic enzyme (ME), and lipoprotein lipase (LPL) in epididymal adipose tissues and inactivating glucose-6-phosphate dehydrogenase (G6PD) in the liver	75
Male SD rats	Isoflavone mixture containing 50.98% DZ: 30.36% GT: 8.80% GC: 1.24% DA: 0.06% GE (50 mg, 150 mg, and 400 mg/kg)/OB	Improving lipid homeostasis by inhibiting mammalian target of rapamycin complex 1 (mTORC1) activity through the phosphorylation of AKT and ribosomal protein S6	77
Female OVX Wistar rats	Isoflavones (431 mg/kg/d)/OB	Lowering BW, visceral fat mass, serum leptin levels, and skeletal muscle weight and reducing adipocyte area by downregulating hepatic sterol regulatory element-binding protein 1c (SREBP-1c), acetyl-CoA carboxylase (ACC), FAS, and peroxisome proliferator-activated receptor-gamma (PPARγ) at the transcriptional level	79
		Effects on improving metabolic syndrome	
Female patients with IBS aged 18–75 years	Isoflavone supplement containing 10 mg DZ, 8.5 mg GS, and 0.5 mg GC (40 mg/d)/OB	Improving inflammation and intestinal permeability of women with IBS by reducing plasma inflammatory biomarkers and fecal protease activity, but not antioxidant activity	91
Female C57BL/mice aged 8–12 weeks	Genistein (5, 20, 40 and 100 mg/kg)/OB	Preventing inflammatory bowel disease by alleviating symptoms of dextran sodium sulfate-induced colitis; increasing BW, and reducing inflammation score; shifting M1 to M2 phenotype; and reducing systemic cytokine levels	92
Male SD rats weighted 180 ± 10 g	Normal chow diet containing isoflavone (0.038%)/OB	Increasing fecal pellet number, fecal water content, intestinal transit rate, serum concentrations of substance P, and vasoactive intestinal peptide; inducing changes in intestinal microbiota; upregulating genes associated with glycan biosynthesis and metabolism	93
		Regulation of blood pressure	
Healthy premenopausal	Novasoy™ (246 mg)	Normalizing systolic blood pressure (SBP) by modulating the physiological effects of calcium; decreasing	98

women aged 30–42 years	containing 68.3 mg of aglycone equivalent of DZ (30 mg), GS (30 mg), and GT (8.3 mg); 1 tablet for 5 d per week/CL	diastolic blood pressure independent of calcium concentration	
African American and Caucasian men and women aged 18–30 years	Isoflavones (0.74-2.50 and 2.51-222.7 mg/d)/CL	Lowering systolic blood pressure in a dose-dependent manner	100

*OB, observed; CL, clinical, DZ, daidzein, DA, daidzin; GS, genistein; GE, genistin; GC, glycitin; GT, glycitein.

Table S4. Improvement of cardiac function, Protection against liver damage, and Improvement of renal dysfunction of isoflavone and its metabolites.

Model	Isoflavone (Conc.)/Assay	Mode of action	Ref
	Improvement of cardiac function		
Healthy male Kunming mice weighing 20–25 g	Genistein (100 mg/kg/d)/OB	Suppressing tumor growth factor beta (TGFβ)-activated kinase 1 (TAK1) expression and producing anti-fibrotic effects by blocking the TAK1/mitogen-activated protein (MAP) kinase kinase 4 (MKK4)/c-Jun N-terminal kinase (JNK) signaling pathway	106
Rat aortic smooth muscle A10 cells	Genistein (5 mg/kg BW)/OB	Decreasing leptin-stimulated reactive oxygen species (ROS) generation and phosphorylation of the p44/mitogen-activated protein kinase (MAPK) signal transduction pathway	107
Post-menopausal women aged mean 55 years	Isoflavone (66 mg/d)/CL	Improving cardiovascular disease risk markers by reducing metabolic parameters and SBP and reducing the risk of myocardial infarction, cardiovascular disease, and death due to cardiovascular disease	110
	Protection against liver damage		
SD rats	Isoflavones (10 or 20 mg/kg)/OB	Alleviating hepatic steatosis and delaying the progression of non-alcoholic fatty liver disease (NAFLD) by inhibiting lipogenesis and promoting fatty acid oxidation in the liver	114
Male SD rats weighting approximately 205 g	Isoflavone (10 and 20 mg/kg/d)/OB	Reducing high fat diet (HFD)-induced lipid accumulation and serum alanine transaminase (ALT) and improving liver lobule structure; downregulating sterol SREBP-1c and FAS and upregulating PPARα	116
Male C57BLKS/J-*db*/*db* mice	Genistein (0.5–0.6 g/kg/d)/OB	Causing oxidative stress, endoplasmic reticulum stress, and adenosine monophosphate (AMP)-activated protein kinase (AMPK) inactivation; downregulating the expression of genes associated with hepatic inflammation, lipid peroxidation, and fibrosis	119
	Improvement of renal dysfunction		

Patients with CKD	Isoflavones (35.5–61 mg/d)/CL	Reducing blood urea nitrogen, serum creatinine, serum phosphorus, C reactive protein (CRP), and proteinuria in patients in the predialysis stage	120
I/R mice	Genistein (~ 15 mg/kg)/OB	Reducing renal I/R-induced cell death by reducing p53 and p21 expression; stimulating renal cell proliferation by increased proliferating cell nuclear antigen (PCNA) expression; upregulating Sirtuin 1 (SIRT1) expression	121
I/R-induced mice aged 7–9 weeks	Genistein (~ 15 mg/kg)/OB	Reducing I/R-induced cell death; stimulating renal cell proliferation; upregulating SIRT1, p53, and p21 expression and downregulating PCNA expression	123
Diabetic nephropathic (DN) rats	Isoflavone/OB	Reducing urinary protein quantity; alleviating renal interstitial pathological damage; downregulating the expression of Wnt4, β-catenin, and TGF-β1 in the renal interstitium	126
Female ICR mice aged 5.5 weeks	Genistein (0.025 and 0.1%)/OB	Improving levels of fasting blood glucose (FBG); attenuating kidney oxidative stress by decreasing levels of malondialdehyde (MDA), blood urea nitrogen (BUN), and plasma creatinine; improving inflammatory and oxidative stress markers	127
C57BL/6 mice; human kidney tubular HK2 and human embryo kidney HEK293 cells	Genistein (10 mg/kg in mice; 15 μM in cells)/OB	Exhibiting anti-renal fibrosis activities by recovering the epigenetic loss of Klotho through the inhibition of histone 3 deacetylation of Klotho promoter and normalization of promoter DNA hypermethylation by suppressing DNA methyltransferase DNA methyltransferase 1 and 3a (DNMT1/3a)	129

*OB, observed; CL, clinical.

Table S5. Anti-inflammatory effects of isoflavone and its metabolites.

Model	Isoflavone (Conc.)/Assay	Mode of action	Ref
Female BALB/c mice	Isoflavones (5 mg/d; 250 mg/kg)/OB	Reducing infiltration and activation of alveolar macrophages and neutrophils in bronchoalveolar and lung parenchyma compartments upon irradiation; protecting F4/CD11c interstitial macrophages as an immunoregulator; producing decreased nitric oxide synthase 2 (NOS2) and increased arginase-1 (Arg1)	137
BALB/c mice aged 5–6 weeks	Isoflavones (1 mg/d)/OB	Preserving Arg-1 activation and nuclear factor kappaB (NF-κB) inactivation in granulocytic myeloid-derived suppressor cells under irradiation; controlling pro-inflammatory cytokine production	138
Adult male SD rats weighing 250–300 g	Daidzein (2, 4 and 8 mg/kg)/OB	Inhibiting toll-like receptor 4 (TLR4)-myeloid differentiation primary response 88 (MyD88)-NF-κB pathway	139
Healthy adult male Albino mice weighting 25–35 g	Daidzein (0.2 and 1.0 mg/kg BW)/OB	Decreasing bacterial population in peritoneal fluid, blood, and lung tissue; reducing tumor necrosis factor alpha (TNFα) and nitrite levels in the plasma; inhibiting lung injury by inactivating vascular permeability and myeloperoxidase (MPO) activity; increasing the expression of inducible NOS (iNOS) and glucocorticoid receptor α/β (GCRα/β) in septic lungs	141

Human umbilical vein endothelial ECV-304 cell	Genistein (~ 100 μM)/OB	Maintaining cell population and preventing changes in cell morphology and ROS accumulation by downregulating NF-κB, transcription factor p65, interleukin 6 (IL-6), and intracellular adhesion molecule 1 (ICAM-1); alleviating apoptosis and proliferation dysfunction of endothelial cells	143
Female ICR mice weighing 22.66 ± 0.12 g and aged five weeks	Soy product (daily diet containing 0.5% isoflavone)/OB	Alleviating dextran sulfate sodium (DSS)-induced growth inhibition and colonic inflammatory response, oxidative stress, and colonic barrier abnormality by downregulating TLR4/Myd88 signaling	146

*OB, observed.

Table S6. Anticancer effects of isoflavone and its metabolites.

Model	Isoflavone (Conc.)/Assay	Mode of action	Ref
Women and postmenopausal women aged 25–74 years	Isoflavone (0–122, 123–496 and ≥497 μg/d)/CL	Reducing breast cancer risk through isoflavone intake in women after menopause, particularly the tumor group with estrogen (ER) and progesterone receptor (PR)	149
Mouse prostate cancer cells RM-1 cells; RM-1 tumor-bearing C57BL/6 mice	Genistein (~ 120 μM)/OB	Increasing ROS-induced oxidative damage by downregulating the intracellular expression of apurinic/apyrimidinic endonuclease 1 (APE1) and reducing oxidative DNA repair in prostate cancer cells	150
Participants diagnosed with benign prostate hyperplasia	Isoflavone (40 mg/d)/CL	Being safe and tolerant with isoflavone-mediated anticancer activity	152
Breast cancer MCF-7 cells	Genistein and quercetin (~ 100 μM/L)/OB	Inhibiting cellular proliferation through the intrinsic and extrinsic apoptotic pathways in a dose- and time-dependent manner; upregulating Fas ligand (FasL), Fas-associated protein with death domain (FADD), cytochrome C, truncated Bid, caspase-9, and caspase-3 and downregulating PI3K and AKT protein; inducing increased estrogen receptor beta (ERβ) expression and decreased ERα	153
Female SD rats with mammary tumors induced by 7,12-dimethylbenz(a)anthracene	Genistein (500 ppm/d) and Tamoxifen (337 ppm/d)/OB	Downregulating the unfolded protein response (UPR), autophagy-related genes [glucose-regulated protein 78 (GRP78), inositol-requiring enzyme 1 alpha (IRE1α), activating transcription factor 4 (ATF4), and beclin-1 (BECN1)], and genes associated with immunosuppression [TGFβ and forkhead box P3 (Foxp3)]; upregulating cluster of differentiation 8a (CD8a) as a cytotoxic T-cell marker	154
Women diagnosed with breast cancer	Isoflavone (GS, DZ and GT; ≥1.5 mg and <0.3 mg/d)/CL	Reducing all-cause mortality through higher dietary intake in patients with breast cancer	155

Gastric cancer AGS and MKN45 cells	Genistein (10 µg/mL)/OB	Reducing the expression of zinc finger protein Gli1 and CD44 acting multifunctional cell surface adhesion receptor at transcriptional and translational levels; inhibiting cell migration ability	156
Human breast cancer MDA-MB-231 (metastatic, estrogen-unresponsive) and MCF-7 (estrogen-responsive) cells	Daidzein (50 and 100 µM)/OB	Exhibiting reduced cell viability and decreased cell migration by inducing a conformational change in DNA, lipids, and protein components associated with antioxidant systems	157
Hepatocellular carcinoma SNU-449 cells	Genistein (0, 200, 400, 600 µM)/OB	Inhibiting the proliferation of SNU-449 cells in a concentration-dependent manner; enhancing genistein-induced apoptosis by downregulating thioredoxin-1 (Trx1), increasing intracellular production of ROS, and activating apoptosis signal-regulating kinase 1 (ASK1), JNK, and p38 as signal transduction mediators	159
Human lung cancer A549 cells	Genistein (0, 10, 20, 50, 100 and 200 µM)/OB	Inhibiting cell growth by inducing cell apoptosis following caspase-3/9 activation in a dose-dependent manner; activating microRNA-27a (miR-27a)-mediated MET signaling	160
Small-cell lung cancer H446 cells	Genistein/OB	Reducing the proliferation and migration ability of H446 cell through apoptosis and G2/M cell cycle arrest; downregulating forkhead box protein M1 (FoxM1) and its target genes (i.e., Cdc25B, cyclin B1, and survivin) associated with cell cycle and apoptosis	161
Human adeno cervical carcinoma HeLa cells	Pinostrobin (10, 30, 50, 100 and 200 µM)/OB	Inducing apoptosis through caspase dependent pathways by increasing ROS accumulation and ROS-mediated mitochondrial damage; upregulating the expression of proteins associated with extrinsic and intrinsic signaling pathways	162
Tumor TC-1 cells; C57BL/6 mice aged 6–8-weeks	Genistein (20 mg/kg)/OB	Increasing lymphocyte proliferation, interferon gamma (IFN-γ) expression, and lactase dehydrogenase (LDH) release	166
Individuals aged 35 years or older	Isoflavone (23.8~77.4 mg/d)/CL	Having a protective effect against stomach cancer of soy isoflavone	169
Patients diagnosed with colorectal cancer	Isoflavones (≥20.89 mg/day)/CL	Reducing the overall risk for colorectal cancer, including distal colon and rectal cancer	170
Chinese women diagnosed with ovarian cancer	Isoflavones (30.3 mg/d; mixture of DZ (12.4 mg), GS (15.5 mg) and GT (2.4 mg)/CL	Reducing ovarian cancer risk in a dose-dependent manner	171
Human ovarian cancer BG-1 cells	Genistein (100 µM)/OB	Inhibiting epithelial-mesenchymal transition and migration abilities of ovarian cells enhanced by estrogenic chemicals (17β-estradiol, bisphenol A, and	172

		nonylphenol); downregulating estrogenic chemicals-mediated TGF-β signaling	
Patients diagnosed with prostate cancer	Synthetic genistein (30 mg/d)/CL	Regulating the expression of androgen-related biomarker [kallikrein-related peptidase 4 (KLK4)] and cell cycle-related biomarker ($p27^{Kip1}$) in tumor cells; regulating the expression of genes involved in developmental processes, stem cell markers, proliferation, and transcriptional regulation; inducing decreased MYC (acting proto-oncogene and bHLH transcription factor) activity and increased phosphatase and tensin homolog (PTEN) activity	173 175
Human prostate cancer (PCa) PC3 and DU145 cells	Genistein (25 μM/L)/OB	Regulating homebox (HOX) transcript antisense RNA (HOTAIR) in PCa cells; decreasing proliferation, migration and invasion and inducing apoptosis and cell cycle arrest in HOTAIR-knockdown PCa cells; upregulating tumor suppressor miR-34a in both PC3 and DU145 PCa cells	174
Human prostate cancer PC3, DU145 and LnCaP cells; male BALB/c nu/nu nude mice aged four weeks weighing 18–20g)	S-equol (0, 50, 100, 150 and 200 μM)/OB	Inhibiting the growth of all three cell lines (PC3, DU145 and LnCaP); promoting cell cycle arrest in the G2/M phase in PC3 cells by downregulating cyclin B1 and cyclin-dependent kinase 1 (CDK1) and upregulating CDK inhibitors (p21 and p27), as well as activating apoptosis by upregulating FasL and the expression of proapoptotic Bim; activating the Akt/forkhead box O3 (FOXO3a) pathway associated with prostate cancer cell survival, cell cycle progression, and apoptosis	177
Men diagnose with prostate cancer	Isoflavones (34 mg/50 g slice of soy bread)/CL	Reducing type 1 T helper (TH1) and myeloid-derived suppressor cell (MDSC)-associated cytokines but not TH2 and TH17 cytokines	178
Men diagnosed with prostate cancer aged ≥40 years	Genistein (513.0 and 728.6 ng/mL)/CL	Contributing to the low risk of prostate cancer at a high concentration of plasma genistein in people with Chinese ethnicity	179
Bone metastatic LNCaP-derivative PCa C4-2B cells	Daidzein (25–200 μM), genistein (25–200 μM) or their combinations (25 or 50 μM of each)/OB	Increasing the synergistic effect by inhibiting cell proliferation and activating apoptosis; increasing the apoptotic effects in concentration of 25 μM daidzein/50 μM genistein and 50 μM daidzein/50 μM genistein in C4-2B cells	180
Renal cancer 786-O and ACHN cells	Genistein (0, 30, 60 and 90 μM)/OB	Inhibiting the activity of renal cancer stem cells (CSCs) by repressing tumor sphere formation, decreasing renal CSCs markers, inhibiting proliferation, and inducing apoptosis; downregulating the oncogenic sonic hedgehog (Shh) pathway	182
Human interventional or observational data (MEDLIN, Embase, the	Safety and efficacy of soy- and red clover-derived isoflavones in for	Evidence showing harm from intake of soy isoflavone regarding risk of breast cancer or recurrence based on long term observational data; protective effects against breast cancer and recurrence through the consumption a traditional Japanese diet (2–3 servings daily,	183

Cochrane Library, and AMED)	breast cancer/OB	comprising 25–50 mg isoflavones); providing a synergetic effect in women taking tamoxifen plus soy isoflavone without side effects; no breast cancer-promoting effects for red clover	
Rats	Identification of differentially regulated proteins from the mammary glands of rats exposed to bisphenol A (BPA) and genistein with estrogenic properties/OB	Upregulating annexin A2, vascular endothelial growth factor receptor 2 (VEGFR2), and phospho-Akt in BPA-exposed rats, but downregulating these proteins in genistein-treated rats; activating GRP-78 and heat shock protein 70 (HSP-70) in response to BPA, and leading to upregulated fetuin B and downregulated phosphoglycerate kinase (PGK) in response to genistein	187
Human mammary epithelial tumor MCF-7 cells and primary breast adipose fibroblasts (BAFs)	Genistein (~ 100 μM)/OB	Improving the efficacy of breast cancer treatment with aromatase inhibitors by disabling the growth inhibitory action of the aromatase inhibitor fadrozole in primary BAFs and estrogen-dependent MCF-7 tumor cells	188
Female athymic nude mice	Soy flour (180 g) and mixture of isoflavones and genistein (750 μM/g; 750 ppm)/OB	Maintaining tumor growth and size; upregulating pS2 (Alzheimer's disease gene for apoptosis), progesterone receptor, and cyclin D1 (a regulator of cell cycle progression) and downregulating B-cell lymphoma 2 (Bcl2) as an apoptosis regulator at the transcriptional level	189
Female athymic nude mice	Genistein (750 ppm) and genistin (1200 ppm)/OB	Stimulating estrogen-dependent breast cancer cell growth in vivo in the presence of the glycoside genistin and aglycone genistein	190
Breast cancer MCF-7 cells	Genistein and daidzein (1, 5 and 10 μM)/OB	Stimulating the proliferation of estrogen receptor ERα(+) breast cancer cells at low concentrations, but not ERβ(+) cells *in vitro*; increasing the risk of breast cancer development and progression in women following consumption of high-dose isoflavone supplements when it binds to ERα(+)	191
Evaluating literatures associated with the risk developing endometriosis and/or the occurrence of dysmenorrhea	Food product associated with isoflavone/OB	Lowering the risk of developing endometriosis by taking fruits and vegetables, fish oils, dairy products rich in calcium and vitamin D, and omega-3 fatty acids	192
Female Wistar rats aged 4 weeks	Soy formulas (~ 60%)/OB	Exhibiting faster growth; increasing the intensity of pelvic pain as well as the volume of ectopic foci in rats fed >10% dietary soy; increasing tissue levels of MDA and glutathione (GSH; reduced form)	193

Women diagnosed with malignant mullerian carcinosarcoma aged 75 years	Super concentrated soy isoflavone (DZ/DA;GT/GC; GE/GS; 72 mg/d)/CL	Promoting ureteral mullerian carcinosarcoma arising in endometriosis foci after extensive phytoestrogen supplementation	194
Male diagnosed with prepubertal gynecomastia aged 8 years	Dietary soybean and soy-derived intake/CL	Contributing to the abnormal development of breast tissue in adolescent and adult males from the excessive intake of dietary soy	195
Man diagnosed with gynecomastia aged 6 years	Dietary soy-product (soy milk) consumption/CL	Resolving breast tenderness and recovering normal estradiol concentration by discontinuing soy milk consumption after a daily intake of soy milk	196

*OB, observed; CL, clinical, DZ, daidzein, DA, daidzin; GE, genistin; GS, genistein; GC, glycitin; GT, glycitein.

Table S7. Anti-osteoporosis and anti-diabetes effects of isoflavone and its metabolites.

Model	Isoflavone (Conc.)/Assay	Mode of action	Ref
	Promotion of osteogenesis and the prevention of osteoporosis		
OVX/HFD rat	Soy isoflavones (300–350 mg/g) plus hop prenylflavanones (Soy-Hop; ~ 300 mg/kg)/OB	Suppressing food intake, BW gain, fat mass, and circulating levels of leptin, adiponectin, low-density lipoprotein (LDL)-cholesterol, total cholesterol, TG, glucose, and insulin by consuming high-dose Soy-Hop; attenuating the elevation of osteocalcin (OC), alkaline phosphatase (ALP), and collagen type 1 cross-linked C telopeptide (CTX) in the plasma; regulating the receptor activator for nuclear factor κ B ligand (RANKL)/osteoprotegerin (OPG) gene expression ratio in femur	205
Primary osteoblasts	Soy isoflavone (0.1, 0.2 and 0.3 M) comprising of GS, DZ and GT, accounting for 60, 20 and 13%, respectively/OB	Increasing RANKL and ALP, and OPG levels in osteoblast cells; promoting cell proliferation and differentiation by activating Wnt/β-catenin pathway	206
Female SD rats aged 3 weeks weighing 35–40 g	Isoflavone (10 and 50 mg/kg BW)/OB	Exhibiting increased ALP activity and decreased OC; enhancing bone volume, trabecular number, bone mineral density (BMD), and trabecular thickness; stimulating longitudinal bone growth in low-dose supplementation; improving bone quality (BMD and structural parameters) with high-dose supplementation	207
Female rats aged 6 months	Isoflavone (80, 200 and 350 mg/kg/d)/OB	Improving the bone quality by increasing histomorphometric parameters, the content of glycosaminoglycans and mature type I collagen fibers in a dose-dependent manner, exception for cortical and trabecular bone, in femurs of rats	208
CD-1 mice	Isoflavones	Improving BMD and trabecular inter-connectivity in long bones and lumbar spine	210

(multifunctional research animal model)	(2 mg DZ/kg BW; 5 mg GS/kg BW; or 2 mg DZ + 5 mg GS/kg BW)/OB		
Female ICR mice	Isoflavone-enriched whole soy milk powder (2%, 10%, and 20%)/OB	Increasing the concentration of serum calcium, OC, procollagen 1 N-terminal propeptide (P1NP), and OPG; inhibiting ALP and tartrate-resistant acid phosphatase (TRAP) 5b activity; preventing BMD reduction in a dose-dependent manner; promoting bone formation	211
		Anti-diabetes effects	
Non-Asian post-menopausal women from 1970 to October 2010	Isoflavone (≈ 100 mg/d)/CL	Reducing BW, blood glucose level, and fasting insulin levels in women after menopause	219
Women with polycystic ovary syndrome aged 18-40 years	Isoflavones (50 mg/d)/CL	Decreasing circulating serum levels through insulin resistance, free androgen index, serum TG, and MDA levels; increasing the quantitative insulin sensitivity check index and total GSH in the plasma	220
Female non-obese diabetic (NOD) mice	Genistein (2, 6 and 20 mg/kg)/OB	Decrease in the incidence of diabetes (blood glucose ≥ 250 mg/dL) and severe diabetes (blood glucose ≥ 400 mg/dL) ranging from 55 to 79% in the presence of genistein; decrease in higher total incidence of diabetes in animals treated with genistein	221
Men aged 45–75 years	Isoflavones (66 mg/d)/CL	Reducing type I collagen crosslinked beta C-telopeptide (βCTX), glycosylated hemoglobin (HbA1c), and homeostatic model assessment of insulin resistance (HOMA-IR)	222
Rat skeletal muscle-derived cell line of L6 myoblasts; male C57BL/6J-*ob*/*ob* (*ob*/*ob*) and C57BL/6J Jcl mice aged 5 weeks	Equol (~ 1 μM)/OB	Increasing glucose transporter 4 (GLUT4) translocation to the plasma membrane through AMPK activation; suppressing FBG levels and downregulating the genes encoding hepatic enzymes associated with glucose metabolism	223
Men aged 45–75 years with an early morning total testosterone concentrations of less than 12 nM/L	Soy protein (15 g/d) containing isoflavone (66 mg/d)/CL	Reducing type I collagen crosslinked βCTX, HbA1c, and HOMA-IR	225
Men and women aged 25-42 and 40-75 years diagnosed with type 2 diabetes (T2D) in US	Isoflavones (0.62–13.13 mg/week)/CL	Lowering T2D risk	227

Male Goto-Kakizaki (GK) type 2 diabetic rats aged 20 weeks	Soy isoflavones from hypocotyl (DZ:GT:GS= 61.7%:21.5%:15.2%; 150 mg/kg BW)/OB	Reducing FBG, CRP, and lipid peroxide; decreasing glycosylated serum protein, TNFα, and IL-6; inhibiting intestinal α-glucosidase; decreasing glucose transport potency into brush border membrane vesicles or Caco-2 cells	228
Rat pancreatic β-cells (INS-1), human hepatoma (HuH-7), and mouse hepatoma (Hepa-1c1c7); male ICR mice aged 5 weeks	*S*-equol (10 μM in cells and 20 mg/kg/d in mice)/OB	Increasing population and mass of β-cell; highlighting growth, insulin secretion, and cAMP-response element (CRE)-mediated transcription in INS-1 cells; promoting glucose-stimulated insulin secretion in mouse pancreatic islets	230

*OB, observed; CL, clinical, DZ, daidzein, DA, daidzin; GE, genistin; GS, genistein; GT, glycitein.

Table S8. Skin improvement of isoflavone and its metabolites.

Model	Isoflavone (Conc.)/Assay	Mode of action	Ref
Human keratinocyte (HaCaT) cells; male C57BL/mice aged 6–8 weeks	Genistein (10, 20 and 40 mg/kg)/OB	Antipruritic and anti-inflammatory effects by inhibiting the production of pro-inflammatory cytokines and intracellular mitogen-activated protein kinase kinase 2 (MAP2K2)/extracellular-signal-regulated kinase (ERK) signaling in HaCaT cells and mice	184
Normal human dermal fibroblasts and keratinocytes (HaCaT cells); albino and hairless male mice (SKH:HR-1) aged 7 weeks	Daidzein and genistein (each 10 μM)/OB	Inhibiting IL-6 production and MAPK signaling in keratinocytes and fibroblasts in the presence of genistein; reducing macrophage infiltration to the dermis and decreasing the expression of iNOS and cyclooxygenase 2 (COX-2) in mice	235
Human skin fibroblasts (BJ-5ta cells)	Genistein and daidzein (2–60 μM)/OB	Preventing ultraviolet B (UVB; 60 mJ/cm^2)-induced DNA damage with both the isoflavone glucosides at a specific concentration and combination with an aglycone	236
Female ICR mice aged 4 weeks	Genistein (0.25 and 1 g/kg)/OB	Accelerating the rate of wound closure; activating the expression of copper/zinc superoxide dismutase (Cu/Zn-SOD), manganese superoxide dismutase (Mn-SOD), catalase, and GSH peroxidase in wound skin tissue; modulating NF-κB and TNF-α expression; lowering hepatic lipid peroxidation	238
Healthy, non-smoking, and post-menopausal females	Isoflavone (40 and 70 mg/d)/CL	Reducing the depth of facial wrinkles by increasing the deposition of new collagen fibres in the dermis	243
Female SD rats aged six months	Genistein (1 mg/kg/d) /OB	Increasing skin flap viability by enhancing SOD activity and Bcl-2 expression, and upregulating genes associated with angiogenesis/inflammation promotion	244

		[connective tissue growth factor (CTGF), C-X-C motif chemokine 5 (CXCL5), IL-6, integrin beta 3 (ITGB3), matrix metalloprotenase-14 (MMP-14), and vascular endothelial growth factor A (VEGF-A)] and angiogenesis inhibition [endostatin (COL18A1), tissue inhibitor of metalloproteinase 2 and 3 (TIMP-2/3)]	
Human epidermal keratinocytes (NHEKs); male BALB/c mice aged 8–11 weeks; normal	Isoflavone extract (10 mg/mL in mice; 1, 3 and 10 μg/mL in cell)/OB	Reducing trans-epidermal water loss (TEWL), erythema, blood flow speed, and ear thickness; increasing surface skin hydration; attenuating epidermal hyperplasia and inflammatory cell infiltration *in vivo* in mice; downregulating IL-22, IL-17A, and TNF-α-induced MAPK, NF-κB, and Janus kinase (JAK)-signal transducer and activator of transcription (STAT) activation in normal human epidermal keratinocytes *in vitro*	245
Porcine ear	Genistein (0.03%)/OB	Protecting the skin against oxidative damage caused by UVA/ UVB light through their high antioxidant activity	246
Human keratinocytes and fibroblasts	Genistein (~ 100 μM)/OB	Improving cell viability, mitochondrial membrane potential, GSH levels, and proliferation rate by suppressing nitric oxide (NO) and ROS release under peroxidation conditions; activating PI3K-Akt and p38-MAPK signaling pathways, as well as NOS	247
Female Wistar rats aged 3 months	Isoflavone mixed DZ and GS (2 and 20 mg/kg BW/d)/OB	Improving the thickness of skin epidermis and collagen fibers in the dermis, as well as the amount of fibers; decreasing catalase activity in skin homogenates; inhibiting lipid peroxidation in a dose-dependent manner	248
HaCaT cells; male BALB/c mice aged 7-8 weeks;	Genistein (50 and 100 μM in mice; 3.12-200 μM in cells)/OB	Inhibiting the proliferation of human keratinocyte HaCaT cells and inhibiting the expression of inflammatory factors in a dose-dependent manner; downregulating TNFα, IL-6, IL-23, and VEGFA expression; inhibiting phosphorylated STAT3 (pSTAT3) expression in the dorsal skin of imiquimod (IMQ) mice and in TNF-α-induced HaCaT cells; inhibiting the nuclear translocation of NF-κB and the phosphorylation of I-kBα (pI-kBα)	249

*OB, observed; CL, clinical; DZ, daidzein; GS, genistein.

Table S9. Enhanced quality of life for postmenopausal women and safety in reproductive development of isoflavone and its metabolites.

**Model**	**Isoflavone (Conc.)/Assay**	**Mode of action**	**Ref**
Enhanced quality of life for postmenopausal women based on improved physical and emotional symptoms			
Perimenopausal or postmenopausal women aged 45 or more	Isoflavones (50 mg/d)/CL	Improving both somatic and urogenital symptoms of menopause as well as the quality of women around and after menopause	165

Women around and after menopause	Isoflavone (100 mg/d)/CL	Improving the menopause rating scale [i.e., hot flashes (HFs) and urogenital subscale] score	257
Menopausal women aged 45–65 years	Isoflavone (60 mg/d)/CL	Improving the Kupperman index (particularly hot HFs); improvement of sleep quality and psychophysical wellness parameters	258
Osteopenic postmenopausal women aged 49–67 years	Genistein (54 mg/d)/CL	Improving quality of life (such as physical and mental health, depression, and social function) in women with osteopenia after menopause	260
Female C57BL/6 mice aged 12 months weighing 200–240 g	Isoflavone (5, 10 and 20 mg/kg/d)/OB	Increasing vaginal blood flow in a dose-dependent manner; improving female sexual dysfunction (FSD) by modulating the levels of hormones in the serum and histologic changes of the vagina through the endothelial nitric oxide synthase (eNOS) pathway	265
Menopausal women aged mean 50.5–58.5 years	Daidzein (63 mg/d)/CL	Lowering the incidence and severity of HFs in menopausal women	266
Safety of isoflavones in reproductive development			
Infants and children	Soy protein (infants from birth until the age of 3 years; children aged 7.8–10.5 years; 6 g of soy product/3 d)/CL	Exhibiting no association between infantile soy-based formula consumption and growth and puberty parameters, including body mass index (BMI) and *z* scores	273
Weanling male SD rats	Soy product containing total isoflavones (3.21 g/kg) including GS (1.87 g/kg), GS aglycone (1.08 g/kg), DZ (1.22 g /kg), and DZ aglycone (0.69 g/kg)/OB	Regulating genes associated with macromolecule modifications including ubiquitination and histone methylation	275

*OB, observed; CL, clinical; DZ, daidzein; GS, genistein.

Table S10. Improvement of arteriosclerosis, antioxidant activity, and antiviral and anti-allergic effects of isoflavone and its metabolites.

Model	Isoflavone (Conc.)/Assay	Mode of action	Ref
Improvement of arteriosclerosis			
Pregnant SD rats	Genistein	Attenuating the deleterious effects of BSA-derived xenoestrogen on epithelial cell proliferation, androgen	279

	(~ 5.5 mg/kg/d)/OB	receptor expression and prostatic architecture in male offspring	
Male healthy men with total cholesterol (TC) >5.17 mmol/L and <6.21 mmol/L intervention	Isoflavones (50 mg /100 g BW)/CL	Improving total cholesterol, non-high-density lipoprotein C (NHDL-C) [LDL + intermediate-density lipoprotein (IDL) + very-low-density lipoprotein (VLDL)] cholesterol fractions) and electronegative LDL concentrations; maintaining HDL-C level	286
		Antioxidant activity	
In vitro assay	Genistein, daidzein and *S*-equol/OB	Characterizing antioxidant properties of genistein, daidzein, and the daidzein metabolite–*S*-equol by evaluating detoxification of superoxide anion, hydroxyl radical, 2,2–diphenyl–1-picrylhydrazyl radical (DPPH), and hydrogen peroxide (H_2O_2) using chemiluminescence and spectrophotometry techniques	287
Chicken macrophage HD11 cells	Equol (~ 160 μM/L)/OB	Producing lower levels of MDA and higher levels of GSH; elevating enzymatic activity of total SOD and iNOS; upregulating the expression of SOD2 and GSH transferase (GST), TLR4, TNFα, and IL-1β at the transcriptional level	288
Antioxidant activity of hop and beer containing flavonoids	Hops and beer/OB	Exhibiting a broad spectrum of biological activities, including estrogenic, anticancer, neuroprotective, anti-inflammatory, and antimicrobial properties in hops and beer containing 8-prenylnaringenin (8-PN), 6-prenylanaringenin (6-PN), 6,8-diprenylnaringenin (6,8-DPN) and 8-geranylnaringenin (8-GN)	290
Extraction and purification of phenolic compounds	Subterranean clover (*Trifolium subterraneum*) and red clover (*Trifolium pratense*)/OB	Producing biologically active flavonoid compounds, including isoflavone; identifying clovamide, four flavonols, and 15 isoflavones presenting as glucosides or glucosyl malonates; comprising derivatives of isoflavones, such as genistein, biochanin A, and formononetin in subterranean clover extracts; exhibiting higher isoflavone concentration in subterranean clover compared with soybean or red clover	291
Determining the maximized content of isoflavones (particularly ERβ) from red clover sprouts cultivated under different conditions (light, time, and temperature)	Red clover /OB	Producing the highest isoflavone (especially formononetin) content from sprouts cultivated for 10 d under continuous white light at 25 °C (562 mg/100 g fresh mass)	292
		Antiviral effects	
Chicken embryo fibroblast DF-1 cells	Genistein (0, 12.5, 25, 50 and 100 μM)/OB	Inhibiting viral gene expression and decreasing viral protein expression in cell supernatant and the cytoplasm without changing virus receptor expression and viral attachment; inhibiting virus transcriptions and release in host cells	299

Human lung carcinoma A549 cells and African green monkey kidney Vero cells	Biochain A (0, 10, 20 and 40 μM)/OB	Inhibiting influenza A nucleoprotein production and replication in A549 cells infected with the highly pathogenic avian influenza H5N1 virus; reducing H5N1 infectious titres and virus-induced caspase 3 cleavage, and the nuclear export of viral ribonucleoprotein (RNP) complexes; affecting cellular signaling pathways by inactivating AKT, ERK 1/2, and NF-kB; inhibiting the virus-induced production of IL-6, IL-8, interferon gamma-induced protein 10 (IP-10), and TNF-α	301
		Anti-allergic effects	
Human keratinocyte (HaCaT) cells; male C57BL/6 mice aged 6–8 weeks	Genistein (10, 20 and 40 mg/kg)/OB	Inhibiting MAP2K2- and MAPK-related signaling pathway by downregulating the expression of inflammatory factor and chemokines by the phosphorylation of ERK and STAT3	184
Pregnant women	Daidzein and genistein (9.0 and 15. mg/d of each)/CL	Inducing anti-allergic rhinitis	302

*OB, observed; CL, clinical.

Abbreviation:

ACC, acetyl-CoA carboxylase; Akt, protein kinase B; ALP, alkaline phosphatase; ALT, alanine transaminase; AMP, adenosine monophosphate; AMPK, AMP-activated protein kinase; APE1, apurinic/apyrimidinic endonuclease 1; Arg1, arginase-1; ASK1, apoptosis signal-regulating kinase 1; ATF4, activating transcription factor 4; BAFs, breast adipose fibroblasts; Bcl2, B-cell lymphoma 2; BDNF, brain-derived neurotrophic factor; BECN1, beclin-1; BMD, bone mineral density; BMI, body mass index; BUN, blood urea nitrogen; BPA, bisphenol A; BW, body weight; cAMP, cyclic adenosine monophosphate; CD8a, cluster of differentiation 8a; CDK1, and cyclin-dependent kinase 1; CKD, chronic kidney disease; CL, clinical; COL18A1, endostatin; COX-2, cyclooxygenase 2; CRE, cAMP-response element; CRP, C reactive protein; CSCs, cancer stem cells; CTGF, connective tissue growth factor; CTX, collagen type 1 cross-linked C telopeptide; βCTX, type I collagen crosslinked beta C-telopeptide; Cu/Zn SOD, copper/zinc superoxide dismutase; Conc., concentration; CREB, cAMP response element-binding protein; CXCL5, C-X-C motif chemokine 5; eNOS, endothelial nitric oxide synthase; ERK, extracellular-signal-regulated kinase; DA, daidzin; DN, diabetic nephropathic; DNMT1/3a, DNA methyltransferase DNA methyltransferase 1 and 3a; 6,8-DPN, 6,8-diprenylnaringenin; DPPH, 2,2–diphenyl–1-picrylhydrazyl radical; DSS, dextran sulfate sodium; DZ, daidzein; ER, estrogen; ERα, estrogen receptor alpha; ERβ, estrogen receptor beta; ERK, extracellular signal-regulated kinase; FADD, Fas-associated protein with death domain; FAS, fatty acid synthase; FasL, Fas ligand; FBG, fasting blood glucose; FoxM1, forkhead box protein M1; FOXO3a, forkhead box O3; Foxp3, forkhead box P3; FSD, female sexual dysfunction; GC, glycitin; GCRα/β, glucocorticoid receptor alpha and beta; GE, genistin; GK, Goto-Kakizaki; GLUT4, glucose transporter 4; 8-GN, 8-geranylnaringenin; G6PD, glucose-6-phosphate dehydrogenase; GRP78, glucose-regulated protein 78; GS, genistein; GSH, reduced glutathione; GST, GSH transferase; GT, glycitein; HbA1c, glycosylated hemoglobin; HCC, hepatocellular carcinoma; HDL, high-density lipoprotein; HFD, high-fat diet; HFs, hot flashes; H_2O_2, hydrogen peroxide; HOMA-IR, homeostatic model assessment of insulin resistance; HOTAIR, homebox transcript antisense RNA; HSP-70, heat shock protein 70; 5-HT, 5-hydroxytryptamine (or serotonin); 5-HT1A, serotonin 1A; HOX, homebox; ICR, institute cancer research; IBS, irritable bowel syndrome; ICAM-1, intracellular adhesion molecule 1; IDL, intermediate-density lipoprotein; IFN-γ, interferon gamma; IL, interleukin; IMQ, imiquimod; iNOS, inducible nitric oxide synthase; IP-10, interferon gamma-induced protein 10; IR, ischemia/reperfusion; IRE1α, inositol-requiring enzyme 1 alpha; ITGB3, integrin beta 3; JAK, Janus kinase; JNK, c-Jun N-terminal kinase; KLK4, kallikrein-related peptidase 4; LDH, lactase dehydrogenase; LDL, low-density lipoprotein; LPL, lipoprotein lipase; MAP, mitogen-activated protein; MAP2K2, mitogen-activated protein kinase kinase 2; MAPK, mitogen-activated protein kinase;

MDA, malondialdehyde; MDSC, myeloid-derived suppressor cell; ME, malic enzyme; miR-27a, microRNA-27a; MKK4, MAP kinase kinase 4; MMP-14, matrix metalloprotenase-14; Mn SOD, manganese superoxide dismutase; MPO, myeloperoxidase; mTORC1, mammalian target of rapamycin complex 1; MyD88, myeloid differentiation primary response 88; NAFLD, non-alcoholic fatty liver disease; NF-κB, nuclear factor kappaB; NHDL-C, non-high-density lipoprotein C; NO, nitric oxide; NOD, non-obese diabetic; NOS, nitric oxide synthase; OB, observed; OC, osteocalcin; OPG, osteoprotegerin; OVX, ovariectomized; PCa, prostate cancer; 6-PN, 6-prenylanaringenin; 8-PN, 8-prenylnaringenin; P1NP, procollagen 1 N-terminal propeptide; PCNA, proliferating cell nuclear antigen; p-CREB, phosphorylated CREB; PGK, phosphoglycerate kinase; PI3K, phosphoinositol 3-kinase; pI-kBα, phosphorylation of I-kBα; PPARγ, peroxisome proliferator-activated receptor-gamma; PR, progesterone receptor; pSTAT3, phosphorylated STAT3; PTEN, phosphatase and tensin homolog; RANKL, receptor activator for nuclear factor κB ligand; Ref, references; ROS, reactive oxygen species; SBP, systolic blood pressure; SCOP, scopolamine; SD, Sprague–Dawley; Shh, sonic hedgehog; 6,7,4–THIF, 6,7,4'-trihydroxyisoflavone; SIRT1, Sirtuin 1; SREBP-1c, sterol regulatory element-binding protein 1c; STAT, signal transducer and activator of transcription; TAK1, TGFβ-activated kinase 1; TC, total cholesterol; T2D, type 2 diabetes; TEWL, trans-epidermal water loss; TG, triglyceride; TGFβ, tumor growth factor beta; TH, type T helper; TIMP-2/3, tissue inhibitor of metalloproteinase 2 and 3; TLR4, toll-like receptor 4; TNFα, tumor necrosis factor alpha; TRAP, tartrate-resistant acid phosphatase; TrkB, tropomyosin receptor kinase B; Trx1, thioredoxin-1; UPR, unfolded protein response; UVA, ultraviolet A; UVB, ultraviolet B; VEGF-A, vascular endothelial growth factor A; VEGFR2, vascular endothelial growth factor receptor 2; VLDL, very-low-density lipoprotein.
